# Efficacy and safety of pharmacological interventions for the treatment of cocaine use disorder: A systematic review and network meta‐analysis

**DOI:** 10.1111/add.70486

**Published:** 2026-07-22

**Authors:** Craig Paterson, Thomas Parkhouse, Chloe Burke, Monika Halicka, Jennifer C. Palmer, Hend Gabr, Rebecca Wilson, Katie E. Webster, Francesca Spiga, Sarah Dawson, Deborah M. Caldwell, Jennifer Scott, Julian P. T. Higgins, Jelena Savović

**Affiliations:** ^1^ Population Health Sciences, Bristol Medical School University of Bristol Bristol UK; ^2^ National Institute for Health and Care Research (NIHR) Bristol Evidence Synthesis Group University of Bristol Bristol UK; ^3^ Department of Mathematics, Insurance and Statistics, Faculty of Business Menoufia University Menoufia Egypt; ^4^ National Institute for Health and Care Research (NIHR) Applied Research Collaboration West (ARC West) at University Hospitals Bristol and Weston NHS Foundation Trust Bristol UK

**Keywords:** cocaine use disorder, network meta‐analysis, pharmacotherapy, safety and acceptability, systematic review, treatment effectiveness

## Abstract

**Background and aims:**

Cocaine use disorder (CUD) is an increasingly widespread concern worldwide. Various pharmacological treatments have been investigated for CUD, but their efficacy remains unclear, and none have been licensed for treatment. Therefore, we assessed the comparative effectiveness, safety and acceptability of pharmacological interventions for the treatment of CUD and prevention of relapse.

**Methods:**

Systematic review and network meta‐analyses (PROSPERO: CRD42024596434) of double‐blind randomized controlled trials (RCTs) of pharmacological interventions specifically tested for the treatment of CUD with a minimum follow‐up of 4 weeks in any in/outpatient setting, with no restriction on geographic location. Participants included adults with diagnosed CUD. Participants with co‐occurring substance use disorders and/or common mental health conditions were eligible. We assessed risk of bias using the Cochrane Risk of Bias 2 tool and certainty of evidence was assessed using the Confidence in Network Meta‐analysis framework. We assessed 12 effectiveness, three acceptability and two safety outcomes. Our primary outcomes were continuous abstinence, point abstinence, dropout for any reason and serious adverse events. We also examined longest duration of continuous abstinence, extent of cocaine use, craving, severity of dependence, dropout due to adverse events, adherence and mortality.

**Results:**

We included 218 reports of 163 studies (14 871 participants) assessing 89 unique medications, grouped into 8 categories (agonist/replacement approaches; antidepressants and other serotonergic agents; glutamate modulators; anticonvulsants and GABAergic agents; antagonist/blocker approaches; opioid agonists and antagonists; combination pharmacotherapy; other neuroactive agents). Most study results had some concerns or high risk of bias, and the results of syntheses were generally judged to be very low certainty evidence. As such, the findings ought to be interpreted with caution. No single treatment type showed consistent beneficial effects across all effectiveness outcomes and there was limited evidence that results varied by intervention type or presence of co‐occurring comorbidity.

**Conclusions:**

In a comprehensive network meta‐analysis, we found no convincing evidence for the effectiveness of any pharmacological treatments for cocaine use disorder.

## INTRODUCTION

According to the UN World Drug Report 2025, an estimated 25 million individuals over the age of 15 years used cocaine in the past year, representing 0.5% of the global population [1]. Only a fraction will go on to experience cocaine use disorder (CUD), but for those that do, it is characterised by the compulsive use of cocaine despite the physical or psychological harm it may cause [2]. Diagnosis is typically established according to the American Psychiatric Association Diagnostic and Statistical Manual of Mental Disorders, Fifth Edition (DSM‐5), where an individual displays at least two of 11 physiological and behavioural symptoms [3]. Estimates suggest that 20% of individuals who use cocaine meet the criteria for, or may develop, the disorder within 2 years [4, 5]. Chronic use of cocaine is associated with increased all‐cause mortality, acute and chronic cardiovascular pathology [6], as well as neurological deficits such as impaired attention and working memory, and response inhibition [7]. At a societal level, cocaine use is associated with increased violence, crime, and the transmission of HIV and hepatitis C [8, 9].

Despite concerted efforts in this area, no standard care pathway for CUD exists, even as the demand for specialist treatment continues to rise [10]. A previous review of randomised controlled trials (RCTs) investigating the use of psychosocial and pharmacological interventions for CUD concluded that only contingency management (a psychosocial intervention) showed a significant reduction in cocaine use amongst adults [11]. While promising, psychosocial interventions may be limited in their ability to address the physical symptoms of CUD, such as cravings and withdrawal. Pharmacological interventions may have a role in treating CUD, as they have for other substance use disorders, such as alcohol use disorder [12, 13] and opioid use disorder [14]. However, despite promising results from individual RCTs investigating a wide variety of medications (e.g. anticonvulsants, antidepressants, psychostimulants), systematic reviews have failed to demonstrate clear or consistent benefits of pharmacological interventions for CUD [11, 15, 16].

Existing systematic reviews of multiple pharmacological treatments for CUD have varied substantially in their scope, outcomes examined and analytic approach. Chan *et al*. [15] summarised seven systematic reviews and 48 additional RCTs, presenting meta‐analyses for some outcomes but not for several clinically relevant outcomes. A later review conducted by Bentzley *et al*. [11] incorporated a larger evidence base but focused on a single outcome. In this context, no established recommendation for pharmacotherapy exists and no drugs have specific approval for use in the treatment of CUD. Given the multi‐dimensional nature of CUD and the limitations of prior reviews that have focused on a narrow set of outcomes and/or treatments, there remains a need for a comprehensive comparative evaluation of pharmacological treatments across multiple clinically relevant outcomes, using an up‐to‐date systematic review and network meta‐analysis (NMA) to enable a coherent comparison of the relative effects of the numerous treatment options.

### Aims and objectives

This review aimed to determine the comparative effectiveness, safety, and acceptability of different pharmacological interventions for the treatment of CUD and the prevention of relapse.

## METHODS

This review was pre‐registered with PROSPERO (CRD42024596434) and the National Institute for Health and Care Research (NIHR165378). Input from people with lived experience of cocaine use informed protocol development. This review is reported following PRISMA (Preferred Reporting Items for Systematic reviews and Meta‐Analyses) guidelines [17].

### Eligibility criteria

We sought RCTs with at least 4 weeks of follow‐up from baseline. To be eligible for inclusion, trial participants and personnel must have been blinded to intervention allocation, which may have been evidenced by the use of a placebo. Individually randomised or cluster‐randomised trials were eligible, as were trials with factorial and crossover designs. However, only data from the first period of a crossover trial were eligible. Studies reported only in conference abstracts, ongoing trial protocols and other ‘grey literature’ were ineligible.

#### Population

Eligible studies were of individuals aged ≥18 years with CUD or dependence (including crack cocaine), according to formal diagnostic criteria: DSM‐5, DSM‐IV or International Classification of Diseases (ICD) classification. For convenience, we use the term CUD throughout, which should be interpreted as including cocaine dependence. Studies of individuals diagnosed with cocaine abuse or where a significant majority (>80%) of participants were not diagnosed with CUD were excluded.

Participants with a co‐occurring substance use disorder (SUD) and those with common mental health conditions (e.g. depression) were eligible. However, studies in participants with co‐occurring amnestic, bipolar, delirium, dementia, dissociative or schizophrenic disorders were excluded. Studies of individuals who were in receipt of prescription stimulants within 6 months of trial participation, studies of opportunistic screening and treatment, and studies conducted solely in laboratory settings were also excluded.

#### Interventions

Any pharmacological intervention (including combinations) was eligible if it was used for the treatment of CUD. No restrictions were placed on intervention setting (in‐patient vs outpatient), treatment supervision, or presence of co‐occurring psychosocial interventions or support, provided that a direct comparison for the evaluation of the pharmacological intervention effect was available.

#### Comparators

Eligible comparators were placebos or any other eligible pharmacological intervention. Psychosocial interventions in isolation were not eligible comparators. Studies only comparing the effectiveness of different doses, formulations, release or delivery schedules of the same pharmacological agent, but with no distinct comparator, were included in the systematic review but not in the NMAs.

#### Outcomes and timepoints of interest

Eligible outcomes are detailed in Table 1 and include 12 effectiveness, three acceptability and two safety outcomes. Where studies reported multiple measures for a given outcome, we followed a hierarchy of preference or conducted a within‐trial synthesis to combine estimates, taking account of correlations between outcomes (Appendix S1). Where studies did not report outcomes and/or timepoints of interest, they were included in the systematic review but not in the NMAs.

**TABLE 1:** Review outcomes and time points of interest with definitions.

Outcome	Definition	Time point	Studies with outcome	Studies in NMA	Classes in NMA
Effectiveness					
Primary					
Continuous abstinence	Whether a participant was abstinent from cocaine use for a specified duration (confirmed using more than two consecutive tests) of 1 week or more at: (i) any point during the treatment period or through to the end of treatment; and (ii) the longest follow‐up. Only confirmed or verified outcomes were eligible (e.g. urinalysis); solely self‐reported measures were excluded	During treatment	50	46	9
		During follow‐up	5	5	4
Point abstinence	Whether the participant was abstinent at the time of assessment, based on confirmed or verified outcomes, as above	End of treatment	27	22	8
		End of follow‐up	7	7	7
Secondary					
Longest duration of continuous abstinence	Defined as the longest duration of continuous abstinence during the treatment period, based on self‐report and/or urinalysis	During treatment	20	19	6
Extent of use—frequency	How often participants self‐reported using cocaine in a given period	During treatment	54	40	9
		During follow‐up	8	7	5
Extent of use—quantity	How much cocaine participants used in a given period	During treatment	30	19	8
		During follow‐up	5	4	5
Extent of use—urine BE levels	Level of BE detected in participant’s urine as a measure of recent cocaine use	During treatment	27	12	7
		During follow up	4	0	0
Extent of use—proportion of UDS	Proportion of positive/negative urinary drug screenings during a given period	During treatment	62	45	8
		During follow‐up	2	0	0
Craving	As measured by any validated scale, at the end of treatment	End of treatment	83	39	8
Severity of dependence	Defined as a change in the number of formal CUD diagnostic criteria met by participants	End of treatment	1	0	0
Acceptability					
Primary					
Dropout from treatment for any reason	Defined as non‐completion of the full treatment period for any reason	End of treatment	141	135	9
Secondary					
Dropout owing to adverse events	Defined as non‐completion of the full treatment period owing to adverse events	End of treatment	57	39	9
Adherence	Defined as adherence to study medication protocol (rather than adherence to adjuvant therapy)	During treatment	67	52	9
Safety					
Primary					
Serious adverse events	As defined by the study authors	End of treatment	88	41	9
Secondary					
Mortality	From any cause	End of treatment	19	4	5

Abbreviations: BE, benzoylecgonine; CUD, cocaine use disorder; NMA, network meta‐analysis; UDS, urine drug screen.

### Searches and study selection

Eligible RCTs were identified in two stages. First, we searched MEDLINE‐Ovid (2 February 2024) for relevant systematic reviews from the preceding 5 years and created a list of all RCTs included in the eight reviews identified [11, 15, 16, 18, 19, 20, 21, 22]. Full‐text reports of all identified RCTs (*n* = 237) were assessed for eligibility by two reviewers independently, using the LaserAI platform (www.laser.ai). In a second stage 1 year later, we searched for primary studies in MEDLINE‐Ovid, PsycINFO‐Ovid and the Cochrane Central Register of Controlled Trials (CENTRAL) (2 February 2025). The full search strategies are presented in Appendix S2. No date restrictions were applied to this search, although the results were restricted to reports published in English. Primary study search results were screened by two reviewers independently. Discrepancies at any stage were resolved by discussion with a third reviewer or with the wider review team.

### Data extraction and processing

Standardised forms, created and piloted in LaserAI, were used to extract study information by a single reviewer, and were then cross‐checked in detail by a second reviewer. Discrepancies were resolved by discussion with a third reviewer or with the wider review team. During extraction, AI model suggestions from LaserAI were used, with all suggestions adjudicated by a human reviewer.

For dichotomous outcomes, we extracted arm‐level counts of participants with event, number of participants with available outcome data, number of participants retained at end of treatment and number of participants randomised into each arm. The choice of denominator used for each analysis depended on the type of outcome. For safety and acceptability outcomes, the denominator was the number of participants randomised. For effectiveness outcomes, where it was reported, the number of participants included in the analyses was used as the denominator, otherwise we used the number of participants who completed treatment.

An extraction hierarchy was established for continuous outcomes. Following National Institute for Health and Care Excellence (NICE) Technical Support Unit (TSU) guidance [23], continuous outcome data were extracted according to a hierarchy with a preference for means and standard deviations of change from baseline (CfB). For multi‐arm trials including two or more of the same medication (e.g. two different doses of the same drug), we pooled the outcome data into a single arm for subsequent analysis before including them in NMAs (for details, see Appendix S3). This approach prevents double‐counting participants and avoids including correlated comparisons in the analyses.

### Risk of bias assessment

We assessed risk of bias using the Cochrane Risk of Bias 2 tool (RoB2) [24] at the results level for all outcomes included in quantitative synthesis. Risk of bias was assessed by a single reviewer and checked in detail by a second reviewer. Discrepancies were resolved by discussion with a third reviewer or with the wider review team.

### Synthesis of results

We conducted an NMA, allowing the relative effects of all pharmacological interventions to be compared simultaneously in a single analysis [25]. We fitted all NMA models in a Bayesian framework using the multinma package [26] in R 4.5.1 [27]. In contrast to a frequentist framework, where intervention effects are commonly reported using point estimates, 95% confidence intervals and *P*‐values, Bayesian approaches produce point estimates, credible intervals and probability statements about intervention effects. These reflect uncertainty in the available evidence and can be interpreted probabilistically as the degree of belief that intervention effects exceed clinically meaningful thresholds (for a plain‐language explanation, see Appendix S4).

Random‐effects models were used as the primary approach, assuming a common between‐study heterogeneity standard deviation (SD), which we denote as *τ*, with common‐effect models fitted as sensitivity analyses. Before undertaking the synthesis, we assessed transitivity by examining the similarity of the distribution of potential effect modifiers across the comparisons in the network. Potential effect modifiers included intervention duration, treatment setting, age and co‐occurring SUD. Global consistency was examined by comparing the model fit between a model assuming consistency and a model allowing for inconsistency. If there was evidence of inconsistency between direct and indirect evidence, we planned further evaluation using a local loop‐based approach (node‐splitting) [23]. Full analysis details, including specific priors, are detailed in Appendix S4.

To aid interpretation, we re‐expressed standardised mean difference (SMD) results as mean differences (MDs), based on the most commonly reported measure for a given outcome, which is articulated under each relevant subsection (full details of the methods used for conversions and reinterpretations are presented in Appendix S3). As such, results are expressed as MDs for continuous outcomes and ORs for dichotomous outcomes, with 95% credible intervals (95% CrIs). Point estimates of relative effect represent the 50% quantile (median) of the posterior distribution, and the 95% CrI represents its 2.5% and 97.5% quantiles.

#### Sensitivity and subgroup analyses

We planned to explore several potential study‐level effect modifiers, including: intervention type (i.e. studies designed to initiate abstinence vs those designed to prevent relapse); presence of co‐occurring SUD/comorbidity; predominant cocaine type used; presence of co‐interventions (e.g. counselling or other psychosocial interventions); and setting and delivery of interventions (in‐patient vs outpatient, supervised vs unsupervised). These were conducted on primary outcomes only, using a meta‐regression approach to estimate intervention–effect modifier interactions (Appendix S4).

#### Certainty of evidence

We assessed the certainty of the evidence using the CINeMA (Confidence in Network Meta‐Analysis) framework [28], which covers the six domains of study‐specific risk of bias, publication bias, indirectness, imprecision, heterogeneity and incoherence. Assessments were completed by one reviewer and checked thoroughly by a second. Any discrepancies were resolved by discussion with a third reviewer or with the wider review team. In the absence of established thresholds for minimum clinically important differences (MCIDs), we used conservative estimates of SMD = −0.2 or +0.2, and OR < 0.8 or OR > 1.25, in line with our previous work. Our judgements of the overall certainty of evidence were reached by starting at high certainty and downgrading by one level for each domain with some concerns, and by two levels for each domain with major concerns.

## RESULTS

### Included studies

We included 218 reports of 163 studies, including 14 871 participants (Figure 1). In total, 151 studies contributed data to at least one NMA. Eight [29, 30, 31, 32, 33, 34, 35, 36] of the remaining 12 studies provided narrative outcomes only and four studies [37, 38, 39, 40] contributed no outcomes to this review. Characteristics of the included studies are presented in Table 2. The sample size of studies ranged between 14 and 464 participants (median = 81 participants). Studies predominantly recruited mixed‐sex samples, although one study recruited only females and 19 recruited only males. The mixed samples were predominantly male (median = 76%, range = 48%–98%) and the average age ranged from 28 to 52 years (median = 39 years). Treatment duration ranged from 1 day to 34 weeks, with a median duration of 12 weeks. Most studies combined pharmacological treatments with adjunctive therapy (95%).

**FIGURE 1:**
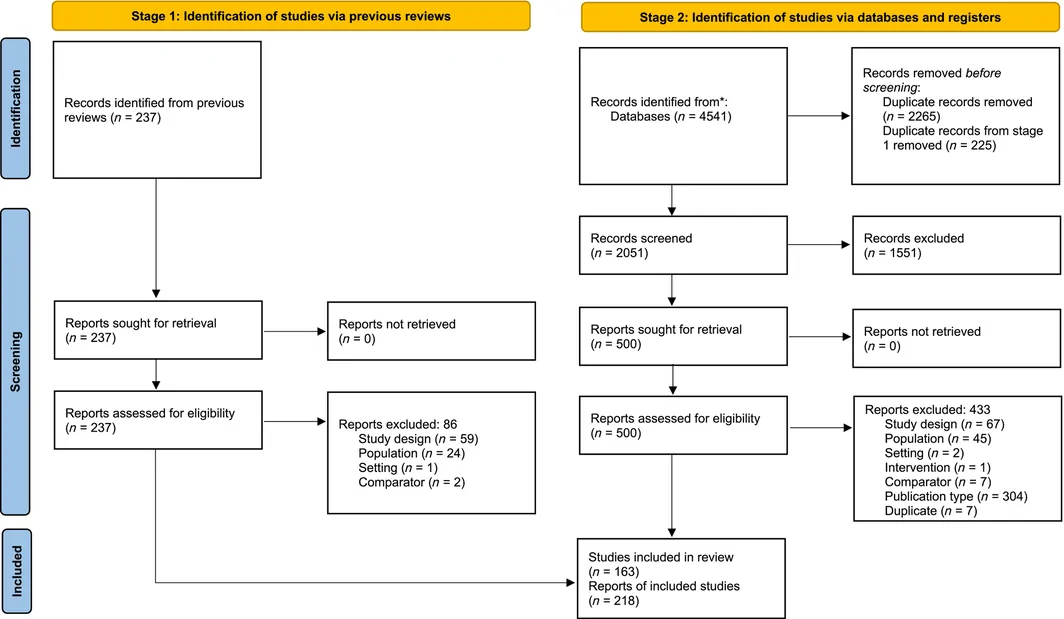
PRISMA (Preferred Reporting Items for Systematic reviews and Meta‐Analyses) flow diagram.

**TABLE 2:** Characteristics of included studies.

Study;^a^ country	*n* randomised^b^ (per arm); *n* treated^c^ (per arm)	Age in years, mean (SD), range; sex (% male); ethnicity^d^	Co‐dependencies; comorbidities^e^	Primary cocaine type (route of administration)	Intervention^f^ (dose^g^, category^h^); (intervention type^i^, duration^j^, setting, supervision^k^, adjuvant therapy)	Outcomes measured
Afshar 2012 [41]; USA	NR (NR, NR); 24 (11, 13)^l^	45.64 (6.33); 70.8% male; Black 79.2% (*n* = 19); White 8.3% (*n* = 2); Other 12.5% (*n* = 3)^m^	No co‐dependencies; HAM‐D score of ≥12 and a diagnosis of major depression, dysthymic disorder or substance‐induced mood disorder	Crack (100% smoked)	(1) Mirtazapine (15 mg 3×/day, MA‐AD/ANTID) (2) Placebo (AI, 12 weeks, outpatient, unsupervised, RP)	NMA: extent of use (BE level; EoT); craving; adherence Narrative: serious adverse events; extent of use (frequency; EoT)
Alterman 1992 [42]; USA	42 (21, 21); NR (NR, NR)	35 (NR); 100% male; 90% Black	No co‐dependencies; No comorbidities	NR (NR)	(1) Amantadine (100 mg 2×/day, NMDAR‐ANT/GLUT) (2) Placebo (AI, 11 days, outpatient, unsupervised, other PSI)	NMA: dropout (any reason); point abstinence (follow‐up); extent of use (frequency; follow‐up) Narrative: serious adverse events
Anderson 2009 [43]; USA	210 (69, 69,72); 207 (68, 68,71)	42.38 (8.3); 71.5% male; Race: White 39.1% (*n* = 81); African American 56.0% (*n* = 116); American Indian or Alaska Native 1.0% (*n* = 2); other 3.4% (*n* = 7); missing 0.5% (*n* = 1) Ethnicity: Hispanic or Latino 14.0% (*n* = 29); not Hispanic or Latino 86.0% (*n* = 178)^m^	No co‐dependencies; No comorbidities	NR (NR)	(1) Modafinil (200 mg 1×/day, STIM/ARA) (2) Modafinil (400 mg 1×/day, STIM/ARA) (3) Placebo (AI, 12 weeks, outpatient, unsupervised, CBT)	NMA: dropout (any reason); dropout (adverse events); LDCA Narrative: extent of use (BE level; EoT); craving
Baldacara 2013 [44]; Brazil	30 (15, 15); NR (NR, NR)	28.6 (4.4); 100% male; NR^n^	No co‐dependencies; No comorbidities	Crack (NR)	(1) Disulfiram (250 mg 1×/day, Disulfiram/ABA) (2) Placebo (AI, 60 days, NR, NR, motivational + other PSI)	NMA: dropout (any reason); point abstinence (EoT); extent of use (frequency; EoT); extent of use (quantity; EoT) Narrative: none
Baldacara 2016 [45]; Brazil	60 (30, 30); 60 (30, 30)	29.45 (4.37); 100% male; NR	No co‐dependencies; No comorbidities	Crack (100% smoked)	(1) Topiramate (25–100 mg 2×/day, ACV + MS/ACV + GABA) (2) Placebo (AI, 12 weeks, outpatient, unsupervised, motivational + other PSI)	NMA: dropout (any reason); point abstinence (EoT); extent of use (frequency; EoT); extent of use (quantity; EoT) Narrative: none
Batki 1996 [46]; USA	32 (16, 16); 32 (16, 16)	34.35 (7.65); 65.7% male; African American 53.1% (*n* = 17); White 21.9% (*n* = 7); Hispanic 15.6% (*n* = 5); Other 9.4% (*n* = 3)	No co‐dependencies; No comorbidities	Mixed (97% smoked crack; 3% intravenous cocaine)	(1) Fluoxetine (20 mg 2×/day, SSRIs+OSA/ANTID) (2) Placebo (AI, 12 weeks, outpatient, unsupervised, other PSI)	NMA: dropout (any reason); dropout (adverse events) Narrative: none
Becker 2020 [47]; USA	24 (NR, NR); 21 (12, 9)	41.44 (8.24, 25–60); 76.2% male; White 14.3% (*n* = 3); Black 71.4% (*n* = 15); Hispanic 4.8% (*n* = 1); Native American 4.8% (*n* = 1); NR 4.8% (*n* = 1)^m^	No co‐dependencies; No comorbidities	NR (NR)	(1) Lidocaine (2 mg/kg Nonex/hour, Lidocaine/ONA) (2) Placebo (RP, 1 day, in‐patient, supervised, CBT + CM)	NMA: extent of use (frequency; follow‐up); extent of use (quantity; follow‐up) Narrative: serious adverse events; extent of use (proportion tests/days; follow‐up)
Berger 2005 [48]; USA	60 (15, 15, 15, 15); 60 (15, 15, 15, 15)	39.1 (6.6); 70% male; African American 92%; Caucasian 8%	No co‐dependencies; No comorbidities	NR (93% smoked; 5% intravenous; 2% intranasal)	(1) Gabapentin (1800 mg 1×/day, Gabapentin/ACV + GABA) (2) Lamotrigine (150 mg 1×/day, ACV + MS/ACV + GABA) (3) Reserpine (0.5 mg 1×/day, Reserpine/ABA) (4) Placebo (AI, 8 weeks, outpatient, unsupervised, CBT)	NMA: dropout (any reason); dropout (adverse events); serious adverse events; extent of use (BE level; EoT); extent of use (frequency; EoT); craving; adherence Narrative: none
Bisaga 2006 [49]^5^ (Bisaga 2005 [50]); USA	99 (51, 48); NR (NR, NR)	39 (7.96, 22–58); 88% male; 46% African American; 26% Caucasian; 20% Hispanic; 8% Other	No co‐dependencies; No comorbidities	NR (NR)	(1) Gabapentin (1600 mg 2×/day, Gabapentin/ACV + GABA) (2) Placebo (AI, 12 weeks, outpatient, unsupervised, CBT + motivational)	NMA: serious adverse events; continuous abstinence (treatment); extent of use (proportion tests/days; EoT) Narrative: dropout (any reason); craving
Bisaga 2010 [51]; USA	81 (39, 42); NR (NR, NR)	40 (7.9); 79% male; 37.0% Black; 18.5% Hispanic; 40.7% White; 3.7% Other	No co‐dependencies; No comorbidities	NR (61.7% snorted; 38.3% smoked)	(1) Memantine (20 mg 2×/day, NMDAR‐ANT/GLUT) (2) Placebo (AI, 14 weeks, unsupervised, CBT + CM + motivational)	NMA: dropout (any reason); serious adverse events; continuous abstinence (treatment); adherence Narrative: extent of use (proportion tests/days; EoT); craving
Blevins 2021 [52]; USA	117 (55, 62); 108 (53, 55)	46.2 (8.5); 61.1% male; 78.7% of African ancestry; 19.4% of European ancestry; 1.9% Other^m^	No co‐dependencies; No comorbidities	NR (NR)	(1) Ondansetron (4 mg 2×/day, Ondansetron/ANTID) (2) Placebo (AI, 9 weeks, outpatient, unsupervised, CBT + other PSI)	NMA: dropout (any reason); serious adverse events; extent of use (proportion tests/days; EoT); adherence Narrative: mortality
Brady 2002 [53]; USA	177 (38, 52, 39, 48); 139 (30, 42, 27, 40)	31.86 (6.28); 63.3% male; Minority 61.9% (*n* = 86)^m^	No co‐dependencies; No comorbidities	NR (NR)	(1) Carbamazepine (‘affective’) (400 mg 2×/day, ACV + MS/ACV + GABA) (2) Carbamazepine (‘non‐affective’) (400 mg 2×/day, ACV + MS/ACV + GABA) (3) Placebo (‘affective’) (4) Placebo (‘non‐affective’) (M, 12 weeks, outpatient, unsupervised, no adjuvant therapy)	NMA: extent of use (frequency; EoT); craving; adherence Narrative: none
Brodie 2009 [54]; Mexico	128 (NR, NR); 103 (50, 53)	29.08 (6.32); 96.1% male; NR^m^	No co‐dependencies; No comorbidities	NR (NR)	(1) Vigabatrin (1.5 g 2×/day, ACV + MS/ACV + GABA) (2) Placebo (AI, 9 weeks, outpatient, mixed, CBT)	NMA: dropout (any reason); continuous abstinence (treatment); continuous abstinence (follow‐up); extent of use (proportion tests/days; EoT) Narrative: serious adverse events; point abstinence (EoT); extent of use (frequency; EoT); extent of use (quantity; EoT); craving
Campbell 2003 [55] (Campbell 1994 [56]); USA	146 (47, 49, 50); NR (NR, NR, NR)	33.44 (NR); 69.9% male; 16.4% White; 81.5% Black; 1.4% Other	No co‐dependencies; No comorbidities	Crack (NR)	(1) Carbamazepine (800 mg 1×/day, ACV + MS/ACV + GABA) (2) Desipramine (200 mg 1×/day, MA‐AD/ANTID) (3) Placebo (AI, 8 weeks, outpatient, unsupervised, NR)	NMA: dropout (any reason); continuous abstinence (treatment); extent of use (frequency; EoT) Narrative: dropout (adverse events)
Carpenter 2024 [57]; USA	86 (45, 41); 84 (43, 41)	44.7 (9.9); 82.6% male; Race: Black 43.0% (37/86); White 45.3% (39/86); Asian 4.7% (4/86); Other 4.7% (4/86) Ethnicity: Hispanic 23.3% (20/86)	No co‐dependencies; No comorbidities	NR (NR)	(1) MAS‐ER‐extended release (80 mg 1×/day, STIM/ARA) (2) Placebo (AI, 11 weeks, outpatient, unsupervised, CM + other PSI)	NMA: dropout (any reason); serious adverse events; continuous abstinence (treatment) Narrative: point abstinence (EoT); adherence
Carroll 1994 [58] (Carroll 1994b [59]; Carroll 1995 [60]); USA	139 (NR, NR, NR, NR); NR (NR, NR, NR, NR)	28.8 (5.8); 73% male; 59 minority (54%)^m^	No co‐dependencies; No comorbidities	NR (62% freebase; 29% intranasal; 9% intravenous)	(1) Desipramine + RP (300 mg 1×/day, MA‐AD/ANTID) (2) Desipramine + clinical management (300 mg 1×/day, MA‐AD/ANTID) (3) Placebo + RP (4) Placebo + clinical management (AI, 12 weeks, outpatient, unsupervised, CBT + other PSI)	NMA: LDCA; extent of use (proportion tests/days; EoT) Narrative: none
Carroll 2004 [61]; USA	121 (30, 30, 30, 31); NR (NR, NR, NR)	34.6 (7); 73.6% male; African American 31% (*n* = 38); Hispanic 6% (*n* = 7); White 63% (*n* = 76)	No co‐dependencies; No comorbidities	NR (74% freebase users)	(1) Disulfiram + CBT (250 mg 1×/day, disulfiram/ABA) (2) Disulfiram + interpersonal psychotherapy (250 mg 1×/day, Disulfiram/ABA) (3) Placebo + CBT (4) Placebo + interpersonal psychotherapy (AI, 12 weeks, outpatient, unsupervised, CBT + other PSI)	NMA: extent of use (frequency; EoT); adherence Narrative: serious adverse events; mortality; extent of use (proportion tests/days; EoT)
Carroll 2012 [62]; USA	112 (29, 30, 27, 26); 107 (27, 28, 26, 26)	38.3 (7.5); 58.9% male; Caucasian 64.3% (*n* = 72); African American 22.3% (*n* = 25); Hispanic 13.4% (*n* = 15)	All participants likely opioid dependent (all enrolled in a methadone programme); No comorbidities	NR (NR)	(1) Disulfiram + Twelve Steps (250 mg 1×/day, disulfiram/ABA) (2) Disulfiram (250 mg 1×/day, disulfiram/ABA) (3) Placebo + Twelve Steps (4) Placebo (AI, 12 weeks, outpatient, NR, other PSI + methadone)	NMA: dropout (any reason); extent of use (frequency; EoT); extent of use (frequency; follow‐up) Narrative: mortality
Carroll 2016 [63] (DeVito 2017 [64]); USA	99 (23, 28, 22, 26); 80 (20, 24, 18, 18)	39.3 (7.5); NR% male; Caucasian 39.4% (*n* = 39); African American 49.5% (*n* = 49); Latino 7.1% (*n* = 7); Multiracial/other 4% (*n* = 4)	No co‐dependencies; No comorbidities	NR (NR)	(1) Disulfiram + CM (250 mg 1×/day, disulfiram/ABA) (2) Disulfiram (250 mg 1×/day, disulfiram/ABA) (3) Placebo + CM (4) Placebo (AI, 12 weeks, outpatient, mixed, CBT + CM)	NMA: dropout (any reason); serious adverse events; continuous abstinence (treatment); extent of use (proportion tests/days; EoT); extent of use (frequency; EoT); extent of use (frequency; follow‐up); adherence Narrative: none
Carroll 2018 [65] (McCurdy 2024 [66]); USA	120 (28, 27, 38, 27); 117 (27, 26, 38, 26)	38.4 (9.4); 66.7% male; Caucasian 51.7% (*n* = 62); African American 20.9% (*n* = 25); Hispanic 26.8% (*n* = 32); Multiracial/other 0.9% (*n* = 1)	Individuals stabilised on methadone maintenance; No comorbidities	NR (NR)	(1) Galantamine + CBT (8 mg 1×/day, AChEIs/ONA) (2) Galantamine (8 mg 1×/day, AChEIs/ONA) (3) Placebo + CBT (4) Placebo (AI, 12 weeks, outpatient, supervised, CBT + methadone)	NMA: dropout (any reason); extent of use (frequency; EoT); adherence Narrative: serious adverse events
Ciraulo 2005a [67] S1 (Streeter 2005 [68]); USA	NR (NR, NR, NR, NR); 65 (16, 16, 17, 16)	40.35 (7.02); 72.3% male; White 21.5% (*n* = 14); Black 75.4% (*n* = 49); Other 3.1% (*n* = 2)^m^	No co‐dependencies; No comorbidities	NR (NR)	(1) Paroxetine (20 mg 2×/day, SSRIs+OSA/ANTID) (2) Pentoxifylline (400 mg 1×/day, Pentoxifylline/ONA) (3) Riluzole (50 mg 3×/day, NNM‐GluM/GLUT) (4) Placebo (AI, 8 weeks, outpatient, unsupervised, CBT)	NMA: dropout (any reason); extent of use (frequency; EoT); craving; extent of use (proportion tests/days; EoT)^o^ Narrative: serious adverse events
Ciraulo 2005a [67] S2 (Streeter 2005 [68]); USA	NR (NR, NR, NR); 60 (20, 20, 20)	43.03 (7.69); 71.7% male; White 6.7% (*n* = 4); Black 91.7% (*n* = 55); Other 1.7% (*n* = 1)^m^	No co‐dependencies; No comorbidities	NR (66.7%–100% crack users)	(1) Pramipexole (0.5 mg 3×/day, DA/ARA) (2) Venlafaxine (50 mg 3×/day, MA‐AD/ANTID) (3) Placebo (AI, 8 weeks, outpatient, unsupervised, CBT)	NMA: dropout (any reason); extent of use (frequency; EoT); craving; extent of use (proportion tests/days; EoT)^o^ Narrative: serious adverse events
Ciraulo 2005b [69]; USA	69 (34, 35); NR (NR, NR)	40.4 (7.6); 71% male; Black 48/69 (69.6%); White 19/69 (27.5%); Other (2.9%)2/69	No co‐dependencies; Depressed mood symptom rating of HAM‐D score ≥12. Subjects with Axis 1 diagnosis of major depression, anxiety disorder or dysthymic disorder included	Mixed [82.6% smoked (crack); 14.5% intranasal; 2.9% intravenous]	(1) Nefazodone (200 mg 2×/day, SSRIs+OSA/ANTID) (2) Placebo (AI, 8 weeks, outpatient, unsupervised, other PSI)	NMA: dropout (any reason); dropout (adverse events); extent of use (BE level; EoT); extent of use (frequency; EoT); craving Narrative: serious adverse events
Cornish 1995 [70]; USA	95 (NR, NR); NR (NR, NR)	36.07 (5.36); 98.8% male; 89% African American	No co‐dependencies; No comorbidities	NR (NR)	(1) Carbamazepine (individually titrated to plasma level of 4–12 mcg/ml, ACV+MS/ACV+GABA) (2) Placebo (AI, 10 weeks, outpatient, mixed, other PSI)	NMA: dropout (any reason); serious adverse events; mortality; extent of use (BE level; EoT); extent of use (proportion tests/days; EoT); adherence Narrative: craving
Cornish 2001 [71] (Ehrman 1996 [72]); USA	80 (40, 40); NR (NR, NR)	37.7 (5.97); 100% male; African American 78 (97.5%); Hispanic 2 (2.5%)^p^	No co‐dependencies; No comorbidities	NR (NR)	(1) Ritanserin (10 mg 1×/day, SSRIs+OSA/ANTID) (2) Placebo (AI, 4 weeks, outpatient, unsupervised, other PSI)	NMA: dropout (any reason); extent of use (proportion tests/days; EoT); craving; adherence^q^ Narrative: none
Covi 1995 [29]; USA	NR (NR, NR, NR, NR); 45 (10, 11, 10, 14)	30 (4.9); 80% male; 55.5% White 44.4% Black^m^	No co‐dependencies; No comorbidities	Mixed (42.2% smoking; 33.3% intranasal; 22.2% intravenous; 2.2% oral ingestion)	(1) Fluoxetine (20 mg 1×/day, SSRIs+OSA/ANTID) (2) Fluoxetine (40 mg 1×/day, SSRIs+OSA/ANTID) (3) Fluoxetine (60 mg 1×/day, SSRIs+OSA/ANTID) (4) Placebo (12.5 mg 1×/day, placebo/PLACEBO) (AI, 12 weeks, outpatient, unsupervised, other PSI)	NMA: none Narrative: serious adverse events; point abstinence (EoT); extent of use (quantity; EoT); craving
Dackis 2005 [73]; USA	62 (30, 32); NR (NR, NR)	44.5 (8.7, 25–63); 71% male; 80.6% African American	No co‐dependencies; No comorbidities	Mixed [87.1% crack (smoking)]	(1) Modafinil (400 mg 1×/day, STIM/ARA) (2) Placebo (AI, 8 weeks, outpatient, unsupervised, CBT)	NMA: dropout (any reason); continuous abstinence (treatment); extent of use (proportion tests/days; EoT) Narrative: dropout (adverse events); extent of use (frequency; EoT); extent of use (quantity; EoT); craving
Dackis 2012 [74]; USA	210 (70, 65, 75); NR (NR, NR, NR)	44.5 (8.7, 22–60); 74.8% male; 78.6% African American	No co‐dependencies; No comorbidities	Mixed [78.6% crack (smoking)]	(1) Modafinil (400 mg 1×/day, STIM/ARA) (2) Modafinil (200 mg 1×/day, STIM/ARA) (3) Placebo (AI, 8 weeks, outpatient, unsupervised, CBT)	NMA: dropout (any reason); continuous abstinence (treatment); point abstinence (EoT) Narrative: craving
Dakwar 2019 [75]; USA	55 (27, 28); NR (NR, NR)	47 (9.3); 74.5% male; Race: African American 61.8% (*n* = 34); Caucasian 12.7% (*n* = 7); more than one race 18.2% (*n* = 10); unknown/NR 7.3% (*n* = 4) Ethnicity: Hispanic 20% (*n* = 11)	No co‐dependencies; No comorbidities	Powder (63.6% freebase; 23.6% intranasal; 12.7% both)	(1) Ketamine (0.5 mg/kg 1× single dose, NMDAR‐ANT/GLUT) (2) Placebo (midazolam) (0.025 mg/kg 1x single dose, placebo/PLACEBO) (RP, 1 day, mixed, supervised, RP)	NMA: dropout (any reason); continuous abstinence (follow‐up) Narrative: dropout (adverse events); serious adverse events; mortality; craving
Delucchi 2002 [37]; USA	149 (NR, NR); NR (NR, NR)	38.3 (5.96); 66.2% male; African American 62.0% (*n* = 44)^s^	No co‐dependencies; No comorbidities	NR (NR)	(1) Fluoxetine (40 mg 1x/day, SSRIs+OSA/ANTID) (2) Placebo (NA, 12 weeks, outpatient, NR, NR)	NMA: none Narrative: none
DeVito 2019 [76]; USA	93 (30, 31, 32); 81 (26, 28, 27)	46.32 (7.66); 67.7% male; African American *n* = 62 (66.7%); Caucasian *n* = 12 (12.9%); Hispanic *n* = 15 (16.1%); Multiracial *n* = 4 (4.3%)	No co‐dependencies; No comorbidities	NR (NR)	(1) Galantamine (16 mg 1 x/day, AChEIs/ONA) (2) Galantamine (8 mg 1×/day, AChEIs/ONA) (3) Placebo (AI, 12 weeks, outpatient, mixed, CM + other PSI)	NMA: dropout (any reason); LDCA; extent of use (frequency; EoT); adherence Narrative: serious adverse events; mortality
DeVito 2022 [77]; USA	91 (26, 19, 19, 27); 89 (26, 19, 19, 25)	38.31 (10.81); 63.7% male; 64.8% White (59/91); 17.6% Black (16/91); 17.6% Latine/Hispanic (16/91)	Opioid dependent; No comorbidities	NR (NR)	(1) Modafinil + CM (400 mg 1×/day, STIM/ARA) (2) Modafinil (400 mg 1×/day, STIM/ARA) (3) Placebo + CM (4) Placebo (AI, 11 weeks, outpatient, mixed, CBT + CM + methadone)	NMA: dropout (any reason); dropout (adverse events); LDCA; extent of use (proportion tests/days; EoT); extent of use (frequency; EoT); adherence Narrative: none
Dieckmann 2014 [78]; Brazil	111 (56, 55); 111 (56, 55)	32.11 (7.12); 100% male; 100% Hispanic/Latino	No co‐dependencies; No comorbidities	Mixed [55% cocaine (presumed powder); 24.3% crack; 20.7% both]	(1) Biperiden (2 mg 3×/day, mAChR‐Ant/ONA) (2) Placebo (AI, 12 weeks, outpatient, unsupervised, CBT)	NMA: dropout (any reason); extent of use (frequency; EoT); extent of use (quantity; EoT); craving Narrative: dropout (adverse events); serious adverse events; mortality
Dursteler‐Macfarland 2013 [79]; Switzerland	62 (15, 15, 17, 15); 62 (15, 15, 17, 15)	35.92 (6.03); 64.5% male; NR	Heroin dependent; No comorbidities	NR (NR)	(1) Methylphenidate + CBT (30 mg 2×/day, STIM/ARA) (2) Methylphenidate (30 mg 2×/day, STIM/ARA) (3) Placebo + CBT (4) Placebo (AI, 12 weeks, NR, supervised, CBT + diacetylmorphine)	NMA: dropout (any reason); continuous abstinence (treatment); extent of use (frequency; EoT); extent of use (quantity; EoT) Narrative: none
Elkashef 2006 [80]; USA	300 (150, 150); 299 (149, 150)	40.7 (7.4); 78% male; African American 62.6% (*n* = 188); Caucasian 26.6% (*n* = 80); Hispanic 9% (*n* = 27); Other 1.6% (*n* = 5)	No co‐dependencies; No comorbidities	NR (86% smoked; 10% intranasal; 4% other)	(1) Selegiline Transdermal System [20mg 1×/day (transdermal continuous release), MA‐AD/ANTID] (2) Placebo (AI, 8 weeks, outpatient, mixed, other PSI)	NMA: dropout (any reason); serious adverse events; continuous abstinence (treatment); LDCA; extent of use (proportion tests/days; EoT); craving Narrative: none
Epstein 2009 [30]; USA	168 (30, 31, 21, 33, 32, 21); NR (NR, NR, NR, NR, NR, NR)	32.3 (7.8); NR% male;	Heroin dependent; No comorbidities	NR (NR)	(1) Methadone + vouchers for cocaine and opiate abstinence (100 mg 1×/day, OAT/OAA) (2) Methadone + vouchers for cocaine abstinence (100 mg 1×/day, OAT/OAA) (3) Methadone + non‐contingent vouchers (100 mg 1×/day, OAT/OAA) (4) Methadone + vouchers for cocaine and opiate abstinence (70 mg 1×/day, OAT/OAA) (5) Methadone + vouchers for cocaine abstinence (70 mg 1×/day, OAT/OAA) (6) Methadone + non‐contingent vouchers (70 mg 1×/day, OAT/OAA) (AI, 12 weeks, outpatient, NR, other PSI)	NMA: none Narrative: dropout (any reason); point abstinence (EoT)
Gawin 1989 [81] (Kosten 1992b [82]); USA	72 (31, 37, 32); NR (NR, NR, NR)	29.07 (1.61); 76% male; 71% White; 29% non‐White	No co‐dependencies; No comorbidities	NR (50% intranasal; 31.9% freebase; 18.1% intravenous)	(1) Desipramine (individual titration, dose of 2.5 mg/kg to achieve target plasma range of 140–220 ng/ml, MA‐AD/ANTID) (2) Lithium carbonate (individual titration to achieve plasma level of 0.6‐0.9 mEq/l, ACV+MS/ACV+GABA) (3) Placebo (AI, 6 weeks, outpatient, unsupervised, other PSI)	NMA: dropout (any reason); continuous abstinence (treatment); continuous abstinence (follow‐up); extent of use (quantity; EoT); extent of use (quantity; follow‐up); craving Narrative: none
George 2000 [83]; USA	20 (11, 9); NR (NR, NR)	37.9 (6.1); 60% male; 75% White	Opiate dependent; No comorbidities	NR (NR)	(1) Disulfiram (250 mg 1×/day, disulfiram/ABA) (2) Placebo (AI, 12 weeks, outpatient, NR, other PSI + buprenorphine)	NMA: dropout (any reason); continuous abstinence (treatment); extent of use (proportion tests/days; EoT); extent of use (quantity; EoT) Narrative: serious adverse events
Gilgun‐Sherki 2016 [84]; USA| Spain	208 (70, 69, 69); 205 (70, 68, 67)	47.1 (7.82, 26–60); 67.8% male; Race: Black 78% (*n* = 163); White 16% (*n* = 33); Other 5% (*n* = 11); missing = 1; Ethnicity: not Hispanic or Latino 91% (*n* = 189); Hispanic or Latino 9% (*n* = 19)	No co‐dependencies; No comorbidities	NR (77% smoking; 23% snorting)	(1) TV‐1380 (150 mg 1×/week, Coc‐PK/ABA) (2) TV‐1380 (300 mg 1×/week, Coc‐PK/ABA) (3) Placebo (AI, 12 weeks, outpatient, supervised, CBT + CM)	NMA: dropout (any reason); dropout (adverse events); serious adverse events; continuous abstinence (treatment) Narrative: none
Gonzalez 2003 [85]; USA	45 (15, 15, 15); NR (NR, NR, NR)	38 (6.5); 77.8% male; 75.6% Caucasian	Opioid dependent; No comorbidities	NR (over 67% smoked crack cocaine)	(1) Tiagabine (12 mg 2×/day, ACV + MS/ACV + GABA) (2) Tiagabine (6 mg 2×/day, ACV + MS/ACV + GABA) (3) Placebo (AI, 9 weeks, outpatient, mixed, other PSI + methadone)	NMA: dropout (any reason); extent of use (proportion tests/days; EoT); extent of use (frequency; EoT) Narrative: dropout (adverse events)
Gorelick 2006 [86]^39^; USA	70 (35, 35); 70 (35, 35)	34.1 (6.77); 100% male; 85.7% African American	No co‐dependencies; No comorbidities	NR (predominantly smoked)	(1) Bromocriptine (2.5 mg 3×/day, DA/ARA) (2) Placebo (RP, 26 weeks, mixed, NR, other PSI)	NMA: dropout (any reason); dropout (adverse events); adherence Narrative: craving
Grabowski 1995 [87] S1; USA	21 (11, 10); 21 (11, 10)	39.3 (5.03); 75.5% male; 38.7% Black; 42.8% White; 18.7% Hispanic; 0% Asian^m^	Opiate dependent; No comorbidities	NR (NR)	(1) Fluoxetine (20 mg 1×/day, SSRIs+OSA/ANTID) (2) Placebo (AI, 10 weeks, outpatient, mixed, CBT)	NMA: dropout (any reason) Narrative: none
Grabowski 1995 [87] S2; USA	228 (NR, NR, NR, NR, NR, NR); NR (NR, NR, NR, NR, NR, NR)	32.8 (6.7); 72.3% male; 64.0% Black; 27.7% White; 7.6% Hispanic; 0.7% Asian	No co‐dependencies; No comorbidities	NR (NR)	(1) Fluoxetine + 5 visits/week (40 mg 1×/day, SSRIs + OSA/ANTID) (2) Fluoxetine + 2 visits/week (40 mg 1×/day, SSRIs + OSA/ANTID) (3) Fluoxetine + 5 visits/week (20 mg 1×/day, SSRIs+OSA/ANTID) (4) Fluoxetine + 2 visits/week (20 mg 1×/day, SSRIs + OSA/ANTID (5) Placebo + 5 visits/week (6) Placebo + 2 visits/week (AI, 14 weeks, outpatient, mixed, CBT)	NMA: dropout (any reason) Narrative: craving
Grabowski 1997 [88]; USA	49 (25, 24); 49 (25, 24)	34.3 (NR); 78% male; 57% Black; 34.5% White; 8.5% Hispanic	No co‐dependencies; No comorbidities	Mixed (79% crack; 13% cocaine; 6% powder; 2% freebase)	(1) Methylphenidate (20 mg 2×/day, STIM/ARA) (2) Placebo (AI, 11 weeks, outpatient, mixed, CBT)	NMA: dropout (any reason) Narrative: craving
Grabowski 2000 [31]; USA	125 (NR, NR, NR, NR); NR (12, NR, NR, NR)	34.8 (7); 74% male; 49% Black; 43% White; 8% Hispanic	No co‐dependencies; No comorbidities	Mixed (70.6% crack or freebase; 26.1% powder; 17% speed; 1.7% other)	(1) Risperidone (8 mg 1×/day, SGAs/ABA) (2) Risperidone (4 mg 1×/day, SGAs/ABA) (3) Risperidone (2 mg 1×/day, SGAs/ABA) (4) Placebo (AI, 14 weeks, outpatient, unsupervised, CBT)	NMA: none Narrative: extent of use (proportion tests/days; EoT)
Grabowski 2001 [89]; USA	128 (NR, NR, NR); NR (NR, NR, NR)	36 (6.4); 79% male; Black 58%; White 31%; Hispanic: 9%	No co‐dependencies; No comorbidities	Mixed (22% crack or freebase; 5.7% powder or snow; 0.8% other; 69.9% a combination of crack, powder and speed; 1.6% all forms mentioned above)	(1) Dexamfetamine (60 mg 2×/day, STIM/ARA) (2) Dextroamphetamine (30 mg 2×/day, STIM/ARA) (3) Placebo (AI, 14.5 weeks (101 days), outpatient, unsupervised, CBT)	NMA: adherence Narrative: none
Grabowski 2004 [90] S1; USA	94 (28, 26, 40); NR (NR, NR, NR)	36.7 (7.3); 67% male; 10.6% Black; 75.5% White; 13.8% Hispanic	Heroin dependent; No comorbidities	Mixed (46.8% crack or freebase; 31.9% powder or snow; 21.3% ‘speedball’)	(1) Dexamfetamine (60 mg 2×/day, STIM/ARA) (2) Dexamfetamine (30 mg 2×/day, STIM/ARA) (3) Placebo (AI, 27 weeks, outpatient, mixed, CBT + methadone)	NMA: dropout (any reason); adherence Narrative: none
Grabowski 2004 [90] S2; USA	96 (31, 32, 33); (NR, NR, NR)	36.9 (7.5); 59.4% male; 10.4% Black; 79.2% White; 10.4% Hispanic	Heroin dependent; No comorbidities	Mixed (42.7% powder; 39.6% crack or freebase; 17.7% ‘speedball’)	(1) Risperidone (4 mg 1×/day, SGAs/ABA) (2) Risperidone (2 mg 1×/day, SGAs/ABA) (3) Placebo (AI, 34 weeks, outpatient, mixed, CBT + methadone)	NMA: dropout (any reason); adherence Narrative: none
Halikas 1997 [91]; USA	183 (58, 63, 62); 150 (49, 50, 51)	32.5 (5.6); 71% male; White 66.1% (*n* = 121); Black 27.9% (*n* = 51); Hispanic 2.2% (*n* = 4); Native American 2.2% (*n* = 4); Other 1.6% (*n* = 3); Not ascertained 0%	No co‐dependencies; No comorbidities	NR (NR)	(1) Carbamazepine (200 mg 2×/day, ACV + MS/ACV + GABA) (2) Carbamazepine (400 mg 2×/day, ACV + MS/ACV + GABA) (3) Placebo (AI, 12 weeks, outpatient, unsupervised, other PSI)	NMA: dropout (any reason); craving Narrative: extent of use (BE level; EoT)
Hall 1994 [92] (Tunis 1994 [93]); USA	94 (23, 22, 28, 21); NR (NR, NR, NR, NR)	38.1 (6.56); 100% male; African American 85% (*n* = 80); Asian 2% (*n* = 2); Caucasian 4% (*n* = 4); Hispanic 5% (*n* = 5); Other 3% (*n* = 3)	No co‐dependencies; No comorbidities	Mixed (all crack or other freebase)	(1) Desipramine + Enhanced Continuity Treatment (100 mg 2×/day, MA‐AD/ANTID) (2) Desipramine (100 mg 2×/day, MA‐AD/ANTID) (3) Placebo + Enhanced Continuity Treatment (4) Placebo (AI, 12 weeks, inpatient, mixed, other PSI)	NMA: point abstinence (EoT); point abstinence (follow‐up) Narrative: craving
Hamilton 2009 [94]; USA	48 (23, 25); NR (NR, NR)	45.8 (6.8, 33–60); 100% male; 85.4% Black; 14.6% White	No co‐dependencies; No comorbidities	NR (73.9% smoking; 10.9% nasal; 6.5% intravenous; 6.5% non‐intravenous injection; 2.2% oral)	(1) Olanzapine (5 mg 1×/day, SGAs/ABA) (2) Placebo (AI, 16 weeks, outpatient, unsupervised, other PSI)	NMA: continuous abstinence (treatment); point abstinence (EoT); point abstinence (follow‐up); adherence Narrative: craving
Handelsman 1995 [95]; USA	67 (23, 19,25); NR (NR, NR,NR)	36.61 (6.3); 100% male; NR^p^	Methadone‐maintained patients; No comorbidities	NR (NR)	(1) Amantadine (200 mg 2×/day, NMDAR‐ANT/GLUT) (2) Amantadine (200 mg 1×/day, NMDAR‐ANT/GLUT) (3) Placebo (AI, 8 weeks, outpatient, mixed, methadone)	NMA: dropout (any reason); extent of use (proportion tests/days; EoT); extent of use (quantity; EoT); craving Narrative: extent of use (frequency; EoT)
Handelsman 1997 [95]; USA	NR (NR, NR); 60 (30, 30)	38.48 (8.5); 100% male; Black 12 (24%); Hispanic 29 (58%); White 6 (12%); Other 3 (6%)^p^	Heroin dependent; No comorbidities	NR (NR)	(1) Bromocriptine (2.5 mg 2×/day, DA/ARA) (2) Placebo (AI, 5 weeks, outpatient, mixed, CBT + methadone)	NMA: dropout (any reason); extent of use (frequency; EoT); craving Narrative: adherence
Harris 2004 [32]; USA	82 (NR, NR); 29 (NR, NR)	40 (6); 90% male; *n* = 5 (22.7%) Caucasian; *n* = 13 (59.1%) African American; *n* = 3 (13.6%) Hispanic; *n* = 1 (4.5%) Pacific Islander^t^	No co‐dependencies; No comorbidities	NR (NR)	(1) Fluoxetine (40 mg 1×/day, SSRIs + OSA/ANTID) (2) Placebo (NA, 11 weeks, mixed, NR, CBT)	NMA: none Narrative: extent of use (BE level; EoT)
Hersh 1998 [96]; USA	64 (31, 33); NR (NR, NR)	35.5 (6); 92.2% male; 76.6% White	Abuse or dependence on alcohol; No comorbidities	NR (NR)	(1) Naltrexone (50 mg 1×/day, NTX/OAA) (2) Placebo (AI, 8 weeks, outpatient, unsupervised, RP)	NMA: dropout (any reason); dropout (adverse events); extent of use (frequency; EoT); extent of use (quantity; EoT); adherence Narrative: continuous abstinence (treatment); extent of use (proportion tests/days; EoT); craving
Johnson 1997 [97]; USA	65 (32, 33); NR (NR, NR)	35.14 (6.31); 84.6% male; 43.1% Caucasian; 49.2% African American; 7.7% Hispanic American	No co‐dependencies; No comorbidities	NR (NR)	(1) Ritanserin (10 mg 1×/day, SSRIs+OSA/ANTID) (2) Placebo (AI, 4 weeks, outpatient, unsupervised, other PSI)	NMA: dropout (any reason); craving; adherence Narrative: serious adverse events; extent of use (BE level; EoT); extent of use (BE level; follow‐up)
Johnson 2006 [98]; USA	63 (19, 12, 16, 16); NR (NR, NR, NR, NR)	36.1 (8); 87% male; White 38% (*n* = 24); Hispanic 44% (*n* = 28); Black 16% (*n* = 10); Other 2% (*n* = 1)	No co‐dependencies; No comorbidities	NR (44% intranasal; 40% smoking; 16% intravenous)	(1) Ondansetron (4 mg 2×/day, Ondansetron/ANTID) (2) Ondansetron (1 mg 2×/day, Ondansetron/ANTID) (3) Ondansetron (0.25 mg 2×/day, Ondansetron/ANTID) (4) Placebo (AI, 8 weeks, outpatient, unsupervised, CBT)	NMA: point abstinence (EoT); adherence Narrative: serious adverse events; mortality; extent of use (proportion tests/days; EoT)
Johnson 2013 [99] (Blevins 2019 [100]); USA	142 (71, 71); 129 (63, 66)	43.7 (8.1); 72.5% male; 41 (28.9%) White; 100 (70.4%) Black; 1 (0.7%) Asian	No co‐dependencies; No comorbidities	NR (86.6% smoking; 14.8% nasal; 1.4% oral)	(1) Topiramate (25–150 mg 2×/day, ACV + MS/ACV + GABA) (2) Placebo (AI, 12 weeks, outpatient, unsupervised, CBT)	NMA: dropout (any reason); extent of use (proportion tests/days; EoT); adherence Narrative: serious adverse events; mortality; continuous abstinence (treatment); craving
Johnson 2020 [101]; USA	52 (30, 22); NR (NR, NR)	51.7 (5.1); 69% male; 33/39 Black; 6/39 White^p^	No co‐dependencies; No comorbidities	Mixed (‘primarily crack’)	(1) d‐Cycloserine (50 mg 3×/week, NNM‐GluM/GLUT) (2) Placebo (AI, 3 weeks, outpatient, supervised, CM)	NMA: dropout (any reason); continuous abstinence (follow‐up); point abstinence (follow‐up); extent of use (proportion tests/days; EoT) Narrative: LDCA; extent of use (proportion tests/days; follow‐up); extent of use (frequency; EoT); craving
Jones 2004 [102]; USA	199 (45, 56, 58, 40); NR (NR, NR, NR, NR)	36.13 (6.9); 55.7% male; White *n* = 71 (38.8%)^m^	No co‐dependencies; No comorbidities	NR (10.9% intravenous)	(1) Tryptophan + CM‐abstinence (4 g 2×/day, SSRIs + OSA/ANTID) (2) Tryptophan + CM‐attendance (4 g 2×/day, SSRIs + OSA/ANTID) (3) Placebo + CM‐abstinence (4) Placebo + CM = attendance (RP, 17 weeks, mixed, mixed, CBT + CM)	NMA: dropout (any reason); LDCA; extent of use (proportion tests/days; EoT); extent of use (frequency; EoT) Narrative: none
Junior 2024 [103]; Brazil	128 (67, 61); NR (NR, NR)	35.4 (9.78, 18–65); 100% male; NR	No co‐dependencies; No comorbidities	NR (NR)	(1) Biperiden (2 mg 3×/day, mAChR‐Ant/ONA) (2) Placebo (RP, 90 days, mixed, NR, other PSI)	NMA: dropout (any reason) Narrative: extent of use (frequency; EoT); extent of use (frequency; follow‐up); extent of use (quantity; EoT); extent of use (quantity; follow‐up); craving
Kablinger 2012 [104]; USA	45 (15, 15, 15); NR (NR, NR, NR)	40.6 (7.54); 65.4% male; African American 69.2% (*n* = 18); White 30.8% (*n* = 8)^p^	No co‐dependencies; No comorbidities	NR (NR)	(1) Metyrapone + oxazepam (750/10 mg 2×/day, MTP + OXZ/COMBO) (2) Metyrapone + oxazepam (250/10 mg 2×/day, MTP + OXZ/COMBO) (3) Placebo (AI, 6 weeks, outpatient, unsupervised, no adjuvant therapy)	NMA: dropout (any reason); dropout (adverse events); craving Narrative: extent of use (BE level; EoT)
Kahn 2009 [105]; USA	160 (80, 80); NR (NR, NR)	42.65 (8.08); 79.4% male; White 25.6% (*n* = 41); Black 58.8% (*n* = 94); Hispanic 13.8% (*n* = 22); Asian/Pacific 1.3% (*n* = 2); American Indian 0.6% (*n* = 1)	No co‐dependencies; No comorbidities	NR (80.6% smoked; 13.1% nasal; 6.3% other)	(1) Baclofen (20 mg 3×/day, Baclofen/ACV + GABA) (2) Placebo (AI, 8 weeks, outpatient, unsupervised, CBT)	NMA: dropout (any reason); serious adverse events; LDCA; extent of use (proportion tests/days; EoT) Narrative: extent of use (BE level; EoT); craving
Kampman 1996 [106] (Kampman 2000 [107]); USA	61 (30, 31); NR (NR, NR)	35.05 (6.36); 82% male; African American 68.9% (*n* = 42); White 31.1% (*n* = 19)	No co‐dependencies; No comorbidities	NR (72.1% freebase; 26.2% inhaled; 1.6% intravenous)	(1) Amantadine (100 mg 3×/day, NMDAR‐ANT/GLUT) (2) Placebo (AI, 4 weeks, outpatient, mixed, RP)	NMA: dropout (any reason); continuous abstinence (treatment); extent of use (BE level; EoT); extent of use (frequency; EoT); extent of use (frequency; follow‐up); extent of use (quantity; EoT); extent of use (quantity; follow‐up) Narrative: craving
Kampman 2001 [108]; USA	108 (56, 52); NR (NR, NR)	37.68 (6.38); 81.9% male; 76.3% African American; 23.7% White	No co‐dependencies; No comorbidities	Mixed (85.5% smoked; 10.9% intranasal; 3.6% intravenous)	(1) Placebo (0 mg 3×/day, placebo/PLACEBO) (2) Propranolol (33 mg 3×/day, ADR‐ANT/ABA) (AI, 8 weeks, outpatient, unsupervised, CBT)	NMA: dropout (any reason); adherence Narrative: serious adverse events; extent of use (BE level; EoT); craving
Kampman 2003a [109]; USA	30 (15, 15); NR (NR, NR)	41.29 (5.97); 73.3% male; African American 93.3% (*n* = 28); Caucasian 3.3% (*n* = 1); Native American 3.3% (*n* = 1)	No co‐dependencies; No comorbidities	NR (86.7% smoked; 10% intranasal; 13.3% intravenous)	(1) Olanzapine (10 mg 1×/day, SGAs/ABA) (2) Placebo (AI, 11 weeks, outpatient, unsupervised, CBT)	NMA: dropout (any reason); extent of use (frequency; EoT); extent of use (quantity; EoT); craving; adherence Narrative: serious adverse events
Kampman 2003b [110]; USA	44 (18, 14); NR (NR, NR)	41.36 (6.02); 79.5% male; 93% African American; 7% White	No co‐dependencies; No comorbidities	NR (93% smoked; 7% intranasal)	(1) Piracetam (2.4 g 2×/day, nootropics/ONA) (2) Placebo (RP, 8 weeks, outpatient, unsupervised, other PSI)	NMA: dropout (any reason); serious adverse events; extent of use (BE level; EoT) Narrative: craving
Kampman 2004 [111]; USA	40 (20, 20); NR (NR, NR)	40 (6.66); 97.5% male; 90% African American; 10% Caucasian	No co‐dependencies; No comorbidities	Mixed (87.5% smoked crack; 12.5% intranasal)	(1) Topiramate (200 mg 1×/day, ACV + MS/ACV + GABA) (2) Placebo (RP, 13 weeks, outpatient, unsupervised, CBT)	NMA: dropout (any reason); point abstinence (EoT) Narrative: serious adverse events; craving
Kampman 2006 [112] (Fairbairn 2008 [113]); USA	199 (50, 50, 50, 49); NR (NR, NR, NR, NR)	40.55 (6.97); 65.3% male; African American 80.4% (*n* = 160); Caucasian 17.1% (*n* = 34); Hispanic 2.5% (*n* = 5)	No co‐dependencies; No comorbidities	NR (86.4% smoked; 11.1% intranasal; 3.5% injected)	(1) Amantadine (100 mg 3×/day, NMDAR‐ANT/GLUT) (2) Amantadine + propranolol (100/33.3 mg 3×/day, NMDAR‐ANT + ADR‐ANT/COMBO) (3) Propranolol (33.3 mg 3×/day, ADR‐ANT/ABA) (4) Placebo (AI, 8 weeks, outpatient, unsupervised, CBT)	NMA: dropout (any reason); point abstinence (EoT); adherence Narrative: serious adverse events; craving
Kampman 2011 [114]; USA	60 (34, 26); NR (NR, NR)	45.65 (8.28); 75% male; *n* = 51 (85%) African American; *n* = 9 (15%) Caucasian	No co‐dependencies; No comorbidities	Mixed (93% crack/smokers; 7% powder/intranasal)	(1) Acamprosate (666mg 3x/day, NMDAR‐ANT/GLUT) (2) Placebo (AI, 8 weeks, outpatient, unsupervised, CBT)	NMA: dropout (any reason); extent of use (proportion tests/days; EoT); adherence Narrative: extent of use (frequency; EoT); craving
Kampman 2013 [115]; USA	170 (83, 87); 170 (83, 87)	43.98 (7.57); 79.4% male; African American 82.9% (*n* = 141); Caucasian 17.1% (*n* = 29)	Alcohol dependent; No comorbidities	NR (77.1% smoked; 21.2% intranasal; 1.2% oral; 0.6% injected)	(1) Topiramate (150 mg 2×/day, ACV + MS/ACV + GABA) (2) Placebo (RP, 12 weeks, outpatient, unsupervised, CBT)	NMA: dropout (any reason); continuous abstinence (treatment); adherence Narrative: craving
Kampman 2015 [116]; USA	94 (47, 47); 94 (47, 47)	46.5 (8.74); 81% male; African American 74.5% (*n* = 70); Caucasian 25.5% (*n* = 24)	No co‐dependencies; No comorbidities	NR (84.0% smoked; 13.8% intranasal; 2.1% injected)	(1) Modafinil (100 mg 3×/day, STIM/ARA) (2) Placebo (AI, 8 weeks, outpatient, unsupervised, CBT + CM)	NMA: dropout (any reason); dropout (adverse events); continuous abstinence (treatment); adherence Narrative: extent of use (frequency; EoT); extent of use (quantity; EoT); craving
Karila 2016 [117]; France	29 (NR, NR); 27 (14, 13)	37.64 (6.13, 26–52); 100% male; NR^m^	No co‐dependencies; No comorbidities	NR (NR)	(1) Modafinil (400 mg 1×/day, STIM/ARA) (2) Placebo [RP, 12.5 weeks (87 days), mixed, NR, NR]	NMA: dropout (any reason); continuous abstinence (treatment) Narrative: none
Kennedy 2012 [118]; USA	46 (15, 14); 28 (15, 13)	48.3 (5.21); 100% male; African American 100%	No co‐dependencies; No comorbidities	NR (NR)	(1) d‐Cycloserine (50 mg 1×/week, NNM‐GluM/GLUT) (2) Placebo (AI, 4 weeks, outpatient, unsupervised, CBT)	NMA: dropout (any reason); continuous abstinence (treatment) Narrative: none
Kolar 1992 [119]; USA	NR (NR, NR, NR); 24 (NR, NR, NR)	34.8 (SE = 0.9); 85% male; White: 32%; Black: 68%^m^	Recruited from a methadone clinic; No comorbidities	NR (96% intravenous; 4% freebase)	(1) Amantadine (200 mg 1×/day, NMDAR‐ANT/GLUT) (2) Desipramine (200 mg 1×/day, MA‐AD/ANTID) (3) Placebo (AI, 12 weeks, NR, supervised, other PSI)	NMA: continuous abstinence (treatment); continuous abstinence (follow‐up); extent of use (quantity; EoT); craving Narrative: dropout (adverse events)
Kosten 1992 [120] (Leal 1994 [121]; Ziedonis 1991a [122]; Ziedonis 1991b [123]); USA	94 (33, 30, 31); NR (NR, NR, NR)	32 (SE: 0.5); 52% male; 82% White	Opiate dependent; No comorbidities	NR (57.4% only intravenous use)	(1) Amantadine (300 mg 1×/day, NMDAR‐ANT/GLUT) (2) Desipramine (150 mg 1×/day, MA‐AD/ANTID) (3) Placebo (AI, 12 weeks, outpatient, supervised, RP + methadone)	NMA: dropout (any reason); dropout (adverse events); continuous abstinence (treatment); extent of use (quantity; EoT) Narrative: craving
Kosten 2002 [124]; USA	40 (20, 20); 39 (20, 19)	39.35 (7.43); 71.8% male; 53.8% Caucasian (*n* = 21); 15.4% African American (*n* = 6); 30.8% Other (*n* = 12)^m^	Opioid dependent; No comorbidities	NR (NR)	(1) Ketoconazole (600–900 mg 1×/day, ketoconazole/ONA) (2) Placebo (AI, 11 weeks, NR, supervised, other PSI + methadone)	NMA: dropout (any reason) Narrative: none
Kosten 2003 [125] (Gonzalez 2003b [126]; Kosten 2003b [127]; Sofuoglu 2003 [128]); USA	160 (40, 40, 40, 40); NR (NR, NR, NR, NR)	36.93 (7.93); 65.6% male; White 55.6% (*n* = 89); African American 36.3% (*n* = 58); Hispanic 6.9% (*n* = 11); Native American: 1.3% (*n* = 2)	Opiate dependent; No comorbidities	NR (NR)	(1) Desipramine + CM (150 mg 1×/day, MA‐AD/ANTID) (2) Desipramine (150 mg 1×/day, MA‐AD/ANTID) (3) Placebo + CM (4) Placebo (AI, 11 weeks, outpatient, NR, RP + CM + buprenorphine)	NMA: LDCA Narrative: serious adverse events
Kosten 2013 [129] (Kampangkaew 2019 [130]; Nielsen 2012 [131]; Shorter 2013 [132]; Spellicy 2012 [133]); USA	74 (34, 40); NR (NR, NR)	38.75 (10); 70.3% male; African American 13.5% (*n* = 10); Hispanic 10.8% (*n* = 8); Caucasian 75.7% (*n* = 56)	Opioid dependent; No comorbidities	NR (NR)	(1) Disulfiram (250 mg 1×/day, disulfiram/ABA) (2) Placebo (AI, 10 weeks, outpatient, mixed, CBT + methadone)	NMA: dropout (any reason); dropout (adverse events); extent of use (proportion tests/days; EoT) Narrative: none
Kosten 2014 [134]; USA	300 (152, 148); NR (NR, NR)	46.08 (6.55); 76% male; Hispanic 7.7% (*n* = 23); Caucasian 22.0% (*n* = 66); African American 72.3% (*n* = 217); Asian 1.0% (*n* = 3); American Indian/Alaskan native 1.0% (*n* = 3); Unknown 3.7% (*n* = 11)	No co‐dependencies; No comorbidities	NR (NR)	(1) TA‐CD vaccine (400 μg 1× at weeks 1, 3, 5, 9 and 13, Coc‐PK/ABA) (2) Placebo (AI, 16 weeks, outpatient, supervised, CBT)	NMA: dropout (any reason); serious adverse events; continuous abstinence (treatment) Narrative: none
Kranzler 1995 [135]; USA	40 (20, 20); NR (NR, NR)	34.35 (5.45); 100% male; White 65% (*n* = 26); Black 32.5% (*n* = 13); Hispanic: 5% (*n* = 1)	No co‐dependencies; No comorbidities	NR (75% smoked; 25% intranasal)	(1) Carbamazepine (200 mg 3×/day, ACV + MS/ACV + GABA) (2) Placebo (AI, 12 weeks, NR, NR, RP)	NMA: dropout (any reason); dropout (adverse events); extent of use (frequency; EoT); extent of use (frequency; follow‐up); extent of use (quantity; EoT); extent of use (quantity; follow‐up); adherence Narrative: extent of use (BE level; EoT); extent of use (BE level; follow‐up)
LaRowe 2013 [136]; USA	126 (43, 41, 42); 111 (40, 33, 38)	43.2 (9.2); 75% male; 48 Caucasians, 61 African Americans, and 2 Hispanics^m^	No co‐dependencies; No comorbidities	NR (72% reported primarily smoking cocaine)	(1) *N*‐Acetylcysteine (600 mg 2×/day, NNM‐GluM/GLUT) (2) *N*‐Acetylcysteine (1200 mg 2×/day, NNM‐GluM/GLUT) (3) Placebo (AI, 8 weeks, outpatient, unsupervised, CBT)	NMA: dropout (any reason); extent of use (BE level; EoT); adherence Narrative: serious adverse events; extent of use (proportion tests/days; EoT)
Levin 1999 [137]; USA	14 (9, 5); NR (NR, NR)	39.3 (6.35); 78.6% male; African American 6/14 42.9%; Caucasian 2/14 14.3%; Hispanic 6/14 42.9%	No co‐dependencies; No comorbidities	NR (50% intranasal; 50% freebase/intravenous)	(1) Risperidone (6 mg 1×/day, SGAs/ABA) (2) Placebo (AI, 12 weeks, outpatient, unsupervised, RP)	NMA: dropout (any reason) Narrative: dropout (adverse events); serious adverse events; extent of use (frequency; EoT)
Levin 2007 [138] (Aharonovich 2006 [139]); USA	106 (53, 53); NR (NR, NR)	37 (6.49); 83% male; African American *n* = 21 (19.81%); Hispanic *n* = 15 (14.15%); Caucasian *n* = 64 (60.38%); Other *n* = 6 (5.66%)	NR; All had ADHD	NR (60.4% intranasal; 34.0% smoking; 4.7% other)	(1) Methylphenidate (sustained‐release formulation) (30 mg 2×/day, STIM/ARA) (2) Placebo (AI, 13 weeks, outpatient, unsupervised, CBT)	NMA: dropout (any reason); dropout (adverse events); continuous abstinence (treatment); extent of use (proportion tests/days; EoT); adherence Narrative: serious adverse events; craving
Levin 2015 [140] (Blevins 2020 [141]; Levin 2018 [142]; Notzon 2016 [143]); USA	126 (43, 40, 43); 126 (43, 40, 43)	40.43 (8.14); 84.1% male; Hispanic 17.5% (*n* = 22); Black 17.5% (*n* = 22); White 57.1% (*n* = 72); Asian, Native American, and Other 7.9% (*n* = 10)	No co‐dependencies; DSM‐IV‐TR diagnosis for current adult ADHD	NR (NR)	(1) MAS‐ER extended release + topiramate (80 mg 1×/day, STIM + ACV/COMBO) (2) MAS‐ER extended release + topiramate (60 mg 1×/day, STIM + ACV/COMBO) (3) Placebo (AI, 13 weeks, outpatient, unsupervised, CBT)	NMA: dropout (any reason); serious adverse events; continuous abstinence (treatment); point abstinence (EoT) Narrative: adherence
Levin 2020 [144]; USA	127 (64, 63); 127 (64, 63)	46.2 (8.4); 75.6% male; NR Other 8/127 (6.6%);	No co‐dependencies; No comorbidities	NR (NR)	(1) MAS‐ER + topiramate (60/100 mg 1× and 2×/day, STIM + ACV/COMBO) (2) Placebo (AI, 12 weeks, outpatient, unsupervised, other PSI)	NMA: dropout (any reason); serious adverse events; continuous abstinence (treatment); craving; adherence Narrative: none
Licata 2011 [145] (Bracken 2011 [146]); USA	29 (15, 14); NR (NR, NR)	38.69 (4.82); 62.1% male; Hispanic 3.4% (*n* = 1); American Indian 3.4% (*n* = 1); Cape Verdian 3.4% (*n* = 1); African American 82.8% (*n* = 24); White 6.9% (*n* = 2)	No co‐dependencies; No comorbidities	NR (NR)	(1) Citicoline (500 mg 2×/day, nootropics/ONA) (2) Placebo (AI, 8 weeks, outpatient, unsupervised, other PSI)	NMA: dropout (any reason); extent of use (frequency; EoT); extent of use (frequency; follow‐up); craving Narrative: serious adverse events
Ling 2016 [147] (Nielsen 2022 [148]); USA	302 (100, 100, 102); 299 (98, 99, 102)	46.4 (8.66); 78.5% male; American Indian or Alaska Native 4/302 (1.3%); Asian 1/302 (0.33%); African American 191/302 (63.2%); White 78/302 (25.8%); Other 12/302 (4.0%); Multiracial 16/302 (5.3%); Hispanic 31/302 (10.3%)	Past‐year opioid dependence or abuse, or past‐year opioid use with a lifetime history of opioid dependence; No comorbidities	NR (NR)	(1) Buprenorphine + naloxone (4 mg 1×/day, OAT/OAA) (2) Buprenorphine + naloxone (16 mg 1×/day, OAT/OAA) (3) Placebo (AI, 8 weeks, outpatient, mixed, CBT)	NMA: dropout (any reason); serious adverse events; mortality; continuous abstinence (treatment); extent of use (proportion tests/days; EoT); adherence Narrative: none
Loebl 2008 [149]; USA	31 (16, 15); NR (NR, NR)	43.3 (6.1); 100% male; African American 19 (61%); White 12 (39%)	No co‐dependencies; No comorbidities	NR (NR)	(1) Risperidone [25 mg 1×/day (week 1), then biweekly, SGAs/ABA] (2) Placebo (AI, 12 weeks, NR, NR, other PSI)	NMA: dropout (any reason); dropout (adverse events); extent of use (proportion tests/days; EoT); extent of use (frequency; EoT) Narrative: serious adverse events; craving
Lynch 2022 [150]; USA	156 (80, 76); 156 (80, 76)	51 (7.6); 78.2% male; Black or African American 87.2% (*n* = 136); White 12.8% (*n* = 20)	No co‐dependencies; No comorbidities	NR (82.1% smoked; 17.9% intranasal)	(1) Varenicline (0 mg 2×/day, varenicline/ONA) (2) Placebo (AI, 12 weeks, outpatient, unsupervised, CBT)	NMA: dropout (any reason); dropout (adverse events); serious adverse events; continuous abstinence (treatment); point abstinence (EoT); adherence Narrative: mortality
Malcolm 2000 [151]; (Malcolm 2001 [152]); USA	464 (155, 156, 153); 358 (117, 110, 131)	NR (NR); 78.8% male; 52.2% Black; 46.4% White; 1.11% Other; 0.28% missing^m^	191 met criteria for alcohol dependence; No comorbidities	Crack (NR)	(1) Pergolide (0.05 mg 2×/day, DA/ARA) (2) Pergolide (0.25 mg 2×/day, DA/ARA) (3) Placebo (AI, 12 weeks, outpatient, mixed, NR)	NMA: dropout (any reason); point abstinence (follow‐up); adherence Narrative: dropout (adverse events); extent of use (proportion tests/days; EoT); craving
Malcolm 2005 [153] (Malcolm 2002 [154]; Turner 2009 [155]); USA	195 (NR, NR); 116 (60, 56)	35.8 (7.3); 79% male; The subject population was 56% African American, 44% Caucasian	No co‐dependencies; No comorbidities	NR (NR)	(1) Amlodipine (7.5–10 mg 1×/day, Amlodipine/ONA) (2) Placebo (AI, 14 weeks, outpatient, mixed, CBT)	NMA: dropout (any reason); point abstinence (EoT); extent of use (BE level; EoT); adherence Narrative: none
Mancino 2014 [156]; USA	107 (34, 35, 38); 103 (30, 35, 38)	39.5 (7.3); 77% male; 71.6% African American^m^	No co‐dependencies; Depression, with score >15 on HAM‐D	NR (NR)	(1) Gabapentin + sertraline (600/100 mg 2×/day, Gaba + SSRI/COMBO) (2) Sertraline (200 mg 2×/day, SSRIs + OSA/ANTID) (3) Placebo (RP, 12 weeks, mixed, mixed, CBT)	NMA: dropout (any reason); serious adverse events; continuous abstinence (treatment); adherence Narrative: none
Margolin 1995 [157]; USA	150 (NR, NR); 149 (74, 75)	37.2 (6.9); 62% male; 67 (45%) White; 64 (43%) African American; 18 (12%) Hispanic and Other ethnic groups^m^	Methadone‐maintained patients; No comorbidities	NR (NR)	(1) Bupropion (100 mg 3×/day, NDRIs/ARA) (2) Placebo (AI, 12 weeks, NR, NR, other PSI)	NMA: dropout (any reason); extent of use (proportion tests/days; EoT); extent of use (frequency; EoT); extent of use (quantity; EoT); craving Narrative: none
Margolin 1997 [158]; USA	17 (7, 6, 4); 17 (7, 6, 4)	36 (5.1); 53% male; 65% White; 24% African American; 11% Hispanic	Enrolled in inner‐city methadone maintenance programme; No comorbidities	NR (65% smoking; 29% intravenously; 6% intranasally)	(1) Mazindol (8 mg 1×/day, STIM/ARA) (2) Mazindol (1 mg 1×/day, STIM/ARA) (3) Placebo (AI, 12 weeks, outpatient, NR, methadone)	NMA: dropout (any reason); point abstinence (EoT); craving Narrative: none
Mariani 2012 [159]; USA	81 (39, 42); NR (NR, NR)	41.97 (8.53); 86.4% male; Hispanic 30.9% (*n* = 25); Black 36.5% (*n* = 30); White 30.9% (*n* = 25); Asian 1.2% (*n* = 1)	No co‐dependencies; No comorbidities	NR (NR)	(1) MAS‐ER + topiramate (30/150 mg 2×/day, STIM + ACV/COMBO) (2) Placebo (AI, 12 weeks, outpatient, unsupervised, CM + other PSI)	NMA: dropout (any reason); dropout (adverse events); serious adverse events; continuous abstinence (treatment) Narrative: adherence
Martell 2009 [160] (Kosten 2013b [161]; Nielsen 2013 [162]); USA	115 (58, 57); 114 (57, 57)	35.9 (9.07); 66.7% male; White 86.8% (*n* = 99); African American 8.8% (*n* = 10); Mixed 4.4% (*n* = 5)^m^	Opioid dependent; No comorbidities	NR (76.5% smoked; 62.3% intranasal; 29.8% intravenous)^u^	(1) TA‐CD vaccine (360 μg 1× at weeks 0, 2, 4, 8 and 12, Coc‐PK/ABA) (2) Placebo (AI, 16 weeks, outpatient, supervised, CBT + methadone)	NMA: dropout (any reason); dropout (adverse events); serious adverse events Narrative: extent of use (proportion tests/days; EoT)
McCann 2024 [163]; USA	242 (118, 124); NR (NR, NR)	50.2 (9.92); 71.9% male; American Indian or Alaska native *n* = 1 (0.4%); Asian *n* = 1 (0.4%); Black or African American *n* = 160 (66.1%); White *n* = 65 (26.9%); More than one *n* = 13 (5.4%); Unknown or NR *n* = 2 (0.8%)	No co‐dependencies; No comorbidities	NR (74.4% smoking; 22.3% nasal; 0.8% oral; 0.4% intravenous)	(1) Lorcaserin (10 mg 2×/day, lorcaserin/ANTID) (2) Placebo (AI, 12 weeks, outpatient, unsupervised, CBT)	NMA: dropout (any reason); serious adverse events; continuous abstinence (treatment); craving Narrative: mortality
McDowell 2005 [164]; USA	111 (55, 56); NR (NR, NR)	35.89 (6.94); 75% male; 27/111 (24.3%) Hispanic; 34/111 (30.6%) African American; 49/111 (44.1%) Caucasian; 1/111 (0.09%) Other	No co‐dependencies; Major depression or dysthymia	Mixed (57% intranasal cocaine; remainder using freebase/crack or intravenous cocaine)	(1) Desipramine (up to 300 mg 1×/day, MA‐AD/ANTID) (2) Placebo (AI, 12 weeks, outpatient, unsupervised, RP)	NMA: dropout (any reason); dropout (adverse events); serious adverse events; continuous abstinence (treatment); extent of use (frequency; EoT) Narrative: none
Moeller 2001 [165]; USA	41 (NR, NR); 35 (17, 18)	39.53 (6.98); NR; NR	No co‐dependencies; No comorbidities	NR (NR)	(1) Buspirone (15 mg 3×/day, buspirone/ANTID) (2) Placebo (AI, 12 weeks, outpatient, unsupervised, RP)	NMA: dropout (any reason); craving Narrative: extent of use (BE level; EoT); extent of use (proportion tests/days; EoT)
Moeller 2007 [33] (Green 2009 [166]; Schmitz 2009b [167]); USA	76 (36, 40); NR (NR, NR)	39.02 (7.33); 82.9% male; 67.1 African American; 23.7% non‐Hispanic Caucasian; 7.9% Hispanic Caucasian; 1.3% unknown	No co‐dependencies; No comorbidities	NR (NR)	(1) Citalopram (20 mg 1×/day, SSRIs + OSA/ANTID) (2) Placebo (AI, 12 weeks, outpatient, unsupervised, CBT + CM)	NMA: none Narrative: dropout (any reason); dropout (adverse events); extent of use (proportion tests/days; EoT)*
Mongeau‐Perusse 2021 [168] (Hebert 2024 [169]; Mongeau‐Perusse 2022 [170]; Morissette 2021 [171]; Rizkallah 2022 [172]); Canada	78 (40, 38); 75 (39, 36)	45.9 (11.2); 82.1% male; White 67/78 (85.9%); Other 11/78 (14.1%)	No co‐dependencies; No comorbidities	Mixed (55.1% smoking; 30.8% nasal; 12.8% intravenous; 1.3% non‐intravenous injection)	(1) Cannabidiol (800 mg 1×/day, CBD/ONA) (2) Placebo (RP, 92 days, mixed, mixed, RP)	NMA: dropout (any reason); serious adverse events; extent of use (proportion tests/days; EoT) Narrative: none
Montoya 1995 [173]; USA	72 (NR, NR); 62 (28, 34)	33.2 (5.4); 79% male; Black 67.7% (*n* = 42); White 30.7% (*n* = 19); Native American 1.6% (*n* = 1)^m^	No co‐dependencies; No comorbidities	NR (NR)	(1) Carbamazepine (800 mg 1×/day, ACV + MS/ACV + GABA) (2) Placebo (AI, 7 weeks, outpatient, mixed, CBT)	NMA: dropout (any reason) Narrative: extent of use (quantity; EoT); craving
Montoya 2004 [34] (Montoya 2005 [174]); USA	200 (50, 50, 50, 50); 46 (44, 43, 46, 46)	34.1 (6.17); 66.5% male; 74.9% Black	Opiate dependent; No comorbidities	NR (NR)	(1) Buprenorphine (8 mg 1×/day, OAT/OAA) (2) Buprenorphine (16 mg 0.5×/day, OAT/OAA) (3) Buprenorphine (16 mg 1×/day, OAT/OAA) (4) Buprenorphine (2 mg 1x/day, OAT/OAA) (AI, 10 weeks, outpatient, supervised, other PSI)	NMA: none Narrative: dropout (any reason); serious adverse events; extent of use (BE level; EoT); extent of use (BE level; follow‐up); extent of use (proportion tests/days; EoT)^w^
Mooney 2007 [175] S1; USA	67 (31, 36); 67 (31, 36)	35.2 (7.1); 67.5% male; 30 White (44.8%); 31 Black (46.3%); 5 Hispanic (7.5%); 1 missing (1.5%)	No co‐dependencies; No comorbidities	NR (74% crack)	(1) Levodopa + carbidopa (400/100 mg 2×/day, l‐DOPA+/ARA) (2) Placebo (AI, 10 weeks, outpatient, unsupervised, other PSI)	NMA: dropout (any reason); dropout (adverse events) Narrative: extent of use (BE level; EoT); extent of use (proportion tests/days; EoT); craving; adherence
Mooney 2007 [175] S2; USA	122 (43, 39, 40); 122 (43, 39, 40)	39.7 (7); 84.3% male; 27.9% White; 63.9% Black; 5.7% Hispanic; 2.5% missing	No co‐dependencies; No comorbidities	Mixed (85% crack)	(1) Levodopa + carbidopa (400/100 mg 2×/day, l‐DOPA+/ARA) (2) Levodopa + carbidopa (800/200 mg 2×/day, l‐DOPA+/ARA) (3) Placebo (AI, 10 weeks, outpatient, unsupervised, other PSI)	NMA: dropout (any reason) Narrative: dropout (adverse events); extent of use (proportion tests/days; EoT); craving; adherence
Mooney 2009 [176]; USA	82 (30, 25, 27); 82 (30, 25, 27)	36.38 (5.89); 65.9% male; White 28.0% (*n* = 23); Black 59.8% (*n* = 49); Hispanic 12.2% (*n* = 10)	No co‐dependencies; No comorbidities	NR (70.7% crack)	(1) Methamphetamine (5 mg 6×/day, STIM/ARA) (2) Sustained release (SR) methamphetamine (30 mg 1×/day, STIM/ARA) (3) Placebo (AI, 10 weeks, outpatient, mixed, CBT + CM)	NMA: dropout (any reason); dropout (adverse events); craving; adherence Narrative: serious adverse events; extent of use (proportion tests/days; EoT)
Mooney 2015 [177]; USA	43 (22, 21); 43 (22, 21)	46.1 (7.9); 81.4% male; 60.5% Black; 27.9% White; 7% More than one race; 4.7% Native Hawaiian or Other Pacific Islander	No co‐dependencies; No comorbidities	NR (NR)	(1) Lisdexamfetamine (70 mg 1×/day, STIM/ARA) (2) Placebo (AI, 14 weeks, outpatient, unsupervised, CBT)	NMA: dropout (any reason); dropout (adverse events); serious adverse events; point abstinence (EoT); craving Narrative: adherence
Morgan 2016 [178] (Canavan 2014 [179]); USA	57 (30, 27); NR (22, NR)	43 (7); 77.2% male; NR	No co‐dependencies; No comorbidities	NR (all smoked or intravenous)	(1) Modafinil (400 mg 1×/day, STIM/ARA) (2) Placebo (RP, 7 weeks, mixed, mixed, RP + CBT)	NMA: dropout (any reason); LDCA Narrative: serious adverse events; continuous abstinence (treatment)
Nanni‐Alvarado 2022 [180]; Mexico	100 (50, 50); NR (NR, NR)	27.75 (SE = 1); 100% male; NR	No co‐dependencies; No comorbidities	NR (all smoked or intravenous)	(1) Mirtazapine (30 mg 1×/day, MA‐AD/ANTID) (2) Placebo (RP, 12 weeks, mixed, mixed, NR)	NMA: dropout (any reason); craving Narrative: continuous abstinence (treatment)
NCT03953612 2024 (Milivojevic 2022 [181]); USA	58 (21, 18, 19); 55 (20, 17, 18)	47.27 (10.48); 70.9% male; Race: Black or African American 60.0% (*n* = 33); White 34.5% (*n* = 19); Unknown or NR 5.5% (*n* = 3) Ethnicity: Hispanic or Latino 9.1% (*n* = 5)	No co‐dependencies; No comorbidities	NR (NR)	(1) Pregnenolone (150 mg 2×/day, DA/ARA) (2) Pregnenolone (250 mg 2×/day, DA/ARA) (3) Placebo (AI, 8 weeks, mixed, mixed, other PSI)	NMA: dropout (any reason); serious adverse events; extent of use (frequency; EoT); extent of use (quantity; EoT)* Narrative: none
Noel Raby 2014 [182]; USA	130 (64, 66); 130 (64, 66)	37.51 (7.98); 72% male; 38% Caucasian; 26% Hispanic; 25% African American	No co‐dependencies; Current major depressive disorder or dysthymia	NR (NR)	(1) Venlafaxine (150 mg 2×/day, MA‐AD/ANTID) (2) Placebo (AI, 12 weeks, outpatient, unsupervised, RP)	NMA: dropout (any reason); dropout (adverse events); serious adverse events; continuous abstinence (treatment); extent of use (proportion tests/days; EoT); extent of use (frequency; EoT) Narrative: none
Noel Raby 2022 [183]; USA	31 (17, 14); 26 (15, 11)	50.2 (5.4); 73.1% male; African American 92.3% (*n* = 24); Hispanic/Latino 7.7% (*n* = 2)^m^	No co‐dependencies; No comorbidities	NR (NR)	(1) Oxytocin (24 IU 1×/day, neuropeptide/ONA) (2) Placebo (RP, 6 weeks, mixed, mixed, other PSI)	NMA: dropout (any reason); point abstinence (EoT); LDCA Narrative: serious adverse events; craving
Nuijten 2016 [184] (Blanken 2020 [185]); Netherlands	73 (38, 35); 73 (38, 35)	49 (6); 90.4% male; 49/73 (67%) European descent	Heroin dependent; No comorbidities	NR (one patient injected cocaine; all others smoked)	(1) Dexamfetamine (60 mg 1×/day, STIM/ARA) (2) Placebo (AI, 12 weeks, outpatient, supervised, methadone + DAM)	NMA: dropout (any reason); dropout (adverse events); serious adverse events; continuous abstinence (treatment); LDCA; extent of use (frequency; EoT) Narrative: extent of use (quantity; EoT); craving; adherence
Oliva 2022 [186]; USA	21 (11, 10); NR (NR, NR)	43 (8); 0% male; White non‐Hispanic *n* = 9 (42.9%); Black *n* = 9 (42.9%); Hispanic *n* = 3 (14.3%)	No co‐dependencies; No comorbidities	NR (52.4% smoked; 47.6% intranasal)	(1) Progesterone (200 mg 2×/day, SEX‐H/ONA) (2) Placebo (RP, 8 weeks, mixed, unsupervised, CM)	NMA: dropout (any reason); serious adverse events; continuous abstinence (treatment) Narrative: extent of use (proportion tests/days; EoT)
Oliveto 2005 [35] (Mancino 2010 [187]); USA	140 (35, 35, 35, 35); NR (NR, NR, NR, NR)	36.5 (6.25); 67.9% male; Caucasian 65% (*n* = 91); African American 27.9% (*n* = 39); Hispanic 7.1% (*n* = 10)	Opioid dependent; No comorbidities	NR (NR)	(1) LAAM + CM (110 mg 3×/week, OAT/OAA) (2) LAAM (110 mg 3×/week, OAT/OAA) (3) LAAM (33 mg 3×/week, OAT/OAA) (4) LAAM (33 mg 3×/week, OAT/OAA) (AI, 12 weeks, outpatient, NR, CM + other PSI)	NMA: none Narrative: dropout (any reason); extent of use (quantity; EoT)
Oliveto 2011 [188] (Atkinson 2013 [189]); USA	158 (40, 39, 37, 39); 152 (39, 38, 37, 38)	36.32 (9.15); 59.2% male; Caucasian *n* = 118 (77.6%); African American *n* = 16 (10.5%); Hispanic/Other *n* = 18 (11.8%)^m^	Opioid dependent; No comorbidities	NR (NR)	(1) Disulfiram (250 mg 1×/day, Disulfiram/ABA) (2) Disulfiram (125 mg 1×/day, Disulfiram/ABA) (3) Disulfiram (62.5 mg 1×/day, Disulfiram/ABA) (4) Placebo (AI, 14 weeks, outpatient, mixed, CBT + methadone)	NMA: dropout (any reason); point abstinence (EoT); extent of use (quantity; EoT) Narrative: none
Oliveto 2012 [190]; USA	94 (50, 44); 94 (50, 44)	38.62 (5.85); 61.02% male; Caucasian *n* = 16 (27.12%); African American *n* = 39 (66.10%); Hispanic *n* = 3 (5.08%); Other *n* = 1 (1.69%)^p^	No co‐dependencies; All had a score of >15 on the Hamilton Depression Scale	NR (NR)	(1) Sertraline (200 mg 1×/day, SSRIs + OSA/ANTID) (2) Placebo (RP, 12 weeks, mixed, NR, CBT + CM)	NMA: dropout (any reason); dropout (adverse events); continuous abstinence (treatment) Narrative: extent of use (frequency; EoT); extent of use (quantity; EoT)
Passos 2005 [191]; Brazil	210 (105, 105); NR (NR, NR)	30.8 (7.6); 92.4% male; 54.8% White	No co‐dependencies; No comorbidities	Powder (NR)	(1) Nefazodone (300 mg 1×/day, SSRIs + OSA/ANTID) (2) Placebo (AI, 10 weeks, outpatient, unsupervised, other PSI)	NMA: dropout (any reason); dropout (adverse events); craving; adherence Narrative: none
Petrakis 2000 [192] (Jofre‐Bonet 2004 [193]); USA	69 (NR, NR); 67 (36, 31)	NR (NR); 48% male; 73% Caucasian^m^	Opioid dependent; No comorbidities	NR [62% freebase (smoking); 27% intravenously or subcutaneously; 11% intranasal]	(1) Disulfiram (250 mg 1×/day, disulfiram/ABA) (2) Placebo (AI, 12 weeks, outpatient, supervised, other PSI + methadone)	NMA: dropout (any reason); dropout (adverse events); extent of use (frequency; EoT); extent of use (quantity; EoT) Narrative: craving
Pettinati 2008 [194]; USA	208 (53, 49, 52, 54); NR (NR, NR, NR, NR)	41.24 (6.82); 70.2% male; African American 88.9%	Alcohol dependent; No comorbidities	NR (78.9% smoked crack)	(1) Disulfiram (250 mg 1×/day, disulfiram/ABA) (2) Disulfiram + naltrexone (250/100 mg 1×/day, Dis + NTX/COMBO) (3) Naltrexone (100 mg 1×/day, NTX/OAA) (4) Placebo (AI, 11 weeks, outpatient, unsupervised, CBT)	NMA: dropout (any reason); continuous abstinence (treatment) Narrative: serious adverse events; extent of use (proportion tests/days; EoT); extent of use (frequency; EoT); extent of use (quantity; EoT); adherence
Pettinati 2014 [195]; USA	80 (39, 41); NR (NR, NR)	47.9 (6.6); 81.3% male; African American 70 (87.5%)	Alcohol dependent; No comorbidities	NR (80% smoked)	(1) Naltrexone (380 mg 2× injections, NTX/OAA) (2) Placebo (AI, 8 weeks, outpatient, supervised, CBT)	NMA: dropout (any reason); serious adverse events; continuous abstinence (treatment) Narrative: adherence
Plebani 2012 [196]; USA	37 (18, 19); NR (NR, NR)	NR (NR); 70% male; African American 78.4% (*n* = 19)	No co‐dependencies; No comorbidities	NR (NR)	(1) Varenicline (2 mg 1×/day, varenicline/ONA) (2) Placebo (AI, 8 weeks, outpatient, unsupervised, CBT + CM)	NMA: point abstinence (EoT) Narrative: extent of use (frequency; EoT); extent of use (quantity; EoT)
Reid 2005a [197]; USA	23 (10, 13); NR (NR, NR)	45 (9); 78% male; White 6 (26%); Black 17 (74%)	No co‐dependencies; No comorbidities	NR (74% smoked; 26% nasal)	(1) Celecoxib (200 mg 2×/day, celecoxib/ONA) (2) Placebo (AI, 8 weeks, outpatient, unsupervised, CBT)	NMA: dropout (any reason); dropout (adverse events); point abstinence (EoT); extent of use (BE level; EoT); extent of use (frequency; EoT) Narrative: serious adverse events; adherence
Reid 2005b [198]; USA	68 (16, 18, 18, 16); 68 (16, 18, 18, 16)	38.72 (6.14); 79.4% male; Race: White 17.5% (*n* = 11); Black 81.0% (*n* = 51); Other 1.5% (*n* = 1) Ethnicity: Hispanic 11.1% (*n* = 7); non‐Hispanic 88.9% (*n* = 56)^x^	No co‐dependencies; No comorbidities	NR (76.2% smoked; 20.6% nasal; 3.2% injected)	(1) CoQ10 + l‐cartinine (100/250 mg 2×/day, CQ10 + LC/COMBO) (2) Olanzapine (5 mg 2×/day, SGAs/ABA) (3) Valproate (750 mg 2×/day, ACV + MS/ACV + GABA) (4) Placebo (AI, 8 weeks, outpatient, unsupervised, CBT)	NMA: dropout (any reason); serious adverse events; mortality; point abstinence (EoT); extent of use (BE level; EoT); extent of use (frequency; EoT); craving Narrative: adherence
Reid 2006 [199]; USA	35 (17, 18); NR (NR, NR)	NR (NR); % male; NR	Opiate dependent; No comorbidities	NR (NR)	(1) Mecamylamine (6 mg 1×/day, mecamylamine/ONA) (2) Placebo (AI, 16 weeks, outpatient, unsupervised, CBT + methadone)	NMA: dropout (any reason); serious adverse events; extent of use (frequency; EoT); craving Narrative: dropout (adverse events); point abstinence (EoT); extent of use (BE level; EoT); adherence
Renshaw 1999 [38]; USA	14 (6, 8); 10 (4, 6)	36.74 (7.38); 78.57% male; 42.86% White; 57.14% African American	No co‐dependencies; No comorbidities	Crack (NR)	(1) Citicoline (500 mg 2×/day, nootropics/ONA) (2) Placebo (AI, 2 weeks, NR, mixed, NR)	NMA: none Narrative: none
Santos 2021 [200]; USA	22 (16, 6); 22 (16, 6)	NR (NR), IQR = 32–55; 100% male; White 41% (*n* = 9); Asian 5% (*n* = 1); Black 23% (*n* = 5); Latino 14% (*n* = 3); Other 18% (*n* = 4)	No co‐dependencies; No comorbidities	Mixed (82% powder; 36% crack)^u^	(1) Lorcaserin (20 mg 1×/day, lorcaserin/ANTID) (2) Placebo (AI, 12 weeks, outpatient, unsupervised, other PSI)	NMA: dropout (any reason); serious adverse events; point abstinence (EoT); adherence Narrative: extent of use (proportion tests/days; EoT); extent of use (frequency; EoT); craving
Schmitz 1998 [39]; USA	81 (29, 23, 29); NR (NR, NR, NR)	33.4 (6.3); 81% male; 37% Caucasian; 55% Black; 6% Hispanic; 1% American Indian	No co‐dependencies; No comorbidities	NR (80% smoked crack)	(1) Fluoxetine (40 mg 1×/day, SSRIs + OSA/ANTID) (2) Fluoxetine (20 mg 1×/day, SSRIs + OSA/ANTID) (3) Placebo (AI, 14 weeks, outpatient, mixed, CBT)	NMA: none Narrative: none
Schmitz 2001 [201]; USA	68 (34, 34); NR (NR, NR)	37.3 (5.85); 57.4% male; 52.9% White; 42.6% Black; 4.4% Hispanic	No co‐dependencies; Major depressive disorder	NR (NR)	(1) Fluoxetine (40 mg 1×/day, SSRIs + OSA/ANTID) (2) Placebo (AI, 12 weeks, outpatient, unsupervised, CBT)	NMA: dropout (any reason); adherence Narrative: dropout (adverse events)
Schmitz 2008 [202]; USA	161 (27, 31, 27, 25, 28, 23); 121 (20, 20, 20, 20, 20, 21)	41.35 (6.95); 83.23% male; White *n* = 39 (24.38%); Black *n* = 109 (68.13%); Hispanic *n* = 12 (7.5%)	No co‐dependencies; No comorbidities	NR (NR)	(1) Levodopa/carbidopa + CM (400/100 mg 2×/day, l‐DOPA+/ARA) (2) Levodopa/carbidopa + CBT (400/100 mg 2×/day, l‐DOPA+/ARA) (3) Levodopa/carbidopa + clinical management (400/100 mg 2×/day, l‐DOPA+/ARA) (4) Placebo + CM (5) Placebo + CBT (6) Placebo + clinical management (AI, 13 weeks, outpatient, unsupervised, CBT + CM)	NMA: dropout (any reason); continuous abstinence (treatment) Narrative: dropout (adverse events); craving
Schmitz 2009 [203]; USA	87 (NR, NR, NR, NR); 86 (25, 20, 14, 27)	34.41 (4.55); 87.3% male; African American 71.3%; Caucasian 13.8%	Alcohol dependent; No comorbidities	Mixed (81.2% smoked crack; 16.5% intranasal; 2.4% intravenous injection)	(1) Naltrexone + CM (100 mg 1×/day, NTX/OAA) (2) Naltrexone (100 mg 1×/day, NTX/OAA) (3) Placebo + CM (4) Placebo (AI, 12 weeks, outpatient, unsupervised, CBT + CM)	NMA: extent of use (proportion tests/days; EoT) Narrative: dropout (adverse events); serious adverse events
Schmitz 2010 [36]; USA	136 (23, 22, 23, 27, 20, 21); NR (NR, NR, NR, NR, NR, NR)	41 (7.5); 83% male; White 20.6% (*n* = 28); Black 70.6% (*n* = 96); Hispanic 8.8% (*n* = 12)	No co‐dependencies; No comorbidities	NR (83.8% smoking crack)	(1) Levodopa/carbidopa + CM‐urine (800/200 mg 1×/day, l‐DOPA+/ARA) (2) Levodopa/carbidopa + CM‐medication (800/200 mg 1×/day, l‐DOPA+/ARA) (3) Levodopa/carbidopa + CM‐attendance (800/200 mg 1×/day, l‐DOPA+/ARA) (4) Placebo + CM‐urine (5) Placebo + CM‐medication (6) Placebo + CM‐attendance (AI, 13 weeks, outpatient, unsupervised, CBT + CM)	NMA: none Narrative: adherence
Schmitz 2012 [204]; USA	73 (20, 22, 15, 16); 49 (11, 10, 12, 16)	42.7 (8); 84.9% male; White 23% (*n* = 17); Black 64% (*n* = 47); Hispanic 9.6% (*n* = 7)	No co‐dependencies; No comorbidities	NR (NR)	(1) Modafinil (133.3 mg 2×/day, STIM/ARA) (2) Dexamfetamine (20 mg 2 x/day, STIM/ARA) (3) Dexamfetamine + modafinil (10/66.6 mg 2 x/day, STIM/ARA) (4) Placebo; (AI, 16 weeks, outpatient, unsupervised, CBT)	NMA: dropout (any reason); extent of use (proportion tests/days; EoT) Narrative: dropout (adverse events); serious adverse events
Schmitz 2014 [205]; USA	81 (25, 22, 16, 18); 75 (24, 18, 15, 18)	42.16 (8.67); 82.7% male; African American 69.1% (*n* = 56); Caucasian 17.3% (*n* = 14); Hispanic 13.6% (*n* = 11)	No co‐dependencies; No comorbidities	NR (NR)	(1) Levodopa + carbidopa (sustained release) (400/100 mg 2×/day, l‐DOPA+/ARA) (2) Modafinil (200 mg 2×/day, STIM/ARA) (3) Naltrexone (25 mg 2×/day, NTX/OAA) (4) Placebo (M, 12 weeks, outpatient, mixed, motivational + CM)	NMA: dropout (any reason); serious adverse events; extent of use (proportion tests/days; EoT); adherence Narrative: none
Schmitz 2017 (206)^6^; USA	30 (15, 15); 26 (12, 14)	47.8 (7.45); 73.3% male; *n* = 19 (63.3%) Black; *n* = 7 (23.3%) White; *n* = 3 (10%) Hispanic; *n* = 1 (3.3%) Other	No co‐dependencies; No comorbidities	NR (NR)	(1) Pioglitazone (45mg 1×/day, pioglitazone/ONA) (2) Placebo (AI, 12 weeks, outpatient, unsupervised, CBT + CM)	NMA: dropout (any reason); extent of use (proportion tests/days; EoT); craving; adherence Narrative: serious adverse events
Schmitz 2021 [207]; USA	110 (31, 26, 26, 27); 109 (30, 26, 26, 27)	46.6 (7.93); 77.3% male; *n* = 84 (76.4%) Black or African America; *n* = 9 (8.2%) White; *n* = 2 (1.8%) more than one race; *n* = 15 (13.6%) unknown or not reported	No co‐dependencies; No comorbidities	NR (NR)	(1) Levodopa + carbidopa (400/100 mg 2×/day, l‐DOPA+/ARA) (2) Levodopa + carbidopa + ropinirole (400/100/1 mg 2×/day, l‐DOPA+/ARA) (3) Levodopa + carbidopa + ropinirole (400/100/2 mg 2×/day, l‐DOPA+/ARA) (4) Placebo (AI, 10 weeks, outpatient, mixed, CBT + CM)	NMA: dropout (any reason); serious adverse events; LDCA; extent of use (proportion tests/days; EoT); adherence Narrative: mortality
Schmitz 2024 [208]; USA	79 (20, 19, 23, 17); 74 (19, 17, 21, 17)	49.37 (8.15); 83.8% male; Asian *n* = 2 (2.7%); Black or African American *n* = 56 (75.7%); White *n* = 10 (13.5%); More than one race *n* = 3 (4.1%); Unknown or NR *n* = 3 (4.1%); Hispanic *n* = 10 (13.5%); Non‐Hispanic *n* = 64 (86.5%)^m^	No co‐dependencies; No comorbidities	NR (NR)	(1) Modafinil + drug counselling (300 mg 1×/day, STIM/ARA) (2) Modafinil + Acceptance and Commitment Therapy (300 mg 1×/day, STIM/ARA) (3) Placebo + Drug Counselling (4) Placebo + Acceptance and Commitment Therapy (AI, 8 weeks, outpatient, mixed, other PSI + CM)	NMA: serious adverse events; extent of use (proportion tests/days; EoT); extent of use (frequency; EoT); adherence Narrative: mortality
Schubiner 2002 [209]; USA	59 (24, 24, 11); NR (NR, NR, NR)	37.05 (6.61)^y^; 89.6% male; NR	No co‐dependencies; All diagnosed with ADHD	NR (NR)	(1) Methylphenidate (30 mg 3×/day, STIM/ARA) (2) Pemoline (NR, STIM/ARA) (3) Placebo (AI, 12 weeks, outpatient, mixed, CBT)	NMA: dropout (any reason); dropout (adverse events); LDCA; extent of use (frequency; EoT) Narrative: serious adverse events; craving
Shearer 2003 [210]; Australia	30 (16, 14); NR (NR, NR)	28 (6); 52% male; NR	80% opioid dependent; No comorbidities	NR (NR)	(1) Dexamfetamine (60 mg 1×/day, STIM/ARA) (2) Placebo (AI, 14 weeks, NR, supervised, NR)	NMA: dropout (any reason); craving Narrative: extent of use (frequency; EoT)
Shoptaw 2002 [211]; USA	70 (35, 35); 69 (34, 35)	36.4 (6.7); 79% male; 39.4% Latino/a; 33.3% African American; 21.7% Caucasian; 5.8% Asian‐Pacific or Native American^m^	No co‐dependencies; No comorbidities	NR (85.5% smoking; 13.0% nasal; 1.4% oral)	(1) Amantadine (100 mg 2×/day, NMDAR‐ANT/GLUT) (2) Placebo (AI, 16 weeks, outpatient, unsupervised, other PSI)	NMA: dropout (any reason); serious adverse events; continuous abstinence (treatment); point abstinence (EoT); point abstinence (follow‐up); LDCA; extent of use (proportion tests/days; EoT); craving Narrative: extent of use (BE level; follow‐up)
Shoptaw 2003 [212]; USA	70 (35, 35); 70 (35, 35)	35.19 (8.07); 68.57% male; White *n* = 14 (20%); African American *n* = 28 (40%); Asian/Other *n* = 2 (2.86%); Latino/a *n* = 26 (37.14%)	No co‐dependencies; No comorbidities	NR (82.9% smoking; 11.4% intranasal; 4.3% injection; 1.4% oral)	(1) Baclofen (20 mg 3×/day, Baclofen/ACV + GABA) (2) Placebo (AI, 16 weeks, outpatient, unsupervised, CBT)	NMA: dropout (any reason); dropout (adverse events); continuous abstinence (treatment); point abstinence (EoT); LDCA; adherence Narrative: serious adverse events; craving
Shoptaw 2004 [213]; USA	23 (11, 12); NR (NR, NR)	39.68 (7.75); 100% male; Caucasian 13.0% (*n* = 3); African American 73.9% (*n* = 17); Hispanic/Latino 13.0% (*n* = 3)	No co‐dependencies; No comorbidities	NR (91.3% smoking; 8.7% inhalation)	(1) Dehydroepiandrosterone (100 mg 1×/day, SEX‐H/ONA) (2) Placebo (AI, 12 weeks, outpatient, unsupervised, other PSI)	NMA: dropout (any reason); continuous abstinence (treatment); extent of use (proportion tests/days; EoT); craving Narrative: serious adverse events; mortality
Shoptaw 2005 [214]; USA	60 (15, 15, 15, 15); NR (NR, NR, NR, NR)	38.13 (6.96); 70% male; African American 60% (*n* = 36); Caucasian 15% (*n* = 9); Hispanic 16.66% (*n* = 10); Other 8.33% (*n* = 5)	No co‐dependencies; No comorbidities	NR (76.6% smoking; 21.6% nasal; 1.7% oral)	(1) Levodopa + carbidopa (100/25 mg 3×/day, l‐DOPA+/ARA) (2) Cabergoline (0.5 mg 1×/week, DA/ARA) (3) Ergoloid mesylates (1 mg 3×/day, ergoloid mesylates/ARA) (4) Placebo (AI, 8 weeks, outpatient, mixed, other PSI)	NMA: dropout (any reason); point abstinence (EoT); extent of use (proportion tests/days; EoT); craving Narrative: extent of use (BE level; EoT)
Shorter 2013 [215]; USA	35 (NR, NR, NR); 30 (8, 9, 13)	48.5 (8.9); 86.7% male; African American 20 (66.7%); Non‐African American 10 (33.3%)	No co‐dependencies; No comorbidities	NR (NR)	(1) Doxazosin (8 mg 1×/day, ADR‐ANT/ABA) (2) Doxazosin (8 mg 1×/day, ADR‐ANT/ABA) (3) Placebo (AI, 13 weeks, outpatient, mixed, CBT)	NMA: dropout (any reason); continuous abstinence (treatment) Narrative: dropout (adverse events); serious adverse events
Sofuoglu 2007 [216]; USA	45 (30, 15); NR (NR, NR)	35.53 (8.04); 100% male; Caucasian 36 (80%); African American 4 (8.9%); Hispanic 5 (11.1%)	Opioid dependent; No comorbidities	Mixed (71.1% smoked; 15.6% snorted; 13.3% intravenous)	(1) Progesterone (300 mg 2×/day, SEX‐H/ONA) (2) Placebo (AI, 9 weeks, outpatient, mixed, other PSI + methadone)	NMA: dropout (any reason) Narrative: serious adverse events
Sofuoglu 2011 [217]; USA	14 (7, 7); NR (NR, NR)	40.1 (10.4); 64.3% male; African American 21.4% (*n* = 3); Native American 7.1% (*n* = 1); Caucasian 71.4% (*n* = 10)	Methadone maintained patients; No comorbidities	NR (NR)	(1) Galantamine (16 mg 1×/day, AChEIs/ONA) (2) Placebo (AI, 8 weeks, outpatient, NR, methadone + CBT)	NMA: dropout (any reason); dropout (adverse events); extent of use (frequency; EoT) Narrative: serious adverse events
Sofuoglu 2017 [218]; USA	106 (35, 37, 34); 94 (33, 33, 28)	38.1 (10.3); 62.3% male; *n* = 26 (24.5%) Black; *n* = 70 (66.0%) White; *n* = 10 (9.4%) Other	Opioid dependent; No comorbidities	NR (NR)	(1) Carvedilol (50 mg 1×/day, ADR‐ANT/ABA) (2) Carvedilol (25 mg 1×/day, ADR‐ANT/ABA) (3) Placebo (AI, 15 weeks, outpatient, mixed, CBT + methadone)	NMA: dropout (any reason); extent of use (proportion tests/days; EoT); adherence Narrative: none
Somoza 2013 [219] (Berezina 2012 [220]); USA	186 (92, 94); NR (NR, NR)	44.8 (7.94); 66.7% male; African American 60.2% (*n* = 112); White 31.2% (*n* = 58); Native American or Alaskan native 1.1% (*n* = 2); Other 7.5% (*n* = 14)	No co‐dependencies; No comorbidities	NR (85.5% smoked or intravenous; 14.5% nasal)	(1) Vigabatrin (1.5 g 2×/day, ACV + MS/ACV + GABA) (2) Placebo (AI, 12 weeks, outpatient, unsupervised, CBT + other PSI + CM)	NMA: dropout (any reason); dropout (adverse events); serious adverse events; mortality; continuous abstinence (treatment); extent of use (proportion tests/days; EoT); craving Narrative: extent of use (BE level; EoT); adherence
Stine 1995 [221]; USA	43 (22, 21); NR (NR, NR)	34.51 (5.49); 86% male; 51% African American; 40% White; 9% Hispanic	No co‐dependencies; No comorbidities	NR (NR)	(1) Mazindol (2 mg 1×/day, STIM/ARA) (2) Placebo (AI, 6 weeks, outpatient, unsupervised, RP)	NMA: dropout (any reason); continuous abstinence (treatment); extent of use (frequency; EoT); extent of use (quantity; EoT) Narrative: craving
Suchting 2021 [222] (de Dios 2025 [223]); USA	108 (21, 44, 43); 107 (21, 43, 43)	46 (8.85); 82.4% male; Hispanic 11.1% (*n* = 12); Black = 75.0% (*n* = 81); White = 13.0% (*n* = 14); American Indian or Alaskan Native = 0.9% (*n* = 1)	No co‐dependencies; No comorbidities	NR (NR)	(1) Citalopram (20 mg 1×/day, SSRIs + OSA/ANTID) (2) Citalopram (40 mg 1×/day, SSRIs + OSA/ANTID) (3) Placebo (AI, 9 weeks, outpatient, unsupervised, CBT + CM)	NMA: dropout (any reason); serious adverse events; continuous abstinence (treatment); LDCA; extent of use (proportion tests/days; EoT); adherence Narrative: none
Tapp 2015 [224]; USA	60 (31, 29); NR (NR, NR)	47.9 (8.42); 86.7% male; African American 64.4% (*n* = 38); Caucasian 32.2% (*n* = 19); American Indian 2.9% (*n* = 1); Hispanic 2.9% (*n* = 1)	No co‐dependencies; No comorbidities	NR (NR)	(1) Quetiapine (400 mg 1×/day, SGAs/ABA) (2) Placebo (AI, 12 weeks, outpatient, unsupervised, CBT)	NMA: dropout (any reason); dropout (adverse events); continuous abstinence (treatment); extent of use (proportion tests/days; EoT); extent of use (frequency; EoT); extent of use (quantity; EoT); craving; adherence Narrative: none
Umbricht 2014 [225] (Rass 2015 [226]); USA	171 (40, 45, 39, 47); NR (NR, NR, NR, NR)	42 (7); 52% male; African American 60%	Opiod dependent; No comorbidities	NR (NR)	(1) Topiramate + CM (150 mg 2×/day, ACV + MS/ACV + GABA) (2) Topiramate (150 mg 2×/day, ACV + MS/ACV + GABA) (3) Placebo + CM (4) Placebo (AI, 19 weeks, outpatient, NR, CM + other PSI + methadone)	NMA: dropout (any reason); dropout (adverse events); LDCA; extent of use (proportion tests/days; EoT) Narrative: serious adverse events; adherence
Venneman 2006 [40]; USA	NR (NR, NR); NR (NR, NR)	34.6 (7, 20–52); 80% male; NR	No co‐dependencies; No comorbidities	NR (NR)	(1) Selegiline (10 mg 1×/day, MA‐AD/ANTID) (2) Placebo (NA, 8 weeks, outpatient, mixed, other PSI)	NMA: none Narrative: none
Walsh 2013 [227]; USA	58 (NR, NR); 50 (25, 25)	42 (1.7); 72% male; African American 68%; Caucasian 32%	No co‐dependencies; No comorbidities	NR (92% smoking; 8% intranasal)	(1) Atomoxetine (80 mg 1×/day, atomoxetine/ARA) (2) Placebo (AI, 12 weeks, outpatient, mixed, CBT + CM)	NMA: dropout (any reason); extent of use (proportion tests/days; EoT) Narrative: serious adverse events; craving
Wardle 2017 [228]; USA	85 (45, 40); 76 (38, 38)	45.53 (8.5); 82% male; White 17.6% (*n* = 15); Black 69.4% (*n* = 59); Hispanic 11.8% (*n* = 10); Other 1.2% (*n* = 1)	No co‐dependencies; No comorbidities	NR (NR)	(1) Levodopa + carbidopa (400/100 mg 2×/day, l‐DOPA+/ARA) (2) Placebo (AI, 12 weeks, outpatient, unsupervised, CM + CBT)	NMA: dropout (any reason); dropout (adverse events); serious adverse events; extent of use (proportion tests/days; EoT); adherence Narrative: none
Ware 2023 [229]; USA	83 (42, 41); 80 (40, 40)	45.2 (9); 72.3% male; American Indian 1.3% (*n* = 1); Black/African American 52.5% (*n* = 42); White/Caucasian 43.8% (*n* = 35); More than one race 2.5% (*n* = 2); and of total sample, Hispanic ethnicity 3.8% (*n* = 3)	Opioid use disorder; No comorbidities	NR (NR)	(1) Bupropion (150 mg 2×/day, NDRIs/ARA) (2) Placebo (M, 20 weeks, outpatient, unsupervised, methadone + RP or AI)	NMA: dropout (any reason); point abstinence (follow‐up); LDCA Narrative: serious adverse events; mortality
Winhusen 2005 [230]; USA	67 (17, 16, 17, 17); NR (NR, NR, NR, NR)	39.6 (5.9); 73% male; 91% African American; 9% Caucasian	No co‐dependencies; No comorbidities	Mixed [97% smoked (crack); 3% intranasal]	(1) Donepezil (10 mg per week, AChEls/ONA) (2) Sertraline (100 mg per week, SSRIs+OSA/ANTID) (3) Tiagabine (20 mg per week, ACV+MS/ACV+GABA) (4) Placebo (AI, 8 weeks, outpatient, unsupervised, CBT)	NMA: dropout (any reason); dropout (adverse events); serious adverse events; extent of use (BE level; EoT); extent of use (frequency; EoT); craving; adherence Narrative: severity of dependence
Winhusen 2007a [231] (Winhusen 2014b [232]); USA	119 (60, 59); NR (NR, NR)	40.95 (7.62); 70.6% male; African American 74.8% (*n* = 89); Caucasian 18.5% (*n* = 22); Hispanic 0.8% (*n* = 1); Other 5.9% (*n* = 7)	No co‐dependencies; No comorbidities	NR (98.3% smoked; 1.7% intranasal)	(1) Reserpine (0.5 mg 1×/day, reserpine/ABA) (2) Placebo (AI, 13 weeks, outpatient, unsupervised, CBT)	NMA: dropout (any reason); dropout (adverse events); extent of use (BE level; EoT); extent of use (proportion tests/days; EoT); craving; adherence Narrative: serious adverse events
Winhusen 2007b [233]; USA	141 (70, 71); NR (NR, NR)	42.5 (7.73); 67.4% male; African American 66.0%; Caucasian 29.1%; Hispanic 2.8%; Native American/Alaskan 1.4%; Other 0.7%	No co‐dependencies; No comorbidities	NR (95% smoked; 0.7% intravenous; 5.7% intranasal; 0.7% oral)	(1) Tiagabine (10 mg 2×/day, ACV + MS/ACV + GABA) (2) Placebo (AI, 15 weeks, outpatient, unsupervised, CM)	NMA: dropout (any reason); dropout (adverse events); serious adverse events; extent of use (proportion tests/days; EoT); craving; adherence Narrative: extent of use (BE level; EoT)
Winhusen 2014a [234]; USA	62 (35, 27); 62 (35, 27)	45.6 (7.3); 62.9% male; African American: 72.6% (*n* = 45); Caucasian 22.6% (*n* = 14); Other/mixed 4.8% (*n* = 3)|*n* = 45 (72.6%) Black/African American, *n* = 14 (22.6%) White, *n* = 3 (4.8%) more than one race	No co‐dependencies; No comorbidities	Crack (NR)	(1) Buspirone (30 mg 2×/day, buspirone/ANTID) (2) Placebo (RP, 16 weeks, mixed, mixed, other PSI + CM)	NMA: dropout (any reason); serious adverse events; LDCA; extent of use (proportion tests/days; EoT); adherence Narrative: dropout (adverse events)
Winstanley 2011 [235]; USA	186 (46, 48, 45, 47); NR (NR, NR, NR, NR)	38.78 (7.78); 54.5% male; White 49.7% (*n* = 72); Black 48.3% (*n* = 70); Other 2.1% (*n* = 3)^z^	Opioid dependent; No comorbidities	NR (NR)	(1) Fluoxetine + CM (60 mg 1×/day, SSRIs + OSA/ANTID) (2) Fluoxetine (60 mg 1×/day, SSRIs + OSA/ANTID) (3) Placebo + CM (4) Placebo (AI, 16 weeks, outpatient, supervised, CM + other PSI)	NMA: dropout (any reason); dropout (adverse events) Narrative: craving
Zhang 2019 [236] (Shorter 2020 [237]); USA	96 (NR, NR); 76 (47, 29)	48.28 (8.36); 73.5% male; 69.8% African American^aa^	No co‐dependencies;	NR (NR)	(1) Doxazosin (8 mg 1×/day, ADR‐ANT/ABA) (2) Placebo (AI, 12 weeks, outpatient, mixed, CBT)	NMA: dropout (adverse events); serious adverse events^ab^ Narrative: dropout (any reason)^ab^

Abbreviations: ABA, antagonist/blocker approaches; AChEI, acetylcholinesterase inhibitors; ACV+GABA, anticonvulsants and GABAergic agents; ACV+MS, anticonvulsants and mood stabilizers; ADHD, Attention Deficit Hyperactivity Disorder; AI, abstinence initiation; ANTID, antidepressants and other serotonergic agents; ARA, agonist/replacement approaches; BE, benzoylecgonine; CBT, cognitive behavioural therapy; CM, contingency management; COMBO, combination pharmacotherapy; DAM, pharmaceutical‐grade diacetyl‐morphine; l‐DOPA+, levodopa combined with other agents; EoT, end of treatment; GABA, gamma‐aminobutyric acid; GLUT, glutamate modulators; HAM‐D, Hamilton Depression Rating Scale; LAAM, levo‐alpha‐acetylmethadol; LDCA, longest duration of continuous abstinence; MA‐AD, mixed action antidepressants; NMA, network meta‐analysis; NMDAR‐ANT, N‐methyl‐D‐aspartate receptor antagonists; NR, not reported; OAA, opioid agonists and antagonists; OAT, opioid agonist treatment; ONA, other neuroactive agents; PSI, psychosocial interventions; RP, relapse prevention therapy; SSRI, selective serotonin reuptake inhibitor; STIM, stimulants.

^a^ Primary studies are referenced first, with secondary studies in brackets (where applicable).

^b^ Only includes participants randomised to eligible study arms.

^c^ Author‐defined ‘treated’ sample (e.g. number receiving first dose, number remaining after first week, etc.).

^d^ Author‐defined ethnicity.

^e^ Only listed when applies to full sample (i.e. was part of the inclusion criteria).

^f^ Only includes intervention arms eligible for the current review.

^g^ Maximum dose.

^h^ Two categories of pharmacotherapy as used in analyses for each drug: fine class and course class. For definitions and descriptions, see Appendix S4.

^i^ Intervention type refers to whether the intervention aimed to address abstinence initiation (AI), relapse prevention (RP) or mixed elements of both (M).

^j^ Time actively taking medication (including dose escalation and titration).

^k^ Supervised: all doses observed by trial personnel; unsupervised: all doses taken at home; mixed: some doses observed.

^l^ *n* analysed.

^m^ Demographics only reported for the ‘treated’ sample.

^n^ Demographics only reported for disulfiram group.

^o^ Extent of use outcome was extracted from Streeter [68].

^p^ Demographics only reported for those who completed treatment.

^q^ Adherence outcome was extracted from Ehrman [72] (subset).

^r^ Secondary to unknown primary study.

^s^ Demographics only reported for the initial 71 participants recruited in this trial.

^t^ Descriptive statistics only reported for the 22 participants that completed both cue‐reactivity sessions.

^u^ Categories not mutually exclusive.

^v^ Extent of use (proportion tests/days; EoT) outcome extracted from Schmitz 2009.

^w^ Dropout (any reason); serious adverse events; extent of use (frequency; EoT) and extent of use (quantity; EoT) outcomes were extracted from Milvojevic [181].

^x^ Demographics only reported for the 63 participants recruited at one of two recruiting sites.

^y^ Age mean/SD does not include the 11 randomised to a dropped arm.

^z^ Demographics only reported for the 145 who were retained at week 8.

^aa^ Demographics only reported for the patients with depression (*n* = 20).

^ab^ All outcomes were extracted from Shorter [237].

In total, 89 unique medications were included across all studies. As in previous reviews of pharmacological treatments for SUDs, we grouped medications according to type and mechanism of action for the purpose of the analysis [238]. Two versions of drug categories were created: (i) therapeutic strategy; and (ii) mechanistic. The therapeutic strategy categorisation pragmatically consolidated medications into a smaller set of broader intervention nodes, consisting of eight classes in total (Appendix S5). In developing these categories, we sought to preserve major therapeutic approaches relevant to CUD (e.g. dopaminergic agonist/replacement strategies or dopamine‐blocking/antagonist approaches) [239] and to reflect the focus of previous reviews (e.g. anticonvulsants, antidepressants) [21, 240]. Where trials used medications that did not fit into existing node structures, or combined medications from multiple nodes, they were grouped as *combination pharmacotherapy* (Appendix S5).

The mechanistic categorisation captured 39 mechanistic groupings that clustered related pharmacological classes, such as *N*‐methyl‐d‐aspartate (NMDA) receptor antagonists and opioid agonist therapies. Further details, including lists of medications within each therapeutic strategy and the mechanistic category are also provided in Appendix S5. This dual approach aimed to preserve pharmacological similarity in how interventions were grouped, while pragmatically consolidating nodes to support network connectivity and facilitate NMA. Results from the mechanistic categorisation are not presented in the main text as the large number of nodes with small study counts limits interpretability, although we have presented them in the supporting information, for completeness (Appendix S6).

### Outcomes

Results for primary outcomes are presented in the text. Quantitative data were available for all outcomes. We conducted NMA for all outcomes except ‘extent of use at follow‐up [urine benzoylecgonine (BE) levels]’ and ‘severity of dependence’, where insufficient data were available for analyses. Where outcomes were reported in a format that was not suitable for NMA or insufficient data were available, we present results narratively in Appendix S8.

### Risk of bias

We assessed a total of 590 results included in the NMAs. We judged 48% to be at high risk of bias, 36% to have some concerns and 16% to be at low risk of bias. The principal concerns were risk of bias through a failure to follow intention‐to‐treat principles in the analysis (covered in the domain on deviations from the intended interventions) and risk of bias associated with missing outcome data. Detailed assessments for each outcome are presented in Appendix S7.

### Results of the syntheses

We considered transitivity across the included studies to be plausible, so we conducted NMAs on outcomes for which the available comparisons formed a connected network. Results from random‐effects models are presented for most outcomes. Unless otherwise stated, the results from common‐effect sensitivity analyses and direct estimates from inconsistency random‐effects models were consistent with the primary NMAs. For models with fewer than five degrees of freedom for estimating the level of heterogeneity, results of common‐effect models are presented without sensitivity analyses. The R‐hat values were all <1.01, suggesting good convergence. Network plots for all analyses are presented in Appendix S9. We conducted subgroup analyses by intervention type (abstinence initiation vs relapse prevention) and by presence of co‐occurring SUD/comorbidity for continuous abstinence during treatment, point abstinence at end of treatment and dropout for any reason. Several pre‐specified subgroup analyses were not possible. The predominant cocaine type used by participants was seldom reported. Around 95% of studies employed co‐occurring psychosocial interventions (e.g. counselling), and the vast majority of studies were conducted in unsupervised, outpatient settings, which prevented meaningful subgroup analysis.

Results are primarily expressed relative to placebo and relative effects for all outcomes are available in Appendix S9. In line with the CINeMA approach of considering outcomes in the context of an MCID, we estimated the probability (reported as *P*) that the relative effect exceeded an MCID by calculating the proportion of posterior draws above or below that value from each NMA (depending on the direction of beneficial effect). The results within the article represent the therapeutic strategy categorisation. Results from the mechanistic categorisation are presented in Appendix S6.

#### Effectiveness

##### Continuous abstinence during treatment

The NMA consisted of 46 studies of nine classes, including direct evidence from a maximum of 11 studies per contrast (antidepressants and other serotonergic agents). Periods of continuous abstinence ranged from 1 to 16 weeks, but were most commonly set as 3 weeks (29/46 studies). The majority of studies (25/46) assessed a period of continuous abstinence up to and including the end of treatment, with the remaining 21 studies assessing a period of continuous abstinence at any point during the treatment period. Results of random‐effects NMA suggest that antidepressants and other serotonergic agents (OR = 1.74, 95% CrI = 1.02–3.13, *P* = 0.89) and combination pharmacotherapy (OR = 4.38, 95% CrI = 2.04–9.94, *P* = 0.99) may have beneficial effects relative to placebo (Figure 2a). Other placebo comparisons were consistent with no effect. The relative effects of different active treatments suggest that combination pharmacotherapy may be more beneficial than all treatments, except anticonvulsants and GABAergic agents and agonist/replacement approaches. All other contrasts between interventions were consistent with the possibility of no effect. All estimates were rated as very low certainty.

**FIGURE 2:**
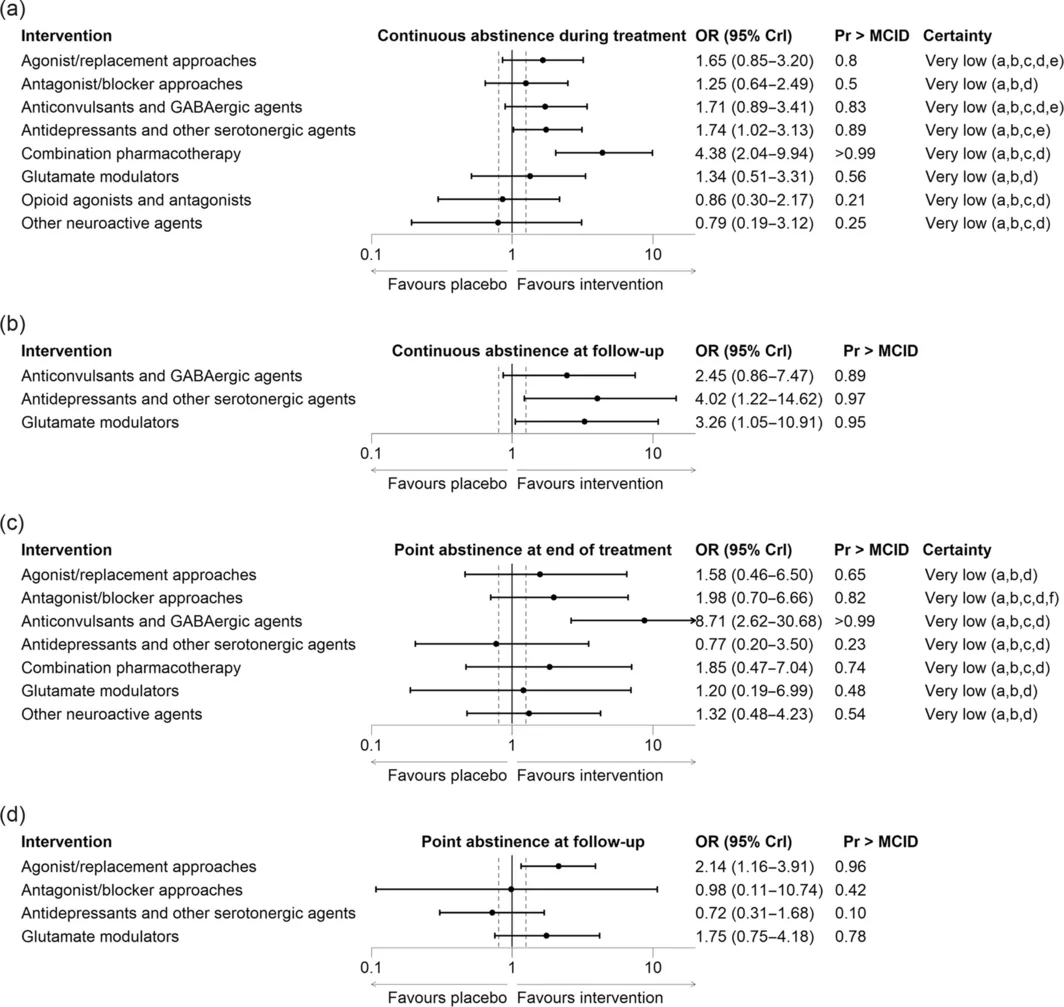
Forest plots of primary effectiveness outcomes: (a) continuous abstinence during treatment; (b) continuous abstinence at follow‐up; (c) point abstinence at end of treatment; (d) point abstinence at follow‐up.

The results of subgroup analysis by presence of co‐occurring SUD/comorbidity (Figure 3a) suggest that combination pharmacotherapy may be beneficial in both groups, and antidepressants and other serotonergic agents may be more beneficial in those with co‐occurring SUD/comorbidity (OR = 2.27, 95% CrI = 1.00–5.45) than in those without (OR = 1.19, 95% CrI = 0.48–2.89). Subgroup analysis by intervention type (Figure 3b) suggests that combination pharmacotherapy may have a beneficial effect relative to placebo in abstinence initiation studies (OR = 5.16, 95% CrI = 2.39–11.7), but there was substantial uncertainty in relapse prevention studies (OR = 2.70, 95% CrI = 0.56–13.8). Additionally, whilst agonist/replacement approaches may have a beneficial effect in abstinence initiation studies relative to placebo (OR = 2.19, 95% CrI = 1.23–4.01), this effect, although imprecise, may be reversed in relapse prevention studies, resulting in reduced abstinence relative to placebo (OR = 0.03, 95% CrI = 0.00–0.32).

**FIGURE 3:**
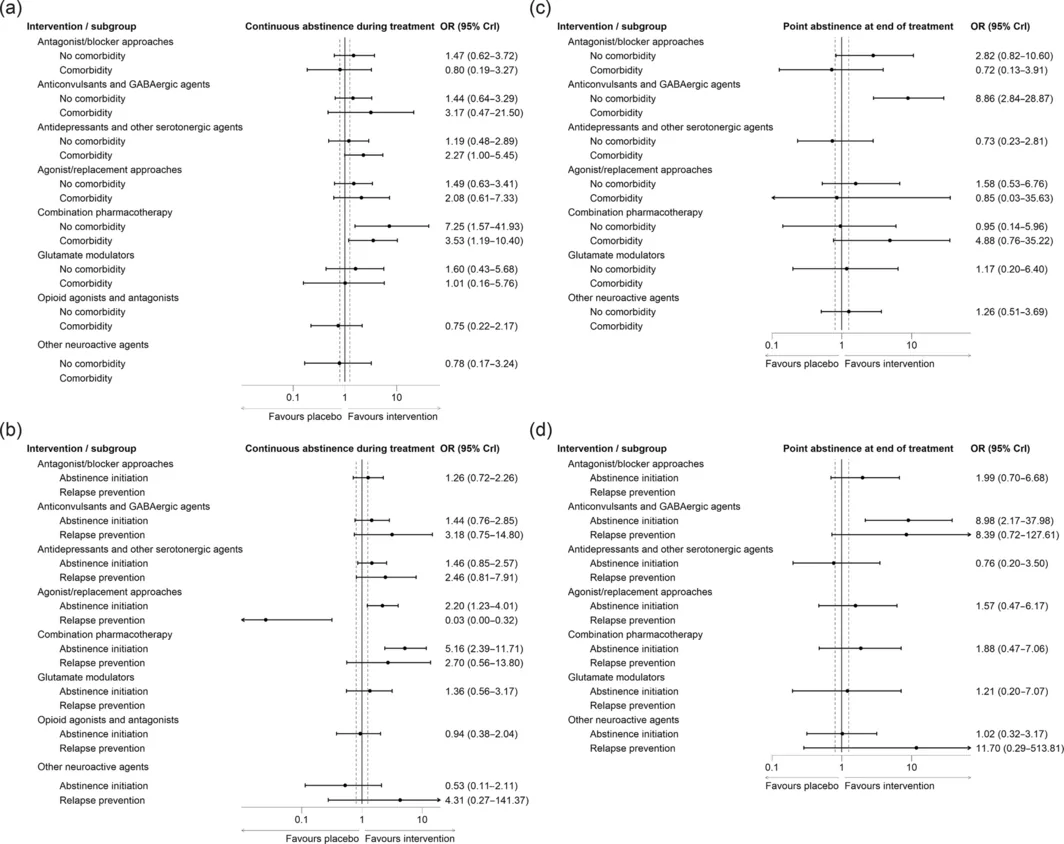
Forest plots of subgroup analyses for primary effectiveness outcomes: (a) continuous abstinence during treatment by presence of co‐occurring substance use disorder (SUD)/comorbidity; (b) continuous abstinence during treatment by intervention type; (c) point abstinence at end of treatment by presence of co‐occurring SUD/comorbidity; (d) point abstinence at end of treatment by intervention type.

##### Continuous abstinence at follow‐up

The NMA consisted of five studies of four classes, including direct evidence from a maximum of three studies per contrast (glutamate modulators). Studies assessed continuous periods of abstinence ranging from 2 to 24 weeks. Results of the common‐effect NMA suggest that antidepressants and other serotonergic agents (OR = 4.02, 95% CrI = 1.22–14.6) and glutamate modulators (OR = 3.26, 95% CrI = 1.05–10.9) may have beneficial effects relative to placebo (Figure 2b).

##### Point abstinence at end of treatment

The NMA consisted of 22 studies of eight classes, including direct evidence from a maximum of six studies per contrast (agonist/replacement approaches). All measures were confirmed with urinalysis and represented abstinence at the final assessment point during the treatment period. The results of the random‐effects NMA suggest that anticonvulsants and GABAergic agents may lead to meaningful improvements relative to placebo (OR = 8.71, 95% CrI = 2.62–30.7, *P* = 0.99) (Figure 2c). All other placebo and active treatment comparisons were consistent with the possibility of no effect relative to placebo. All estimates were rated as very low certainty.

The results of subgroup analyses were consistent with the primary models for studies in people without co‐occurring SUD/comorbidity and abstinence initiation studies (Figure 3c,d). Owing to very few relapse prevention studies and studies in people with co‐occurring SUD/comorbidity, the effect estimates in these subgroups were very imprecise.

##### Point abstinence at end of follow‐up

The NMA consisted of seven studies of five classes, including direct evidence from a maximum of three studies per contrast (glutamate modulators). Results of the common‐effect NMA suggest that agonist/replacement approaches may have beneficial effects relative to placebo (OR = 2.14, 95% CrI = 1.16–3.91) (Figure 2d). All other estimates showed evidence consistent with the possibility of no effect.

#### Acceptability

##### Dropout for any reason

The NMA consisted of 135 studies of nine classes, including direct evidence from a maximum of 34 studies per contrast (agonist/replacement approaches). All estimates were rated as very low certainty. All placebo comparisons from the random‐effects NMA showed evidence consistent with the possibility of no effect (Figure 4). The relative effects of different active treatments suggest that antagonist/blocker approaches may reduce dropout compared with other neuroactive agents, while all other contrasts were consistent with the possibility of no effect.

**FIGURE 4:**
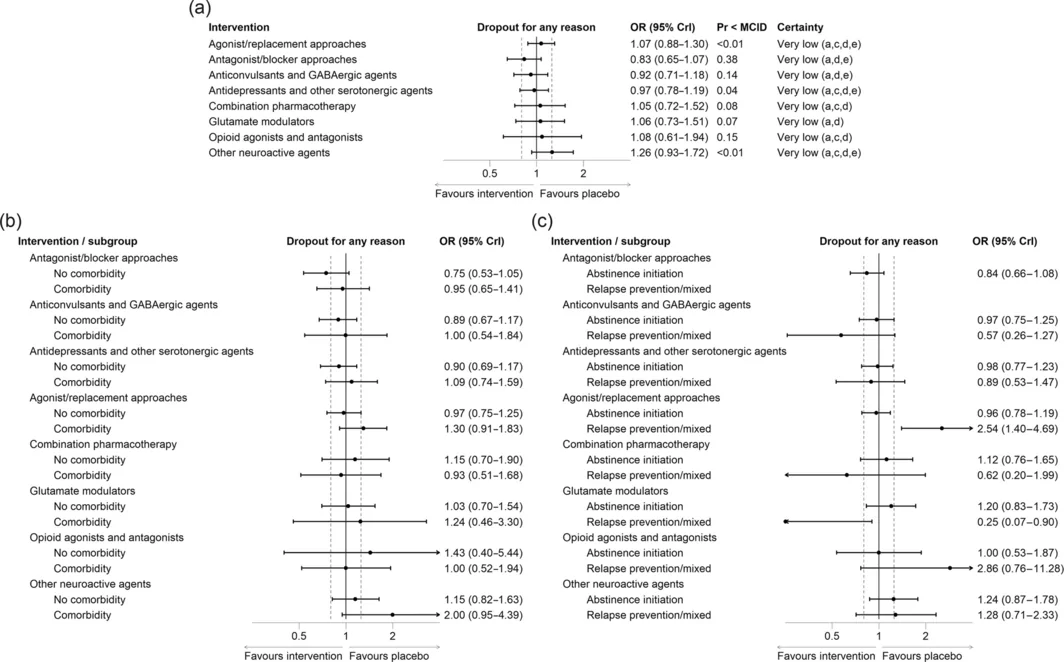
Forest plots of primary acceptability outcome—dropout for any reason—with subgroup analyses: (a) primary model; (b) subgroup by presence of co‐occurring substance use disorder (SUD)/comorbidity; (c) subgroup by intervention type.

The results of subgroup analysis by presence of co‐occurring SUD/comorbidity were consistent with the results of the main analysis (Figure 4b). The results from intervention type subgroup analysis were largely consistent with the primary model, with the exception of agonist/replacement approaches. In contrast to the primary analysis, agonist/replacement approaches appeared to result in greater dropout than placebo in relapse prevention/mixed studies (Figure 4c).

#### Safety

##### Serious adverse events

The NMA consisted of 41 studies of nine classes, including direct evidence from a maximum of 10 studies per contrast (antidepressants and other serotonergic agents). The number of reported serious adverse events across studies, and subsequently different nodes, was typically low, contributing to imprecise estimates with wide 95% CrIs. No consistent evidence of effect was observed across any placebo or active treatment comparisons, either in the primary or in the subgroup analyses Figure 5a–c.

**FIGURE 5:**
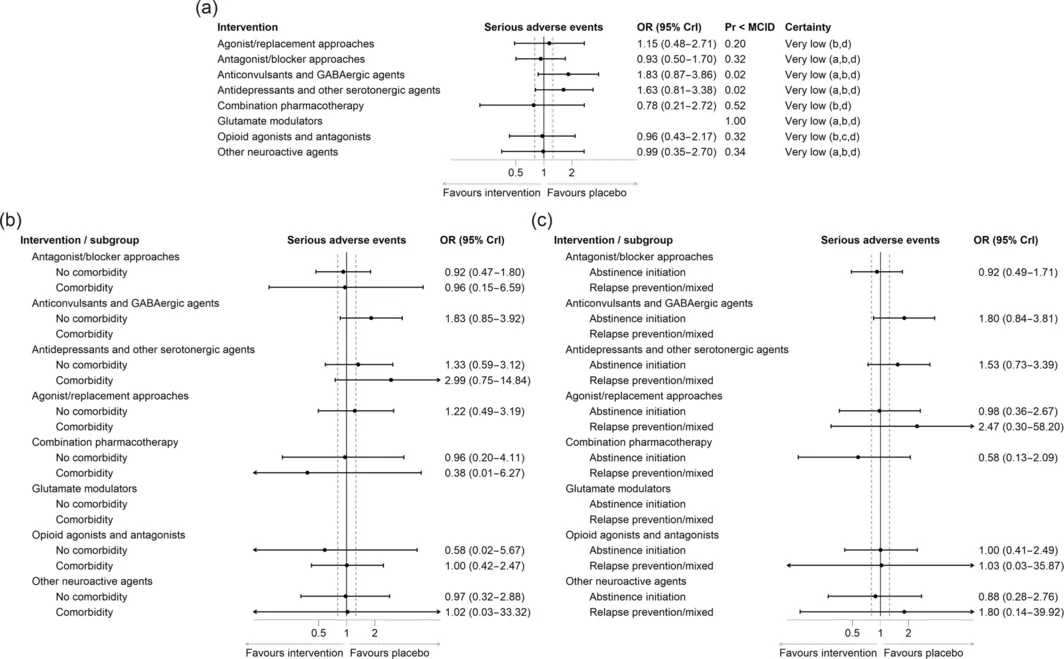
Forest plots of primary safety outcome—serious adverse events—with subgroup analyses: (a) primary model; (b) subgroup by presence of co‐occurring substance use disorder (SUD)/comorbidity; (c) subgroup by intervention type.

The results for all secondary effectiveness, acceptability and safety outcomes are summarised in Table 3 and Figures 6, 7, 8.

**TABLE 3:** Secondary outcomes.

Outcome	*K* in NMA (classes in NMA) NMA model	Notable findings	Risk of bias
**Effectiveness**			
Longest duration of continuous abstinence	19 (6) RE	Primarily reported as ‘days of continuous abstinence’ (17/19 studies), with a minority of studies reporting the longest sequence of negative tests (2/19). As such, all SMD estimates are re‐expressed as MDs in days of continuous abstinence. NMA included direct evidence from a maximum of 9 studies per contrast (agonist/replacement approaches). All placebo and active treatment comparisons from random‐effects NMA showed evidence consistent with the possibility of no effect relative to placebo (Figure 6a)	55% High 45% SC 0% Low
Extent of use: frequency (EoT)	40 (9) RE	NMA included direct evidence from a maximum of 10 studies per contrast (antagonist/blocker approaches and other neuroactive agents). SMD estimates are re‐expressed as MDs in proportion of days using cocaine during treatment period. Results of random‐effects NMA suggest that anticonvulsants and GABAergic agents may lead to meaningful reductions in frequency of cocaine use relative to placebo (MD = –5.94%, 95% CrI = −12.0 to 0.00; *P* = 0.52). All other placebo comparisons were consistent with the possibility of no effect (Figure 6b)	65% High 35% SC 0% Low
Extent of use: frequency (follow‐up)	7 (5) CE	NMA included direct evidence from a maximum of two studies per contrast (glutamate modulators, other neuroactive agents and antagonist/blocker approaches). SMD estimates are re‐expressed as MDs in proportion of days using cocaine during follow‐up period. Results of common‐effect NMA suggest that anticonvulsants and GABAergic agents may lead to reductions in the frequency of cocaine use relative to placebo (MD = –15.7%, 95% CrI = −30.6 to −0.81%, *P* = 0.87). All other placebo and active treatment comparisons were consistent with the possibility of no effect (Figure 6c)	0% High 100% SC 0% Low
Extent of use: quantity (EoT)	19 (8) RE	NMA included direct evidence from a maximum of 6 studies per contrast (antagonist/blocker approaches). SMD estimates are re‐expressed as MD in grams of cocaine used per week during treatment period. All placebo and active treatment comparisons from random‐effects NMA showed evidence consistent with the possibility of no effect (Figure 6d)	48% High 52% SC 0% Low
Extent of use: quantity (follow‐up)	4 (5) CE	SMD estimates are re‐expressed as MDs in grams of cocaine used per week during treatment period. All placebo and active treatment comparisons from common‐effect NMA showed evidence consistent with the possibility of no effect (Figure 6e)	60% High 40% SC 0% Low
Extent of use: BE levels	12 (6) RE	NMA included direct evidence from a maximum of five studies per contrast (anticonvulsants and GABAergic agents). All placebo and active treatment comparisons from random‐effects NMA showed evidence consistent with the possibility of no effect (Figure 7a).	87% High 13% SC 0% Low
Extent of use: proportion of UDS	45 (8) RE	NMA included direct evidence from a maximum of 19 studies per contrast (agonist/replacement approaches). All estimates from random‐effects NMA showed evidence consistent with the possibility of no effect relative to placebo except other neuroactive agents, which may be detrimental relative to placebo (MD = 16%, 95% CrI = 1.47–31.2) (Figure 7b). All relative effects of different active treatments showed evidence consistent with the possibility of no effect	52% High 44% SC 4% Low
Craving	39 (8) RE	NMA included direct evidence from a maximum of 14 studies per contrast (antidepressants and other serotonergic agents). SMD estimates are re‐expressed as MDs in points on the Brief Substance Craving Scale. Results of random‐effects NMA suggest that antidepressants and other serotonergic agents may lead to reductions in craving relative to placebo (MD = –0.58, 95% CrI = −1.22 to 0.09, *P* = 0.54) (Figure 7c). All other placebo and active treatment comparisons were consistent with the possibility of no effect	80% High 20% SC 0% Low
**Acceptability**			
Dropout for adverse events	39 (9) RE	NMA included direct evidence from a maximum of 11 studies per contrast (agonist/replacement approaches). Results of random‐effects NMA suggest that antagonist/blocker approaches may lead to increased dropout owing to adverse events compared with placebo (OR = 3.19, 95% CrI = 1.21–9.69, *P* = 0.99). All other placebo comparisons were consistent with the possibility of no effect (Figure 8a). Relative effects of different active treatments suggest that antagonist/blocker approaches may lead to increased dropout relative to agonist/replacement approaches, while all other contrasts were consistent with the possibility of no effect	61% High 37% SC 2% Low
Adherence	52 (9) RE	NMA included direct evidence from a maximum of 15 studies per contrast (agonist/replacement approaches). SMD estimates are re‐expressed as MDs in proportion of medication taken. Results of random‐effects NMA suggest that no treatments are likely to have a beneficial effect on adherence relative to placebo; however, anticonvulsants and GABAergic agents may lead to reduced adherence (MD = –3.32%, 95% CrI = −6.36 to 0.00). All other placebo comparisons were consistent with the possibility of no effect (Figure 8b). Relative effects of different active treatments suggest that antidepressants and other serotonergic agents may be more beneficial for adherence than anticonvulsants and GABAergic agents, while all other contrasts were consistent with the possibility of no effect	61% High 39% SC 0% Low
**Safety**			
Mortality	4 (5) CE	Only four deaths were reported across all trials. Consequently, the NMA estimates were extremely imprecise with wide 95% CrIs. No consistent evidence of effect was observed across any placebo or active treatment comparisons	–

Abbreviations: BE, benzoylecgonine; CE, common effect; CrI, credible interval; CUD, cocaine use disorder; EoT, end of treatment; *K*, number of studies; MD, mean difference; NMA, network meta‐analysis; OR, odds ratio; *P*, probability; RE, random effects; SC, some concerns; SMD, standardised mean difference; UDS, urine drug screen.

**FIGURE 6:**
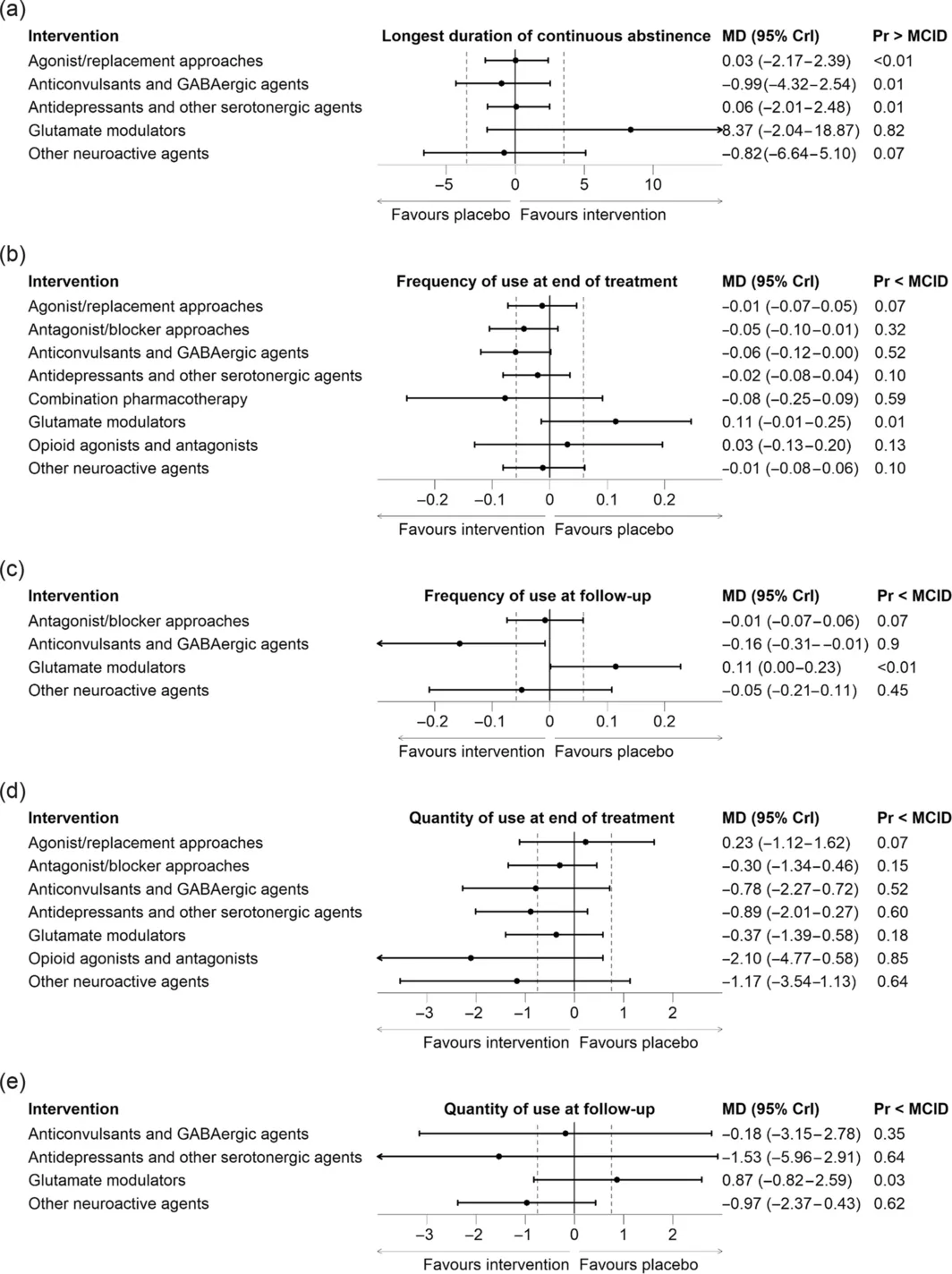
Forest plots of secondary effectiveness outcomes: (a) longest duration of continuous abstinence; (b) extent of use—frequency at end of treatment; (c) extent of use—frequency at end of follow‐up; (d) extent of use—quantity at end of treatment; (E) extent of use—quantity at end of follow‐up.

**FIGURE 7:**
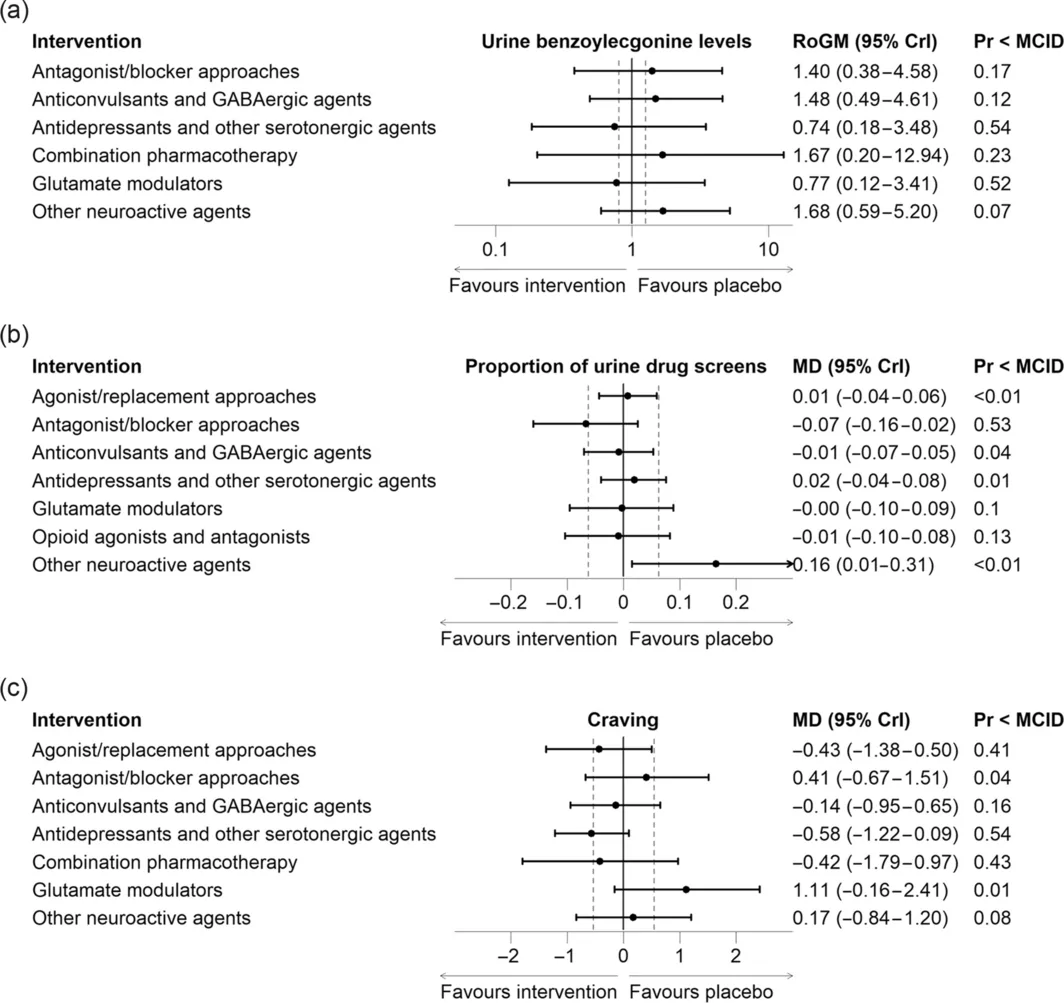
Forest plots of secondary effectiveness outcomes: (a) extent of use—urine benzoylecgonine levels; (b) extent of use—proportion of urine drug screens; (c) craving.

**FIGURE 8:**
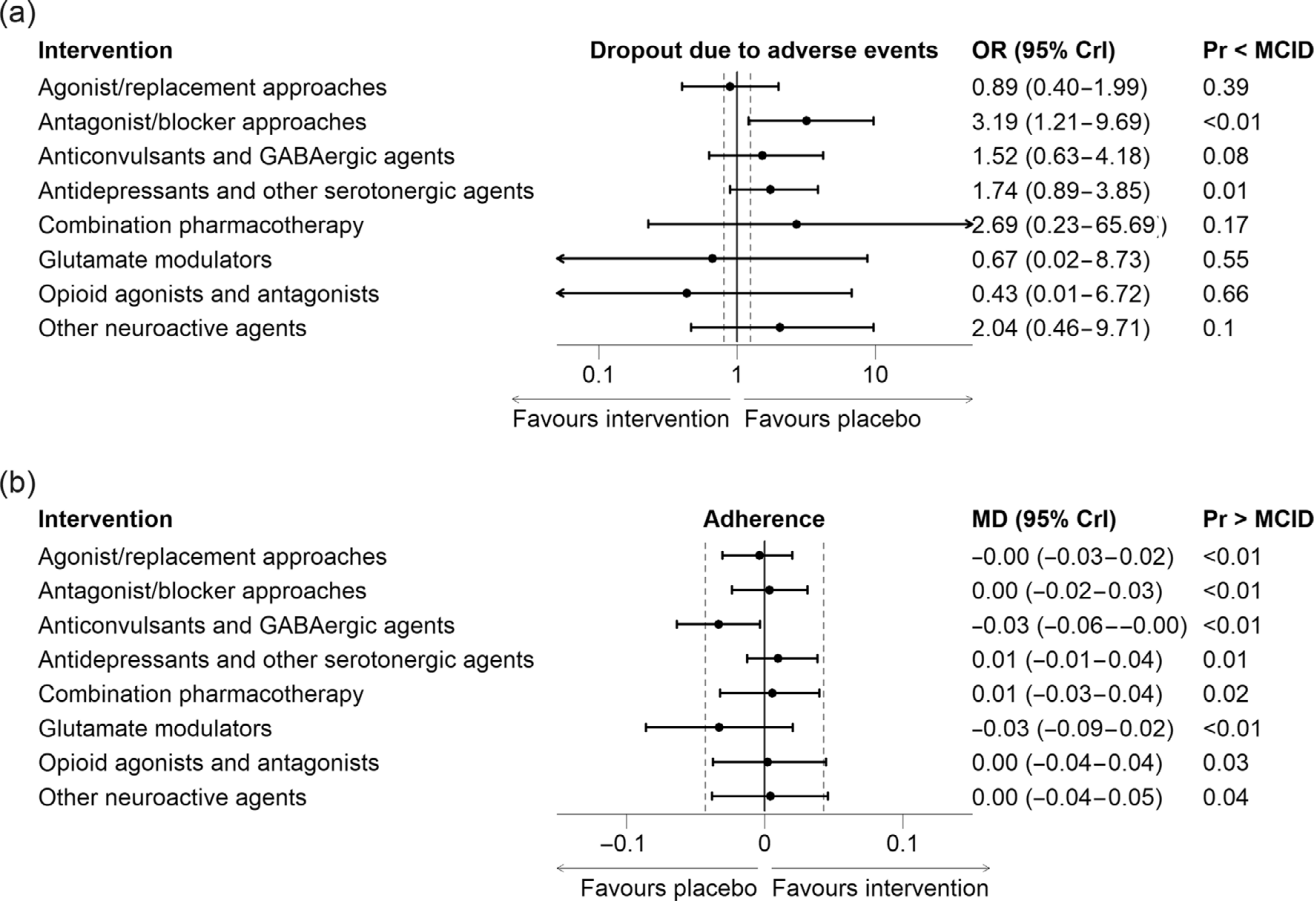
Forest plots of secondary acceptability outcomes: (a) dropout owing to adverse events; (b) adherence.

## DISCUSSION

### Summary of findings

Our systematic review and NMA sought to determine the comparative effectiveness, safety and acceptability of different pharmacological interventions for the treatment of CUD and the prevention of relapse. The 163 included RCTs with 14 871 participants evaluated 89 pharmacological agents, and our NMAs addressed 12 effectiveness outcomes, three acceptability outcomes and two safety outcomes. Overall, there were limited and inconsistent signals from the data across outcomes, with the certainty of evidence ranging from very low to low because of concerns regarding the risk of bias and imprecise and heterogenous results.

#### Effectiveness

The mixed signals from effectiveness outcomes, in tandem with risk of bias concerns and very low certainty, cast doubt on any potentially promising results for a given therapeutic strategy. For example, antidepressants and other serotonergic agents show a potentially promising effect for continuous abstinence during treatment and follow‐up. However, there is limited evidence of any effect for point abstinence or any extent of use outcomes (Figure 9). These findings seem incompatible, as one would expect that if continuous abstinence was achieved for a specified period of follow‐up, this beneficial effect would also be seen for point abstinence in the same time period (although this would not necessarily be expected in reverse). Similarly, anticonvulsants and GABAergic agents show promising results for point abstinence at end of treatment and follow‐up, but we do not see such benefit across any extent of use outcomes. Even if none of these outcomes directly predict long‐term changes in cocaine use behaviour, we would expect some congruence between related outcomes on this acute time scale. These divergent findings across effectiveness outcomes may be evidence of limited construct validity, or they may be indicative of spurious or chance findings. As such, any conclusions should be made with caution.

**FIGURE 9:**
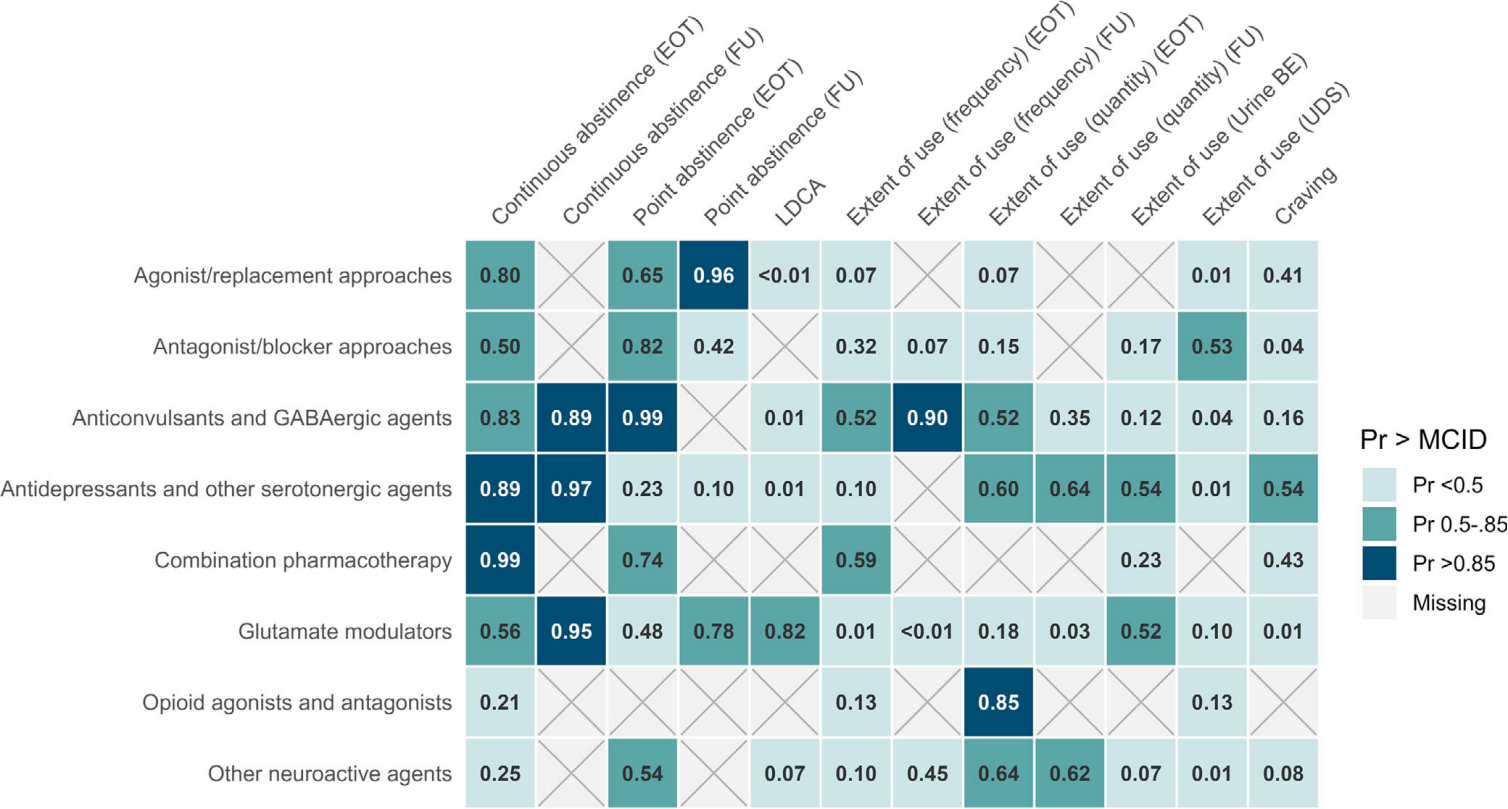
Heat map of probabilities that point estimates for effectiveness outcomes exceed minimum clinically important differences.

Our findings are broadly consistent with, but extend beyond, those of Bentzley *et al*. [11], the largest and most recent previous review of this literature. Bentzley *et al*. identified 157 studies and focused on end‐of‐treatment urinalysis, showing potentially positive signals for some pharmacological treatment strategies that did not persist following imputation and sensitivity analyses. Our investigation of a broader set of clinically relevant effectiveness outcomes found similarly inconsistent signals that did not generalise across related outcome measures. Our findings, in tandem with those of Bentzley *et al*., reinforce concerns that isolated positive findings may not represent reliable intervention effects.

Several treatment strategies included in this review were evaluated based on plausible physiological mechanisms, including the modulation of dopaminergic, serotonergic or GABAergic pathways. The absence of consistent effects across related effectiveness outcomes suggests that such mechanistic hypotheses have not translated into reliable clinical benefits.

#### Acceptability

Our primary analysis does not suggest that any one medication type is likely to influence dropout. All estimates were graded as very low certainty, owing to risk of bias, imprecision and indirectness concerns. We found no evidence that the presence or absence of comorbidities affected the relative acceptability of pharmacotherapies. However, analysis by intervention type did suggest that the agonist/replacement approaches used in relapse prevention studies may result in increased dropout relative to placebo, but the reason for this is unclear.

#### Safety

Collectively, the results from this review do not suggest that any particular class of drugs is likely to be associated with an increased incidence of serious adverse events or mortality. This is consistent with previous meta‐analyses of different medication types, but the low number of events prevented precise estimations of intervention effects, such that any inferences should be made with caution.

### Strengths and limitations of the review

To our knowledge, this is the most comprehensive review and NMA of pharmacological interventions for CUD to date. Previous reviews have filtered by drug type [16] and restricted their synthesis to a single or a limited set of outcomes [11, 241]. By contrast, our review assessed a broad range of effectiveness, safety and acceptability outcomes from double‐blinded RCTs without limiting by medication type or by subpopulation of those living with CUD. We also applied NMA methods to enable the simultaneous synthesis of different classes of drugs, which is the recommended approach when the evidence base consists of numerous diverse therapeutic approaches.

Despite the rigorous methods employed in this review, several limitations stem from weaknesses with the broader evidence base. First, we grouped studies by therapeutic strategy to facilitate the NMA, which may have missed some nuance afforded by differences in dosing or exact mechanism of action [22]. The 163 identified studies assessed 89 unique medications, reflecting minimal replication across this research area. The aim of consolidating studies into broader therapeutic strategies was to maximise our ability to detect potential signals that might better inform future research directions. However, the lack of consistent or compelling evidence across outcomes means we are unable to identify any therapeutic strategy warranting prioritisation. For completeness, the results of a more granular grouping approach, informed by mechanism of action are presented in the supporting information (Appendix S6). Second, in addition to heterogenous interventions, studies assessed broad and inconsistent effectiveness outcomes, which made synthesis challenging. Such diverse outcomes are perhaps understandable in the absence of established proxy outcomes for long‐term behaviour change in people living with CUD. However, the lack of standardised outcomes may dilute the evidence base with outcomes that do not truly represent marked changes in cocaine use or lasting behaviour change. Third, whilst we planned to conduct subgroup analyses by the presence of co‐occurring SUD/comorbidity and predominant cocaine type, in practice very few trials adequately reported these factors and thus we were unable to fully explore these potential differences. Fourth, publication bias is a threat to all literature‐based systematic reviews. The lack of convincing evidence for the effectiveness of any intervention suggests that publication bias favouring small *P*‐values or large effects—as it is usually expected to operate—was not an important driver of our findings. Finally, data extraction and risk of bias assessments were conducted by one of three reviewers and checked by a second reviewer, rather than independently and in duplicate, which may have increased the risk of error. However, we used structured guidance documents and regular team discussions throughout the process to attenuate this risk.

### Implications for future research

This review highlights a lack of coherence across pharmacological trials for CUD. The absence of validated proxy measures for long‐term behaviour change in people living with CUD has led to the use of heterogeneous and incongruent indicators of effectiveness. Equally, demographic and clinical characteristics are typically poorly and inconsistently reported. Only a small subset of included trials explicitly restricted enrolment to participants with co‐occurring mental health disorders, whereas most enrolled broader CUD populations. Given the high prevalence of co‐occurring substance use and mental health disorders among individuals with CUD [242], and the potentially different treatment needs for people using different types of cocaine (i.e. powder or crack) [243], the under‐reporting or the exclusion of such factors reduces the practicality and generalisability of the findings. Additionally, the limited or inconsistent reporting limits the ability to determine whether node‐ or outcome‐specific signals reflect direct effects on cocaine use, indirect effects mediated through mood symptoms or both. Establishing core outcomes and demographic reporting standards would enable greater comparability across trials, a more thorough investigation of subgroups within this population and facilitate stronger conclusions.

Future work may also consider investigating different treatment paradigms. Most studies in this review were classified as abstinence initiation studies, whereby participants are actively using cocaine at the time of enrolment and the therapeutic goals are initiating and maintaining abstinence. However, for some SUDs, one approach is to employ an in‐patient detoxification period before using pharmacological therapies to prevent relapse (rather than initiate abstinence) [244]. Based on input from clinical experts, we had planned to compare findings from the two different treatment paradigms in a subgroup analysis; however, the paucity of studies using a relapse prevention design prohibited this. Future research should address this evidence gap in pharmacological treatments for the longer‐term prevention of relapse in people who have achieved initial abstinence from cocaine.

## CONCLUSION

The results from this systematic review and the NMAs suggest that there is currently little compelling evidence for the adoption of pharmacological treatments for CUD. Whilst some treatment strategies such as antidepressants and other serotonergic agents and anticonvulsants and GABAergic agents showed promise for some effectiveness outcomes, these beneficial findings were not consistent across all effectiveness outcomes. There was limited evidence regarding the safety of different pharmacotherapies, and some were associated with poor acceptability. Additionally, all estimates were rated as ‘very low’ or ‘low’ certainty, with common concerns around bias. Future work should seek to develop a set of core outcomes and demographics to improve the congruency of future research.

## AUTHOR CONTRIBUTIONS

**Craig Paterson:** Data curation; investigation; methodology; project administration; visualization; writing—original draft; writing—review and editing; validation. **Thomas Parkhouse:** Investigation; data curation; validation; writing—original draft; writing—review and editing; methodology. **Chloe Burke:** Writing—original draft; writing—review and editing; investigation; validation; data curation; methodology. **Monika Halicka:** Data curation; methodology; investigation; formal analysis; writing—review and editing; software; visualization. **Jennifer C Palmer:** Methodology; data curation; investigation; writing—review and editing. **Hend Gabr:** Writing—review and editing; formal analysis; visualization; data curation; investigation. **Rebecca Wilson:** Investigation; data curation; writing—review and editing. **Katie E Webster:** Investigation; data curation; writing—review and editing. **Francesca Spiga:** Writing—review and editing; investigation; data curation; conceptualization. **Sarah Dawson:** Investigation; data curation; writing—review and editing. **Deborah M Caldwell:** Writing—review and editing; conceptualization; supervision; funding acquisition. **Jennifer Scott:** Conceptualization; methodology; supervision; writing—review and editing. **Julian PT Higgins:** Funding acquisition; writing—review and editing; methodology; conceptualization; supervision. **Jelena Savović:** Supervision; funding acquisition; conceptualization; methodology; writing—review and editing.

## DECLARATION OF INTERESTS

None to declare.

## SYSTEMATIC REVIEW REGISTRATION

PROSPERO (Protocol Registration for Systematic Reviews): CRD42024596434.

## Supporting information


**Appendix S1.** Hierarchy for extracting outcomes with multiple data points.
**Appendix S2.** Literature search strategy.
**Appendix S3.** Data preparation methods.
**Appendix S4.** Plain language statistical explanation, additional analysis details, model parameters and sensitivity analyses details.
**Appendix S5.** Description of interventions.
**Appendix S6.** Network meta‐analysis results for mechanistic treatment classification.
**Appendix S7.** Risk of bias assessments for included studies.
**Appendix S8.** Narrative results for all outcomes.
**Appendix S9.** Network plots and all relative effects from network meta‐analysis models of pharmacotherapies on each outcome.

## Data Availability

Data sharing not applicable to this article as no datasets were generated or analysed during the current study.

## References

[1] United Nations: Office on Drugs and Crime [Internet] . [cited 2025 Sep 19]. World Drug Report 2025 ‐ Key findings. Available from: //www.unodc.org/unodc/en/data-and-analysis/world-drug-report-2025-key-findings.html

[2] Schwartz EKC , Wolkowicz NR , De Aquino JP , MacLean RR , Sofuoglu M . Cocaine Use Disorder (CUD): Current clinical perspectives. Subst Abuse Rehabil. 2022;13:25–46. 10.2147/SAR.S337338 PMC9451050 36093428

[3] Hasin DS , O'Brien CP , Auriacombe M , Borges G , Bucholz K , Budney A , et al. DSM‐5 Criteria for Substance Use Disorders: Recommendations and Rationale. Am J Psychiatry. 2013 Aug 1;170(8):834–851. 10.1176/appi.ajp.2013.12060782 PMC3767415 23903334

[4] O'Brien MS , Anthony JC . Risk of Becoming Cocaine Dependent: Epidemiological Estimates for the United States, 2000–2001. Neuropsychopharmacol. 2005 May;30(5):1006–1018. 10.1038/sj.npp.1300681 15785780

[5] Substance Abuse and Mental Health Services Administration . Key Substance Use and Mental Health Indicators in the United States: Results from the 2019 National Survey on Drug use and Health [Internet]. Rockville, MD: Center for Behavioral Health Statistics and Quality, Substance Abuse and Mental Health Administration; 2020. Report Nos: PEP20‐07‐01–001. Available from: https://www.samhsa.gov/data/sites/default/files/reports/rpt29393/2019NSDUHFFRPDFWHTML/2019NSDUHFFR1PDFW090120.pdf

[6] Stankowski RV , Kloner RA , Rezkalla SH . Cardiovascular consequences of cocaine use. Trends Cardiovasc Med. 2015 Aug 1;25(6):517–526. 10.1016/j.tcm.2014.12.013 25657055

[7] Jovanovski D , Erb S , Zakzanis KK . Neurocognitive deficits in cocaine users: a quantitative review of the evidence. J Clin Exp Neuropsychol. 2005 Feb;27(2):189–204. 10.1080/13803390490515694 15903150

[8] Farrell M , Martin NK , Stockings E , Bórquez A , Cepeda JA , Degenhardt L , et al. Responding to global stimulant use: challenges and opportunities. The Lancet. 2019 Nov 2;394(10209):1652–1667. 10.1016/S0140-6736(19)32230-5 PMC6924572 31668409

[9] Daldegan‐Bueno D , Fischer B . The association between cocaine product use and violence outcomes in Brazil: A comprehensive, systematized review. Aggression and Violent Behavior. 2024;74:101891. 10.1016/j.avb.2023.101891

[10] Cocaine – the current situation in Europe (European Drug Report 2024) www.euda.europa.eu [Internet]. [cited 2025 Sep 19]. Available from: https://www.euda.europa.eu/publications/european-drug-report/2024/cocaine_en#level-5

[11] Bentzley BS , Han SS , Neuner S , Humphreys K , Kampman KM , Halpern CH . Comparison of treatments for cocaine use disorder among adults: a systematic review and meta‐analysis. JAMA Netw Open. 2021;4(5):e218049. 10.1001/jamanetworkopen.2021.8049 PMC8105751 33961037

[12] Rösner S , Hackl‐Herrwerth A , Leucht S , Lehert P , Vecchi S , Soyka M . Acamprosate for alcohol dependence. Cochrane Database Syst Rev. 2010;(9):CD004332. 10.1002/14651858.CD004332.pub2 PMC12147086 20824837

[13] Rösner S , Hackl‐Herrwerth A , Leucht S , Vecchi S , Srisurapanont M , Soyka M . Opioid antagonists for alcohol dependence. Cochrane Library. 2010;(12):CD001867. 10.1002/14651858.CD001867.pub3. PMC13590218 21154349

[14] Mattick RP , Breen C , Kimber J , Davoli M . Methadone maintenance therapy versus no opioid replacement therapy for opioid dependence. Cochrane Database Syst Rev. https://www.cochranelibrary.com/cdsr/doi/10.1002/14651858.CD002209.pub2/full. 2009;3:CD002209. 10.1002/14651858.CD002209.pub2 PMC7097731 19588333

[15] Chan B , Kondo K , Freeman M , Ayers C , Montgomery J , Kansagara D . Pharmacotherapy for Cocaine use disorder‐a systematic review and meta‐analysis. J Gen Intern Med. 2019;34(12):2858–2873. 10.1007/s11606-019-05074-8 PMC6854210 31183685

[16] Traccis F , Minozzi S , Trogu E , Vacca R , Vecchi S , Pani PP , et al. Disulfiram for the treatment of cocaine dependence. Cochrane Database Syst Rev. 2024;1(1):CD007024. 10.1002/14651858.CD007024.pub3 PMC10767770 38180268

[17] Hutton B , Salanti G , Caldwell DM , Chaimani A , Schmid CH , Cameron C , et al. The PRISMA Extension Statement for Reporting of Systematic Reviews Incorporating Network Meta‐analyses of Health Care Interventions: Checklist and Explanations. Ann Intern Med. 2015 Jun 2;162(11):777–784. 10.7326/M14-2385 26030634

[18] Castells X , Cunill R , Pérez‐Mañá C , Vidal X , Capellà D . Psychostimulant drugs for cocaine dependence. Cochrane Database Syst Rev. 2016;9(9):CD007380. 10.1002/14651858.CD007380.pub4 PMC6457633 27670244

[19] Chan B , Freeman M , Ayers C , Korthuis PT , Paynter R , Kondo K , et al. A systematic review and meta‐analysis of medications for stimulant use disorders in patients with co‐occurring opioid use disorders. Drug Alcohol Depend. 2020;216:108193. 10.1016/j.drugalcdep.2020.108193 PMC8562993 32861136

[20] Lee‐Cheong S , Ludgate SA , Epp TCM , Schütz CG . The effectiveness of oxytocin in the treatment of stimulant use disorders: a systematic review. Behav Pharmacol. 2023 Oct 1;34(7):381–392. 10.1097/FBP.0000000000000744 PMID: PubMed PMID: 37462158. 37462158

[21] Minozzi S , Cinquini M , Amato L , Davoli M , Farrell MF , Pani PP , et al. Anticonvulsants for cocaine dependence. Cochrane Database Syst Rev. 2015 Apr 17;2015(4):CD006754. 10.1002/14651858.CD006754.pub4 PMC8812341 25882271

[22] Tardelli VS , Bisaga A , Arcadepani FB , Gerra G , Levin FR , Fidalgo TM . Prescription psychostimulants for the treatment of stimulant use disorder: a systematic review and meta‐analysis. Psychopharmacology (Berl). 2020 Aug;237(8):2233–2255. 10.1007/s00213-020-05563-3 PMID: PubMed PMID: 32601988. 32601988

[23] Dias SW , Sutton A , Caldwell D , Lu G , Ades A . NICE DSU Technical Support Document 4: Inconsistency in the networks of evidence based on randomised controlled trials [Internet]. 2014. Available from: https://www.sheffield.ac.uk/media/34181/download?attachment 27466656

[24] Sterne JA , Savović J , Page MJ , Elbers RG , Blencowe NS , Boutron I , et al. RoB 2: A revised tool for assessing risk of bias in randomised trials. BMJ. 2019;366:l4898. 10.1136/bmj.l4898. 31462531

[25] Salanti G . Indirect and mixed‐treatment comparison, network, or multiple‐treatments meta‐analysis: many names, many benefits, many concerns for the next generation evidence synthesis tool. Res Synth Methods. 2012;3(2):80–97. 10.1002/jrsm.1037 26062083

[26] Phillippo DM , Perren SJ . multinma: Bayesian Network Meta‐Analysis of Individual and Aggregate Data.

[27] R Core Team . R: A Language and Environment for Statistical Computing [Internet]. Vienna, Austria; 2020. Available from: https://www.R-project.org/

[28] Nikolakopoulou A , Higgins JP , Papakonstantinou T , Chaimani A , Del Giovane C , Egger M , et al. CINeMA: An approach for assessing confidence in the results of a network meta‐analysis. PLoS Med. 2020;17(4):e1003082. 10.1371/journal.pmed.1003082 PMC7122720 32243458

[29] Covi L , Hess JM , Kreiter NA , Haertzen CA . Effects of combined fluoxetine and counseling in the outpatient treatment of cocaine abusers. Am J Drug Alcohol Abuse. 1995 Aug;21(3):327–344. Located at: S1 MEDLINE; 7484983. 10.3109/00952999509002701 7484983

[30] Epstein DH , Schmittner J , Umbricht A , Schroeder JR , Moolchan ET , Preston KL . Promoting abstinence from cocaine and heroin with a methadone dose increase and a novel contingency. Drug Alcohol Depend. 2009;101(1–2):92–100. Located at: Embase; 50367979. 10.1016/j.drugalcdep.2008.11.006 PMC2943844 19101098

[31] Grabowski J , Rhoades H , Silverman P , Schmitz JM , Stotts A , Creson D , et al. Risperidone for the treatment of cocaine dependence: Randomized, double‐ blind trial. J Clin Psychopharmacol. 2000;20(3):305–310. Located at: Embase; 30318358. 10.1097/00004714-200006000-00003 10831016

[32] Harris DS , Batki SL , Berger SP . Fluoxetine attenuates adrenocortical but not subjective responses to cocaine cues. Am J Drug Alcohol Abuse. 2004;30(4):765–782. Located at: Embase; 39578303. 10.1081/ADA-200037542 15624548

[33] Moeller FG , Schmitz JM , Steinberg JL , Green CM , Reist C , Lai LY , et al. Citalopram combined with behavioral therapy reduces cocaine use: a double‐blind, placebo‐controlled trial. Am J Drug Alcohol Abuse. 2007;33(3):367–378. Located at: S1 MEDLINE; 17613964. 10.1080/00952990701313686 17613964

[34] Montoya ID , Gorelick DA , Preston KL , Schroeder JR , Umbricht A , Cheskin LJ , et al. Randomized trial of buprenorphine for treatment of concurrent opiate and cocaine dependence. Clin Pharmacol Ther. 2004;75(1):34–48. 10.1016/j.clpt.2003.09.004 PMC2633656 14749690

[35] Oliveto A , Poling J , Sevarino KA , Gonsai KR , McCance‐Katz EF , Stine SM , et al. Efficacy of dose and contingency management procedures in LAAM‐maintained cocaine‐dependent patients. Drug Alcohol Depend. 2005;79(2):157–165. 10.1016/j.drugalcdep.2005.01.007 16002025

[36] Schmitz JM , Lindsay JA , Stotts AL , Green CE , Moeller FG . Contingency management and levodopa‐carbidopa for cocaine treatment: A comparison of three behavioral targets. Exp Clin Psychopharmacol. 2010;18(3):238–244. Located at: Embase; 359070231. 10.1037/a0019195 PMC3164487 20545388

[37] Delucchi KL , Batki SL , Moon J , Jacob P 3rd , Jones RT . Urine toxicology samples in cocaine treatment trials: How many need to be tested? J Addict Dis. 2002;21(2):17–26. 10.1300/J069v21n02_02 11916369

[38] Renshaw PF , Daniels S , Lundahl LH , Rogers V , Lukas SE . Short‐term treatment with citicoline (CDP‐choline) attenuates some measures of craving in cocaine‐dependent subjects: A preliminary report. Psychopharmacology (Berl). 1999;142(2):132–138. Located at: Embase; 29190847. 10.1007/s002130050871 10102764

[39] Schmitz JM , Rhoades HM , Elk R , Creson D , Hussein I , Grabowski J . Medication take‐home doses and contingency management. Exp Clin Psychopharmacol. 1998 May;6(2):162–168. Located at: S1 MEDLINE; 9608348. 10.1037//1064-1297.6.2.162 9608348

[40] Venneman S , Leuchter A , Bartzokis G , Beckson M , Simon SL , Schaefer M , et al. Variation in neurophysiological function and evidence of quantitative electroencephalogram discordance: Predicting cocaine‐dependent treatment attrition. J Neuropsychiatry Clin Neurosci. 2006;18(2):208–216. 10.1176/jnp.2006.18.2.208 16720798

[41] Afshar M , Knapp CM , Sarid‐Segal O , Devine E , Colaneri LS , Tozier L , et al. The efficacy of mirtazapine in the treatment of cocaine dependence with comorbid depression. Am J Drug Alcohol Abuse. 2012;38(2):181–186. Located at: Embase; 364342347. 10.3109/00952990.2011.644002 PMC5377928 22221171

[42] Alterman AI , Droba M , Antelo RE , Cornish JW , Sweeney KK , Parikh GA , et al. Amantadine may facilitate detoxification of cocaine addicts. Drug Alcohol Depend. 1992;31(1):19–29. Located at: Embase; 22320405. 10.1016/0376-8716(92)90004-V 1330470

[43] Anderson AL , Reid MS , Li S‐H , Holmes T , Shemanski L , Slee A , et al. Modafinil for the treatment of cocaine dependence. Drug Alcohol Depend. 2009;104(1–2):133–139. Located at: Embase; 50561360. 10.1016/j.drugalcdep.2009.04.015 PMC2818032 19560290

[44] Baldacara L , Diniz TA , Parreira BL , Milhomem JJ , de Almeida LJC , Fernandes CC . Could disulfiram be a new treatment for crack cocaine dependence? A pilot study. Revista Brasileira de Psiquiatria. 2013;35(1):97–98 Located at: Embase; 368725752. 10.1016/j.rbp.2012.10.006 23567610

[45] Baldacara L , Cogo‐Moreira H , Parreira BL , Diniz TA , Milhomem JJ , Fernandes CC , et al. Efficacy of topiramate in the treatment of crack cocaine dependence: A double‐blind, randomized, placebo‐controlled trial. J Clin Psychiatry. 2016;77(3):398–406. Located at: Embase; 609940086. 10.4088/JCP.14m09377 27046312

[46] Batki SL , Washburn AM , Delucchi K , Jones RT . A controlled trial of fluoxetine in crack cocaine dependence. Drug Alcohol Depend. 1996;41(2):137–142. Located at: Embase; 26183905. 10.1016/0376-8716(96)01233-1 8809502

[47] Becker JE , Price JL , Leonard D , Suris A , Kandil E , Shaw M , et al. The efficacy of lidocaine in disrupting cocaine cue‐induced memory reconsolidation. Drug Alcohol Depend. 2020;212:108062. 10.1016/j.drugalcdep.2020.108062 32480252

[48] Berger SP , Winhusen TM , Somoza EC , Harrer JM , Mezinskis JP , Leiderman DB , et al. A medication screening trial evaluation of reserpine, gabapentin and lamotrigine pharmacotherapy of cocaine dependence. Addiction. 2005;100(SUPPL. 1):58–67. Located at: Embase; 40363425. 10.1111/j.1360-0443.2005.00983.x 15730350

[49] Bisaga A , Aharonovich E , Garawi F , Levin FR , Rubin E , Raby WN , et al. A randomized placebo‐controlled trial of gabapentin for cocaine dependence. Drug Alcohol Depend. 2006;81(3):267–274. Located at: Embase; 43152453. 10.1016/j.drugalcdep.2005.07.009 16169160

[50] Bisaga A , Aharonovich E , Garawi F , Levin FR , Rubin E , Raby WN , et al. Utility of lead‐in period in cocaine dependence pharmacotherapy trials. Drug Alcohol Depend. 2005;77(1):7–11. 10.1016/j.drugalcdep.2004.06.007 15607836

[51] Bisaga A , Aharonovich E , Cheng WY , Levin FR , Mariani JJ , Raby WN , et al. A placebo‐controlled trial of memantine for cocaine dependence with high‐value voucher incentives during a pre‐randomization lead‐in period. Drug Alcohol Depend. 2010;111(1–2):97–104. Located at: Embase; 50926174. 10.1016/j.drugalcdep.2010.04.006 PMC2930076 20537812

[52] Blevins D , Seneviratne C , Wang XQ , Johnson BA , Ait‐Daoud N . A randomized, double‐blind, placebo‐controlled trial of ondansetron for the treatment of cocaine use disorder with post hoc pharmacogenetic analysis. Drug Alcohol Depend. 2019;228:109074. Located at: S1 MEDLINE; 34600264. 10.1016/j.drugalcdep.2021.109074 PMC8595865 34600264

[53] Brady KT , Sonne SC , Malcolm RJ , Randall CL , Dansky BS , Simpson K , et al. Carbamazepine in the treatment of cocaine dependence: Subtyping by affective disorder. Exp Clin Psychopharmacol. 2002;10(3):276–285. 10.1037//1064-1297.10.3.276 12233988

[54] Brodie JD , Case BG , Figueroa E , Dewey SL , Robinson JA , Wanderling JA , et al. Randomized, double‐blind, placebo‐controlled trial of vigabatrin for the treatment of cocaine dependence in Mexican parolees. Am J Psychiatry. 2009;166(11):1269–1277. 10.1176/appi.ajp.2009.08121811 19651710

[55] Campbell J , Nickel EJ , Penick EC , Wallace D , Gabrielli WF , Rowe C , et al. Comparison of desipramine or carbamazepine to placebo for crack cocaine‐dependent patients. The American Journal on Addictions. 2003;12(2):122–136. Located at: APA PsycInfo <2002 to 2003>. 10.1080/10550490390201380 12746087

[56] Campbell JL , Thomas HM , Gabrielli W , Liskow BI , Powell BJ . Impact of desipramine or carbamazepine on patient retention in outpatient cocaine treatment: Preliminary findings. J Addict Dis. 1994;13(4):191–199 Located at: Embase; 25072088. 10.1300/J069v13n04_07 7734469

[57] Carpenter KM , Choi CJ , Basaraba C , Pavlicova M , Brooks DJ , Brezing CA , et al. Mixed amphetamine salts‐extended release (MAS‐ER) as a behavioral treatment augmentation strategy for cocaine use disorder: A randomized clinical trial. Exp Clin Psychopharmacol. 2024 Feb;32(1):112–127. Located at: S1 MEDLINE; 37732961. 10.1037/pha0000676 PMC10872820 37732961

[58] Carroll KM , Rounsaville BJ , Gordon LT , Nich C , Jatlow P , Bisighini RM , et al. Psychotherapy and pharmacotherapy for ambulatory cocaine abusers. Arch Gen Psychiatry. 1994;51(3):177–187. 10.1001/archpsyc.1994.03950030013002 8122955

[59] Carroll KM , Rounsaville BJ , Nich C , Gordon LT , Wirtz PW , Gawin F . One‐year follow‐up of psychotherapy and pharmacotherapy for cocaine dependence. Delayed emergence of psychotherapy effects. Arch Gen Psychiatry. 1994 Dec;51(12):989–997. Located at: S1 MEDLINE; 7979888. 10.1001/archpsyc.1994.03950120061010 7979888

[60] Carroll KM , Nich C , Rounsaville BJ . Differential symptom reduction in depressed cocaine abusers treated with psychotherapy and pharmacotherapy. J Nerv Ment Dis. 1995;183(4):251–259. 10.1097/00005053-199504000-00012 7714514

[61] Carroll KM , Fenton LR , Ball SA , Nich C , Frankforter TL , Shi J , et al. Efficacy of Disulfiram and Cognitive Behavior Therapy in Cocaine‐Dependent Outpatients: A Randomized Placebo‐Controlled Trial. Arch Gen Psychiatry. 2004;61(3):264–272. Located at: Embase; 38295828. 10.1001/archpsyc.61.3.264 PMC3675448 14993114

[62] Carroll KM , Nich C , Shi JM , Eagan D , Ball SA . Efficacy of disulfiram and Twelve Step Facilitation in cocaine‐dependent individuals maintained on methadone: A randomized placebo‐controlled trial. Drug Alcohol Depend. 2012;126(1–2):224–231. Located at: Embase; 52054760. 10.1016/j.drugalcdep.2012.05.019 PMC3461119 22695473

[63] Carroll KM , Nich C , Petry NM , Eagan DA , Shi JM , Ball SA . A randomized factorial trial of disulfiram and contingency management to enhance cognitive behavioral therapy for cocaine dependence. Drug Alcohol Depend. 2016;160 ((Carroll, Nich, Eagan, Shi, Ball) Yale University School of Medicine, Department of Psychiatry, United States):135–142. 10.1016/j.drugalcdep.2015.12.036 PMC4767616 26817621

[64] DeVito EE , Dong G , Kober H , Xu J , Carroll KM , Potenza MN . Functional neural changes following behavioral therapies and disulfiram for cocaine dependence. Psychol Addict Behav. 2017;31(5):534–547. 10.1037/adb0000298 PMC5548432 28714728

[65] Carroll KM , Nich C , DeVito EE , Shi JM , Sofuoglu M . Galantamine and Computerized Cognitive Behavioral Therapy for cocaine dependence: A randomized clinical trial. J Clin Psychiatry. 2018;79(1):17m11669. 10.4088/JCP.17m11669 PMC5866530 29286595

[66] McCurdy LY , DeVito EE , Loya JM , Nich C , Zhai ZW , Kiluk BD , et al. Structural brain changes associated with cocaine use and digital cognitive behavioral therapy in cocaine use disorder treatment. Drug Alcohol Depend Rep. 2024;11:100246 Located at: Embase [“Drug literature “.ec.]; 2032741793. 10.1016/j.dadr.2024.100246 PMC11222934 38966567

[67] Ciraulo DA , Sarid‐Segal O , Knapp CM , Ciraulo AM , Locastro J , Bloch DA , et al. Efficacy screening trials of paroxetine, pentoxifylline, riluzole, pramipexole and venlafaxine in cocaine dependence. Addiction. 2005;100(SUPPL. 1):12–22. Located at: Embase; 40363421. 10.1111/j.1360-0443.2005.00985.x 15730346

[68] Streeter CC , Hennen J , Ke Y , Jensen JE , Sarid‐Segal O , Nassar LE , et al. Prefrontal GABA levels in cocaine‐dependent subjects increase with pramipexole and venlafaxine treatment. Psychopharmacology (Berl). 2005 Nov;182(4):516–526. Located at: S1 MEDLINE; 16075286. 10.1007/s00213-005-0121-5 16075286

[69] Ciraulo DA , Knapp C , Rotrosen J , Sarid‐Segal O , Ciraulo AM , LoCastro J , et al. Nefazodone treatment of cocaine dependence with comorbid depressive symptoms. Addiction. 2005;100(SUPPL. 1):23–31. Located at: Embase; 40363422. 10.1111/j.1360-0443.2005.00984.x 15730347

[70] Cornish JW , Maany I , Fudala PJ , Neal S , Poole SA , Volpicelli P , et al. Carbamazepine treatment for cocaine dependence. Drug Alcohol Depend. 1995;38(3):221–227. Located at: Embase; 25198288. 10.1016/0376-8716(95)01102-5 7555622

[71] Cornish JW , Maany I , Fudala PJ , Ehrman RN , Robbins SJ , O'Brien CP . A randomized, double‐blind, placebo‐controlled study of ritanserin pharmacotherapy for cocaine dependence. Drug Alcohol Depend. 2001;61(2):183–189. Located at: Embase; 32008532. 10.1016/S0376-8716(00)00140-X 11137283

[72] Ehrman RN , Robbins SJ , Cornish JW , Childress AR , O'Brien CP . Failure of ritanserin to block cocaine cue reactivity in humans. Drug Alcohol Depend. 1996;42(3):167–174. Located at: Ovid MEDLINE(R) <1996 to 2002>. 10.1016/s0376-8716(96)01278-1 8912799

[73] Dackis CA , Kampman KM , Lynch KG , Pettinati HM , O'Brien CP . A double‐blind, placebo‐controlled trial of modafinil for cocaine dependence. Neuropsychopharmacology. 2005;30(1):205–211. Located at: Embase; 40024415. 10.1038/sj.npp.1300600 15525998

[74] Dackis CA , Kampman KM , Lynch KG , Plebani JG , Pettinati HM , Sparkman T , et al. A double‐blind, placebo‐controlled trial of modafinil for cocaine dependence. J Subst Abuse Treat. 2012;43(3):303–312. Located at: Embase; 51882855. 10.1016/j.jsat.2011.12.014 PMC3378797 22377391

[75] Dakwar E , Nunes EV , Hart CL , Foltin RW , Mathew SJ , Carpenter KM , et al. A single ketamine infusion combined with mindfulness‐based behavioral modification to treat cocaine dependence: A randomized clinical trial. Am J Psychiatry. 176(11):923–930. 10.1176/appi.ajp.2019.18101123 31230464

[76] DeVito EE , Carroll KM , Babuscio T , Nich C , Sofuoglu M . Randomized placebo‐controlled trial of galantamine in individuals with cocaine use disorder. J Subst Abuse Treat. 2019;107:29–37. 10.1016/j.jsat.2019.08.009 PMC6918721 31757262

[77] DeVito EE , Poling J , Babuscio T , Nich C , Carroll KM , Sofuoglu M . Modafinil does not reduce cocaine use in methadone‐maintained individuals. Drug Alcohol Depend Rep. 2022;2:100032. 10.1016/j.dadr.2022.100032 PMC9615926 36310662

[78] Dieckmann LHJ , Ramos AC , Silva EA , Justo LP , Sabioni P , Frade IF , et al. Effects of biperiden on the treatment of cocaine/crack addiction: A randomised, double‐blind, placebo‐controlled trial. Eur Neuropsychopharmacol. 2014;24(8):1196–1202. Located at: Embase; 373625588. 10.1016/j.euroneuro.2014.06.001 24974353

[79] Dursteler‐Macfarland KM , Farronato NS , Strasser J , Boss J , Kuntze MF , Petitjean SA , et al. A randomized, controlled, pilot trial of methylphenidate and cognitive‐behavioral group therapy for cocaine dependence in heroin prescription. J Clin Psychopharmacol. 2013;33(1):104–108. Located at: Embase; 52376611. 10.1097/JCP.0b013e31827bfff4 23277248

[80] Elkashef A , Fudala PJ , Gorgon L , Li S‐H , Kahn R , Chiang N , et al. Double‐blind, placebo‐controlled trial of selegiline transdermal system (STS) for the treatment of cocaine dependence. Drug Alcohol Depend. 2006;85(3):191–197. Located at: Embase; 44666279. 10.1016/j.drugalcdep.2006.04.010 16730924

[81] Gawin FH , Kleber HD , Byck R , Rounsaville BJ , Kosten TR , Jatlow PI , et al. Desipramine facilitation of initial cocaine abstinence. Archives of General Psychiatry. 1989;A.M.A. Archives of General Psychiatry. JAMA Psychiatry. 46(2):117–121. Located at: APA PsycInfo <1987 to 2001>. 10.1001/archpsyc.1989.01810020019004 2492422

[82] Kosten TR , Gawin FH , Kosten TA , Morgan C , Rounsaville BJ , Schottenfeld R , et al. Six‐month follow‐up of short‐term pharmacotherapy for cocaine dependence. Am J Addict. 1992;1(1):40–49. Located at: S1 APA PsycInfo <1987 to 2001>; 1994‐03259‐001. 10.3109/10550499208998339

[83] George TP , Chawarski MC , Pakes J , Carroll KM , Kosten TR , Schottenfeld RS . Disulfiram versus placebo for cocaine dependence in buprenorphine‐maintained subjects: a preliminary trial. Biol Psychiatry. 2000;47(12):1080–1086. Located at: Ovid MEDLINE(R) <1996 to 2002>. 10.1016/s0006-3223(99)00310-8 10862808

[84] Gilgun‐Sherki Y , Eliaz RE , McCann DJ , Loupe PS , Eyal E , Blatt K , et al. Placebo‐controlled evaluation of a bioengineered, cocaine‐metabolizing fusion protein, TV‐1380 (AlbuBChE), in the treatment of cocaine dependence. Drug Alcohol Depend. 2016;166:13–20. 10.1016/j.drugalcdep.2016.05.019 27394932

[85] Gonzalez G , Sevarino K , Sofuoglu M , Poling J , Oliveto A , Gonsai K , et al. Tiagabine increases cocaine‐free urines in cocaine‐dependent methadone‐treated patients: Results of a randomized pilot study. Addiction. 2003;98(11):1625–1632. Located at: Embase; 37373861. 10.1046/j.1360-0443.2003.00544.x 14616189

[86] Gorelick DA , Wilkins JN . Bromocriptine treatment for cocaine addiction: Association with plasma prolactin levels. Drug Alcohol Depend. 2006;81(2):189–195. Located at: Embase; 41821802. 10.1016/j.drugalcdep.2005.06.010 16051446

[87] Grabowski J , Rhoades H , Elk R , Schmitz J , Davis C , Creson D , et al. Fluoxetine is ineffective for treatment of cocaine dependence or concurrent opiate and cocaine dependence: two placebo‐controlled double‐blind trials. J Clin Psychopharmacol. 1995;15(3):163–174. Located at: Ovid MEDLINE(R) <1988 to 1995>. 10.1097/00004714-199506000-00004 7635993

[88] Grabowski J , Roache JD , Schmitz JM , Rhoades H , Creson D , Korszun A . Replacement medication for cocaine dependence: Methylphenidate. J Clin Psychopharmacol. 1997;17(6):485–488 Located at: Embase; 28389189. 10.1097/00004714-199712000-00008 9408812

[89] Grabowski J , Rhoades H , Schmitz J , Stotts A , Daruzska LA , Creson D , et al. Dextroamphetamine for cocaine‐dependence treatment: A double‐blind randomized clinical trial. J Clin Psychopharmacol. 2001;21(5):522–526. Located at: Embase; 32937435. 10.1097/00004714-200110000-00010 11593078

[90] Grabowski J , Rhoades H , Stotts A , Cowan K , Kopecky C , Dougherty A , et al. Agonist‐like or antagonist‐like treatment for cocaine dependence with methadone for heroin dependence: Two double‐blind randomized clinical trials. Neuropsychopharmacology. 2004;29(5):969–981. Located at: Embase; 38526797. 10.1038/sj.npp.1300392 15039761

[91] Halikas JA , Crosby RD , Pearson VL , Graves NM . A randomized double‐blind study of carbamazepine in the treatment of cocaine abuse. Clin Pharmacol Ther. 1997;62(1):89–105 Located at: Embase; 27327103. 10.1016/S0009-9236(97)90155-7 9246023

[92] Hall SM , Tunis S , Triffleman E , Banys P , Clark HW , Tusel D , et al. Continuity of care and desipramine in primary cocaine abusers. J Nerv Ment Dis. 1994 Oct;182(10):570–575. Located at: S1 MEDLINE; 7931205. 10.1097/00005053-199410000-00007 7931205

[93] Tunis SL , Delucchi KL , Hall SM . Assessing thoughts about cocaine and their relationship to short‐term treatment outcome. Exp Clin Psychopharmacol. 1994;2(2):184–193. Located at: S1 APA PsycInfo <1987 to 2001>; 1994–31213‐001. 10.1037/1064-1297.2.2.184

[94] Hamilton JD , Nguyen QX , Gerber RM , Rubio NB . Olanzapine in cocaine dependence: A double‐blind, placebo‐controlled trial. Am J Addict. 2009;18(1):48–52. Located at: Embase; 354201668. 10.1080/10550490802544318 19219665

[95] Handelsman L , Limpitlaw L , Williams D , Schmeidler J , Paris P , Stimmel B . Amantadine does not reduce cocaine use or craving in cocaine‐dependent methadone maintenance patients. Drug Alcohol Depend. 1995 Oct 1;39(3):173–180. 10.1016/0376-8716(95)01154-9 8556965

[96] Hersh D , Van Kirk JR , Kranzler HR . Naltrexone treatment of comorbid alcohol and cocaine use disorders. Psychopharmacology (Berl). 1998;139(1–2):44–52. Located at: Embase; 28416881. 10.1007/s002130050688 9768541

[97] Johnson BA , Chen YR , Swann AC , Schmitz J , Lesser J , Ruiz P , et al. Ritanserin in the treatment of cocaine dependence. Biol Psychiatry. 1997;42(10):932–940. Located at: Embase; 27486448. 10.1016/S0006-3223(96)00490-8 9359980

[98] Johnson BA , Roache JD , Ait‐Daoud N , Javors MA , Harrison JM , Elkashef A , et al. A preliminary randomized, double‐blind, placebo‐controlled study of the safety and efficacy of ondansetron in the treatment of cocaine dependence. Drug Alcohol Depend. 2006;84(3):256–263. Located at: Embase; 44287214. 10.1016/j.drugalcdep.2006.02.011 16631323

[99] Johnson BA , Ait‐Daoud N , Wang XQ , Penberthy JK , Javors MA , Seneviratne C , et al. Topiramate for the treatment of cocaine addiction: a randomized clinical trial. JAMA. Psychiatry. 2013;70(12):1338–1346. Located at: Ovid MEDLINE(R) <2013>. 10.1001/jamapsychiatry.2013.2295 24132249

[100] Blevins D , Wang XQ , Sharma S , Ait‐Daoud N . Impulsiveness as a predictor of topiramate response for cocaine use disorder. Am J Addict. 2019;28(2):71–76. 10.1111/ajad.12858 PMC8107800 30664303

[101] Johnson MW , Bruner NR , Johnson PS , Silverman K , Berry MS . Randomized Controlled Trial of D‐Cycloserine in Cocaine Dependence: Effects on Contingency Management and Cue‐Induced Cocaine Craving in a Naturalistic Setting. Exp Clin Psychopharmacol. 2020;28(2):157–168. Located at: Embase; 628872947. 10.1037/pha0000306 PMC6994347 31368770

[102] Jones HE , Johnson RE , Bigelow GE , Silverman K , Mudric T , Strain EC . Safety and efficacy of L‐tryptophan and behavioral incentives for treatment of cocaine dependence: A randomized clinical trial. Am J Addict. 2004;13(5):421–437. Located at: Embase; 39603288. 10.1080/10550490490512753 15764421

[103] Junior MSC , Bezerra AG , Curado DF , Gregório RP , Galduróz JCF . Preliminary investigation of the administration of biperiden to reduce relapses in individuals with cocaine/crack user disorder: A randomized controlled clinical trial. Pharmacol Biochem Behav. 2024;237:173725. 10.1016/j.pbb.2024.173725 38340989

[104] Kablinger AS , Lindner MA , Casso S , Hefti F , Demuth G , Fox BS , et al. Effects of the combination of metyrapone and oxazepam on cocaine craving and cocaine taking: A double‐blind, randomized, placebo‐controlled pilot study. J Psychopharmacol. 2012;26(7):973–981. Located at: Embase; 365106486. 10.1177/0269881111430745 22236504

[105] Kahn R , Biswas K , Childress A‐R , Shoptaw S , Fudala PJ , Gorgon L , et al. Multi‐center trial of baclofen for abstinence initiation in severe cocaine‐dependent individuals. Drug Alcohol Depend. 2009;103(1–2):59–64. Located at: Embase; 354570185. 10.1016/j.drugalcdep.2009.03.011 PMC2774896 19414226

[106] Kampman K , Volpicelli JR , Alterman A , Cornish J , Weinrieb R , Epperson L , et al. Amantadine in the early treatment of cocaine dependence: A double‐blind, placebo‐controlled trial. Drug Alcohol Depend. 1996;41(1):25–33. Located at: Embase; 26185292. 10.1016/0376-8716(96)01225-2 8793307

[107] Kampman KM , Volpicelli JR , Alterman AI , Cornish J , O'Brien CP . Amantadine in the treatment of cocaine‐dependent patients with severe withdrawal symptoms. Am J Psychiatry. 2000;157(12):2052–2054. 10.1176/appi.ajp.157.12.2052 11097979

[108] Kampman KM , Volpicelli JR , Mulvaney F , Alterman AI , Cornish J , Gariti P , et al. Effectiveness of propranolol for cocaine dependence treatment may depend on cocaine withdrawal symptom severity. Drug Alcohol Depend. 2001;63(1):69–78. Located at: Ovid MEDLINE(R) <1996 to 2002>. 10.1016/s0376-8716(00)00193-9 11297832

[109] Kampman KM , Pettinati H , Lynch KG , Sparkman T , O'Brien CP . A pilot trial of olanzapine for the treatment of cocaine dependence. Drug Alcohol Depend. 2003 Jun 5;70(3):265–273. 10.1016/S0376-8716(03)00009-7 12757964

[110] Kampman K , Majewska MD , Tourian K , Dackis C , Cornish J , Poole S , et al. A pilot trial of piracetam and *ginkgo biloba* for the treatment of cocaine dependence. Addict Behav. 2003;28(3):437–448. 10.1016/s0306-4603(02)00226-5 12628617

[111] Kampman KM , Pettinati H , Lynch KG , Dackis C , Sparkman T , Weigley C , et al. A pilot trial of topiramate for the treatment of cocaine dependence. Drug Alcohol Depend. 2004;75(3):233–240. Located at: Embase; 38993293. 10.1016/j.drugalcdep.2004.03.008 15283944

[112] Kampman KM , Dackis C , Lynch KG , Pettinati H , Tirado C , Gariti P , et al. A double‐blind, placebo‐controlled trial of amantadine, propranolol, and their combination for the treatment of cocaine dependence in patients with severe cocaine withdrawal symptoms. Drug Alcohol Depend. 2006;85(2):129–137. Located at: Embase; 44441667. 10.1016/j.drugalcdep.2006.04.002 16697124

[113] Fairbairn CE , Dundon WD , Xie H , Plebani JG , Kampman KM , Lynch KG . Study blinding and correlations between perceived group assignment and outcome in a cocaine pharmacotherapy trial. Am J Addict. 2008 Sep;17(5):387–391. Located at: S1 MEDLINE; 18770080. 10.1080/10550490802266078 PMC3172694 18770080

[114] Kampman KM , Dackis C , Pettinati HM , Lynch KG , Sparkman T , O'Brien CP . A double‐blind, placebo‐controlled pilot trial of acamprosate for the treatment of cocaine dependence. Addict Behav. 2011;36(3):217–221. Located at: Embase; 51168155. 10.1016/j.addbeh.2010.11.003 PMC3018663 21112155

[115] Kampman KM , Pettinati HM , Lynch KG , Spratt K , Wierzbicki MR , O'Brien CP . A double‐blind, placebo‐controlled trial of topiramate for the treatment of comorbid cocaine and alcohol dependence. Drug Alcohol Depend. 2013;133(1):94–99 Located at: Embase; 52651209. 10.1016/j.drugalcdep.2013.05.026 PMC3786029 23810644

[116] Kampman KM , Lynch KG , Pettinati HM , Spratt K , Wierzbicki MR , Dackis C , et al. A double blind, placebo controlled trial of modafinil for the treatment of cocaine dependence without comorbid alcohol dependence. Drug Alcohol Depend. 2015;155:105–110. 10.1016/j.drugalcdep.2015.08.005 PMC4582003 26320827

[117] Karila L , Leroy C , Dubol M , Trichard C , Mabondo A , Marill C , et al. Dopamine transporter correlates and occupancy by modafinil in cocaine‐dependent patients: A controlled study with high‐resolution PET and [11 C]‐PE2I. Neuropsychopharmacology. 2016;41(9):2294–2302. Located at: Embase; 609137618. 10.1038/npp.2016.28 PMC4946060 26892922

[118] Kennedy AP , Gross RE , Whitfield N , Drexler KP , Kilts CD . A controlled trial of the adjunct use of D‐cycloserine to facilitate cognitive behavioral therapy outcomes in a cocaine‐dependent population. Addict Behav. 2012;37(8):900–907. 10.1016/j.addbeh.2012.03.008 PMC3366369 22578380

[119] Kolar AF , Brown BS , Weddington WW , Haertzen CC , Michaelson BS , Jaffe JH . Treatment of cocaine dependence in methadone maintenance clients: A pilot study comparing the efficacy of desipramine and amantadine. Int J Addict. 1992;27(7):849–868. Located at: Embase; 22190311. 10.3109/10826089209068770 1319961

[120] Kosten TR , Morgan CM , Falcione J , Schottenfeld RS . Pharmacotherapy for cocaine‐abusing methadone‐maintained patients using amantadine or desipramine. Archives of General Psychiatry. 1992;A.M.A. Archives of General Psychiatry. JAMA Psychiatry. 49(11):894–898. Located at: APA PsycInfo <1987 to 2001>. 10.1001/archpsyc.1992.01820110058009 1444728

[121] Leal J , Ziedonis D , Kosten T . Antisocial personality disorder as a prognostic factor for pharmacotherapy of cocaine dependence. Drug Alcohol Depend. 1994 Mar;35(1):31–35. Located at: S1 MEDLINE; 8082553. 10.1016/0376-8716(94)90107-4 8082553

[122] Ziedonis DM , Kosten TR . Depression as a prognostic factor for pharmacological treatment of cocaine dependence. Psychopharmacol Bull. 1991;27(3):337–343. PMID: Located at: S1 MEDLINE; 1775608. 1775608

[123] Ziedonis DM , Kosten TR . Pharmacotherapy Improves Treatment Outcome in Depressed Cocaine Addicts. J Psychoactive Drugs. 1991 Oct 1;23(4):417–425. 10.1080/02791072.1991.10471612 PMID: PubMed PMID: 1813613. 1813613

[124] Kosten TR , Oliveto A , Sevarino KA , Gonsai K , Feingold A . Ketoconazole increases cocaine and opioid use in methadone maintained patients. Drug Alcohol Depend. 2002;66(2):173–180. Located at: Ovid MEDLINE(R) <1996 to 2002>. 10.1016/s0376-8716(01)00198-3 11906804

[125] Kosten T , Oliveto A , Feingold A , Poling J , Sevarino K , McCance‐Katz E , et al. Desipramine and contingency management for cocaine and opiate dependence in buprenorphine maintained patients. Drug Alcohol Depend. 2003;70(3):315–325. Located at: Ovid MEDLINE(R) <2003 to 2004>. 10.1016/s0376-8716(03)00032-2 12757969

[126] Gonzalez G , Feingold A , Oliveto A , Gonsai K , Kosten TR . Comorbid major depressive disorder as a prognostic factor in cocaine‐abusing buprenorphine‐maintained patients treated with desipramine and contingency management. Am J Drug Alcohol Abuse. 2003 Aug;29(3):497–514. Located at: S1 MEDLINE; 14510037. 10.1081/ada-120023455 14510037

[127] Kosten T , Poling J , Oliveto A . Effects of reducing contingency management values on heroin and cocaine use for buprenorphine‐ and desipramine‐treated patients. Addiction. 2003 May;98(5):665–671. Located at: S1 MEDLINE; 12751984. 10.1046/j.1360-0443.2003.00380.x 12751984

[128] Sofuoglu M , Gonzalez G , Poling J , Kosten TR . Prediction of treatment outcome by baseline urine cocaine results and self‐reported cocaine use for cocaine and opioid dependence. Am J Drug Alcohol Abuse. 2003;29(4):713–727. Located at: S1 MEDLINE; 14713135. 10.1081/ada-120026256 14713135

[129] Kosten TR , Wu G , Huang W , Harding MJ , Hamon SC , Lappalainen J , et al. Pharmacogenetic randomized trial for cocaine abuse: Disulfiram and dopamine beta‐hydroxylase. Biol Psychiatry. 2013;73(3):219–224. Located at: Embase; 52167105. 10.1016/j.biopsych.2012.07.011 PMC3514624 22906516

[130] Kampangkaew JP , Spellicy CJ , Nielsen EM , Harding MJ , Ye A , Hamon SC , et al. Pharmacogenetic role of dopamine transporter (SLC6A3) variation on response to disulfiram treatment for cocaine addiction. Am J Addict 7 AD. 28(4):311–317. Located at: S1 MEDLINE; 31087723. 10.1111/ajad.12891 PMC6591035 31087723

[131] Nielsen DA , Harding MJ , Hamon SC , Huang W , Kosten TR . Modifying the role of serotonergic 5‐HTTLPR and TPH2 variants on disulfiram treatment of cocaine addiction: A preliminary study. Genes Brain Behav. 2012;11(8):1001–1008. 10.1111/j.1601-183X.2012.00839.x PMC3521860 22925276

[132] Shorter D , Nielsen DA , Huang W , Harding MJ , Hamon SC , Kosten TR . Pharmacogenetic randomized trial for cocaine abuse: Disulfiram and alpha1A‐adrenoceptor gene variation. Eur Neuropsychopharmacol. 2013 Nov;23(11):1401–1407. Located at: S1 MEDLINE; 23849431. 10.1016/j.euroneuro.2013.05.014 PMC3818518 23849431

[133] Spellicy CJ , Kosten TR , Hamon SC , Harding MJ , Nielsen DA . The MTHFR C677T variant is associated with responsiveness to Disulfiram treatment for Cocaine dependency. Front Psychiatry. 2012;3:109. 10.3389/fpsyt.2012.00109 PMC3544007 23335901

[134] Kosten TR , Domingo CB , Shorter D , Orson F , Green C , Somoza E , et al. Vaccine for cocaine dependence: A randomized double‐blind placebo‐controlled efficacy trial. Drug Alcohol Depend. 2014;140:42–47. 10.1016/j.drugalcdep.2014.04.003 PMC4073297 24793366

[135] Kranzler HR , Bauer LO , Hersh D , Klinghoffer V . Carbamazepine treatment of cocaine dependence: a placebo‐controlled trial. Drug Alcohol Depend. 1995;Comment in: Drug Alcohol Depend. 1995 Jun;38(3):201–202 PMID: 7555619 [https://www.ncbi.nlm.nih.gov/pubmed/7555619]38(3):203–11. Located at: Ovid MEDLINE(R) <1988 to 1995>. 10.1016/0376-8716(95)01100-d 7555620

[136] LaRowe SD , Kalivas PW , Nicholas JS , Randall PK , Mardikian PN , Malcolm RJ . A double‐blind placebo‐controlled trial of N‐acetylcysteine in the treatment of cocaine dependence. Am J Addict. 2013;22(5):443–452. Located at: Embase; 52592430. 10.1111/j.1521-0391.2013.12034.x PMC4348575 23952889

[137] Levin FR , McDowell D , Evans SM , Brooks D , Spano C , Nunes EV . Pergolide mesylate for cocaine abuse: a controlled preliminary trial. Am J Addict. 1999;8(2):120–127. Located at: S1 MEDLINE; 10365192. 10.1080/105504999305929 10365192

[138] Levin FR , Evans SM , Brooks DJ , Garawi F . Treatment of cocaine dependent treatment seekers with adult ADHD: Double‐blind comparison of methylphenidate and placebo. Drug Alcohol Depend. 2007;87(1):20–29. Located at: Embase; 46074379. 10.1016/j.drugalcdep.2006.07.004 16930863

[139] Aharonovich E , Garawi F , Bisaga A , Brooks D , Raby WN , Rubin E , et al. Concurrent cannabis use during treatment for comorbid ADHD and cocaine dependence: effects on outcome. Am J Drug Alcohol Abuse. 2006;32(4):629–635. 10.1080/00952990600919005 PMID: PubMed PMID: 17127551. 17127551

[140] Levin FR , Mariani JJ , Specker S , Mooney M , Mahony A , Brooks DJ , et al. Extended‐release mixed amphetamine salts vs placebo for comorbid adult attention‐deficit/hyperactivity disorder and cocaine use disorder a randomized clinical trial. JAMA Psychiatry. 2015;72(6):593–602. 10.1001/jamapsychiatry.2015.41 PMC4456227 25887096

[141] Blevins D , Choi CJ , Pavlicova M , Martinez D , Mariani JJ , Grabowski J , et al. Impulsiveness as a moderator of amphetamine treatment response for cocaine use disorder among ADHD patients. Drug Alcohol Depend. 2020 Aug;213:108082. Located at: S1 MEDLINE; 32485656. 10.1016/j.drugalcdep.2020.108082 PMC7371538 32485656

[142] Levin FR , Choi CJ , Pavlicova M , Mariani JJ , Mahony A , Brooks DJ , et al. How treatment improvement in ADHD and cocaine dependence are related to one another: A secondary analysis. Drug Alcohol Depend. 2018 Jul 1;188:135–140. 10.1016/j.drugalcdep.2018.03.043 PMC6158788 29775957

[143] Notzon DP , Mariani JJ , Pavlicova M , Glass A , Mahony AL , Brooks DJ , et al. Mixed‐amphetamine salts increase abstinence from marijuana in patients with co‐occurring attention‐deficit/hyperactivity disorder and cocaine dependence. Am J Addict. 12 AD. 25(8):666–672. Located at: S1 MEDLINE; 28051838. 10.1111/ajad.12467 PMC5435118 28051838

[144] Levin FR , Mariani JJ , Pavlicova M , Choi CJ , Mahony AL , Brooks DJ , et al. Extended release mixed amphetamine salts and topiramate for cocaine dependence: A randomized clinical replication trial with frequent users. Drug Alcohol Depend. 2020;206:107700. 10.1016/j.drugalcdep.2019.107700 PMC6980777 31753736

[145] Licata SC , Penetar DM , Ravichandran C , Rodolico J , Palmer C , Berko J , et al. Effects of daily treatment with citicoline: A double‐blind, placebo‐controlled study in cocaine‐dependent volunteers. J Addict Med. 2011;5(1):57–64. Located at: Embase; 361611249. 10.1097/ADM.0b013e3181d80c93 PMC3140015 21769048

[146] Bracken BK , Penetar DM , Rodolico J , Ryan ET , Lukas SE . Eight weeks of citicoline treatment does not perturb sleep/wake cycles in cocaine‐dependent adults. Pharmacol Biochem Behav. 2011 Jun;98(4):518–524. Located at: S1 MEDLINE; 21397626. 10.1016/j.pbb.2011.03.003 PMC3081995 21397626

[147] Ling W , Hillhouse MP , Saxon AJ , Mooney LJ , Thomas CM , Ang A , et al. Buprenorphine + naloxone plus naltrexone for the treatment of cocaine dependence: The Cocaine Use Reduction with Buprenorphine (CURB) study. Addiction. 2016; British Journal of Addiction;111(8):1416–1427. Located at: APA PsycInfo <2016>. 10.1111/add.13375 PMC4940267 26948856

[148] Nielsen DA , Walker R , Graham DP , Nielsen EM , Hamon SC , Hillhouse M , et al. Moderation of buprenorphine therapy for cocaine dependence efficacy by variation of the Prodynorphin gene. Eur J Clin Pharmacol. 2022 Jun;78(6):965–973. Located at: S1 MEDLINE; 35218405. 10.1007/s00228-022-03302-5 35218405

[149] Loebl T , Angarita GA , Pachas GN , Huang K‐L , Lee SH , Nino J , et al. A randomized, double‐blind, placebo‐controlled trial of long‐acting risperidone in cocaine‐dependent men. J Clin Psychiatry. 2008;69(3):480–486. Located at: Embase; 351549404. 10.4088/JCP.v69n0321 18294021

[150] Lynch KG , Plebani J , Spratt K , Morales M , Tamminga M , Feibush P , et al. Varenicline for the Treatment of Cocaine Dependence. J Addict Med. 2022 Mar;16(2):157–163. Located at: S1 MEDLINE; 33840773. 10.1097/ADM.0000000000000842 PMC8497642 33840773

[151] Malcolm R , Kajdasz DK , Herron J , Anton RF , Brady KT . A double‐blind, placebo‐controlled outpatient trial of pergolide for cocaine dependence. Drug Alcohol Depend. 2000;60(2):161–168. Located at: Ovid MEDLINE(R) <1996 to 2002>. 10.1016/s0376-8716(99)00151-9 10940543

[152] Malcolm R , Herron J , Sutherland SE , Brady KT . Adverse outcomes in a controlled trial of pergolide for cocaine dependence. J Addict Dis. 2001;20(1):81–92. Located at: Embase; 32247024. 10.1300/J069v20n01_08 11286433

[153] Malcolm R , LaRowe S , Cochran K , Moak D , Herron J , Brady K , et al. A controlled trial of amlodipine for cocaine dependence: A negative report. J Subst Abuse Treat. 2005;28(2):197–204. Located at: Embase; 40380928. 10.1016/j.jsat.2004.12.006 15780550

[154] Malcolm R , Liao J , Michel M , Cochran K , Pye W , Yeager D , et al. Amlodipine reduces blood pressure and headache frequency in cocaine‐dependent outpatients. J Psychoactive Drugs. 2002 Oct;34(4):415–419. Located at: S1 MEDLINE; 12562110. 10.1080/02791072.2002.10399983 12562110

[155] Turner TH , LaRowe S , Horner MD , Herron J , Malcolm R . Measures of cognitive functioning as predictors of treatment outcome for cocaine dependence. J Subst Abuse Treat. 2009 Dec;37(4):328–334. Located at: S1 MEDLINE; 19394790. 10.1016/j.jsat.2009.03.009 19394790

[156] Mancino MJ , McGaugh J , Chopra MP , Guise JB , Cargile C , Williams DK , et al. Clinical efficacy of sertraline alone and augmented with gabapentin in recently abstinent cocaine‐dependent patients with depressive symptoms. J Clin Psychopharmacol. 2014;34(2):234–239. Located at: Embase; 53009616. 10.1097/JCP.0000000000000062 PMC4068618 24525654

[157] Margolin A , Kosten TR , Kelly Avants S , Wilkins J , Ling W , Beckson M , et al. A multicenter trial of bupropion for cocaine dependence in methadone‐maintained patients. Drug Alcohol Depend. 1995;40(2):125–131 Located at: Embase; 26011975. 10.1016/0376-8716(95)01198-6 8745134

[158] Margolin A , Avants SK , Malison RT , Kosten TR . High‐ and low‐dose mazindol for cocaine dependence in methadone‐ maintained patients: A preliminary evaluation. Subst Abuse. 1997;18(3):125–131 Located at: Embase; 27413639. 10.1080/08897079709511358

[159] Mariani JJ , Pavlicova M , Bisaga A , Nunes EV , Brooks DJ , Levin FR . Extended‐release mixed amphetamine salts and topiramate for cocaine dependence: A randomized controlled trial. Biol Psychiatry. 2012;72(11):950–956. Located at: Embase; 52107895. 10.1016/j.biopsych.2012.05.032 PMC3648884 22795453

[160] Martell BA , Orson FM , Poling J , Mitchell E , Rossen RD , Gardner T , et al. Cocaine vaccine for the treatment of cocaine dependence in methadone‐maintained patients: A randomized, double‐blind, placebo‐controlled efficacy trial. Arch Gen Psychiatry. 2009;66(10):1116–1123. Located at: Embase; 355381338. 10.1001/archgenpsychiatry.2009.128 PMC2878137 19805702

[161] Kosten TR , Domingo CB , Hamon SC , Nielsen DA . DBH gene as predictor of response in a cocaine vaccine clinical trial. Neurosci Lett. 2013;541:29–33. 10.1016/j.neulet.2013.02.037 PMC3631430 23458673

[162] Nielsen DA , Hamon SC , Kosten TR . The kappa‐opioid receptor gene as a predictor of response in a cocaine vaccine clinical trial. Psychiatr Genet. 2013 Dec;23(6):225–232. Located at: S1 MEDLINE; 23995774. 10.1097/YPG.0000000000000008 PMC3885873 23995774

[163] McCann DJ , Chen HH , Devine EG , Gyaw S , Ramey T . Results of a randomized, double‐blind, placebo‐controlled trial of lorcaserin in cocaine use disorder. Drug Alcohol Depend. 2024;255:111063. 10.1016/j.drugalcdep.2023.111063 PMC10872513 38163425

[164] McDowell D , Nunes EV , Seracini AM , Rothenberg J , Vosburg SK , Ma GJ , et al. Desipramine treatment of cocaine‐dependent patients with depression: A placebo‐controlled trial. Drug Alcohol Depend. 2005;80(2):209–221. Located at: Embase; 41427635. 10.1016/j.drugalcdep.2005.03.026 15913920

[165] Moeller FG , Dougherty DM , Barratt ES , Schmitz JM , Swann AC , Grabowski J . The impact of impulsivity on cocaine use and retention in treatment. J Subst Abuse Treat. 2001 Dec;21(4):193–198. Located at: S1 MEDLINE; 11777668. 10.1016/s0740-5472(01)00202-1 11777668

[166] Green CE , Moeller FG , Schmitz JM , Lucke JF , Lane SD , Swann AC , et al. Evaluation of heterogeneity in pharmacotherapy trials for drug dependence: a Bayesian approach. Am J Drug Alcohol Abuse. 2009;35(2):95–102. Located at: S1 MEDLINE; 19322730. 10.1080/00952990802647503 PMC4429522 19322730

[167] Schmitz JM , Mooney ME , Green CE , Lane SD , Steinberg JL , Swann AC , et al. Baseline neurocognitive profiles differentiate abstainers and non‐abstainers in a cocaine clinical trial. J Addict Dis. 2009 Jul;28(3):250–257. Located at: S1 MEDLINE; 20155594. 10.1080/10550880903028502 PMC2822995 20155594

[168] Mongeau‐Perusse V , Brissette S , Bruneau J , Conrod P , Dubreucq S , Gazil G , et al. Cannabidiol as a treatment for craving and relapse in individuals with cocaine use disorder: a randomized placebo‐controlled trial. Addiction. 2021;116(9):2431–2442. Located at: Embase; 634060581. 10.1111/add.15417 PMC8451934 33464660

[169] Hebert FO , Mongeau‐Perusse V , Rizkallah E , Mahroug A , Bakouni H , Morissette F , et al. Absence of Evidence for Sustained Effects of Daily Cannabidiol Administration on Anandamide Plasma Concentration in Individuals with Cocaine Use Disorder: Exploratory Findings from a Randomized Controlled Trial. Cannabis Cannabinoid Res. 2024 May 21;21:21. Located at: S1 MEDLINE; 38770686. 10.1089/can.2023.0273 38770686

[170] Mongeau‐Perusse V , Rizkallah E , Morissette F , Brissette S , Bruneau J , Dubreucq S , et al. Cannabidiol Effect on Anxiety Symptoms and Stress Response in Individuals With Cocaine Use Disorder: Exploratory Results From a Randomized Controlled Trial. J Addict Med. 2022 Sep;16(5):521–526. Located at: S1 MEDLINE; 35135986. 10.1097/ADM.0000000000000959 35135986

[171] Morissette F , Mongeau‐Perusse V , Rizkallah E , Thebault P , Lepage S , Brissette S , et al. Exploring cannabidiol effects on inflammatory markers in individuals with cocaine use disorder: a randomized controlled trial. Neuropsychopharmacology. 11 AD. 46(12):2101–2111. Located at: S1 MEDLINE; 34331010. 10.1038/s41386-021-01098-z PMC8505631 34331010

[172] Rizkallah E , Mongeau‐Perusse V , Lamanuzzi L , Castenada‐Ouellet S , Stip E , Juteau LC , et al. Cannabidiol effects on cognition in individuals with cocaine use disorder: Exploratory results from a randomized controlled trial. Pharmacol Biochem Behav 5 AD. 216:173376. Located at: S1 MEDLINE; 35367279. 10.1016/j.pbb.2022.173376 35367279

[173] Montoya ID , Levin FR , Fudala PJ , Gorelick DA . Double‐blind comparison of carbamazepine and placebo for treatment of cocaine dependence. Drug Alcohol Depend. 1995;38(3):213–219 Located at: Embase; 25198287. 10.1016/0376-8716(95)01101-4 PMC2633654 7555621

[174] Montoya ID , Schroeder JR , Preston KL , Covi L , Umbricht A , Contoreggi C , et al. Influence of psychotherapy attendance on buprenorphine treatment outcome. J Subst Abuse Treat. 2005;28(3):247–254. 10.1016/j.jsat.2005.01.004 PMC2633651 15857725

[175] Mooney ME , Schmitz JM , Moeller FG , Grabowski J . Safety, tolerability and efficacy of levodopa‐carbidopa treatment for cocaine dependence: two double‐blind, randomized, clinical trials. Drug Alcohol Depend. 2007;88(2–3):214–223. Located at: Ovid MEDLINE(R) <2005 to 2007>. 10.1016/j.drugalcdep.2006.10.011 PMC2693095 17134849

[176] Mooney ME , Herin DV , Schmitz JM , Moukaddam N , Green CE , Grabowski J . Effects of oral methamphetamine on cocaine use: A randomized, double‐blind, placebo‐controlled trial. Drug Alcohol Depend. 2009;101(1–2):34–41. Located at: Embase; 50352973. 10.1016/j.drugalcdep.2008.10.016 PMC2742691 19058926

[177] Mooney ME , Herin DV , Specker S , Babb D , Levin FR , Grabowski J . Pilot study of the effects of lisdexamfetamine on cocaine use: A randomized, double‐blind, placebo‐controlled trial. Drug Alcohol Depend. 2015;153 ((Mooney, Herin, Specker, Babb, Grabowski] Department of Psychiatry, University of Minnesota, Minneapolis, United States):94–103. 10.1016/j.drugalcdep.2015.05.042 PMC4509923 26116930

[178] Morgan PT , Angarita GA , Canavan S , Pittman B , Oberleitner L , Malison RT , et al. Modafinil and sleep architecture in an inpatient‐outpatient treatment study of cocaine dependence. Drug Alcohol Depend. 2016;160:49–56. 10.1016/j.drugalcdep.2015.12.004 PMC4767553 26777774

[179] Canavan SV , Forselius EL , Bessette AJ , Morgan PT . Preliminary evidence for normalization of risk taking by modafinil in chronic cocaine users. Addict Behav. 2014 Jun;39(6):1057–1061. Located at: S1 MEDLINE; 24642345. 10.1016/j.addbeh.2014.02.015 PMC4026273 24642345

[180] Nanni‐Alvarado R , Gonzalez M , Lima C , Marin‐Navarrete R , Barbosa‐Mendez S , Salazar‐Juarez A . Effect of mirtazapine on craving in cocaine‐dependent patients. International Journal of Mental Health and Addiction. 2022;20(5):2770–2786. Located at: APA PsycInfo <2021>. 10.1007/s11469-021-00547-6

[181] Milivojevic V , Charron L , Fogelman N , Hermes G , Sinha R . Pregnenolone reduces stress‐induced craving, anxiety, and autonomic arousal in individuals with Cocaine use disorder. Biomolecules. 12(11):29. 10.3390/biom12111593 PMC9687893 36358943

[182] Raby WN , Rubin EA , Garawi F , Cheng W , Mason E , Sanfilippo L , et al. A randomized, double‐blind, placebo‐controlled trial of venlafaxine for the treatment of depressed cocaine‐dependent patients. Am J Addict. 2014;23(1):68–75. Located at: Embase; 52656357. 10.1111/j.1521-0391.2013.12065.x PMC4133789 24313244

[183] Noel Raby W , Heller M , Milliaressis D , Jean Choi C , Basaraba C , Pavlicova M , et al. Intranasal oxytocin may improve odds of abstinence in cocaine‐dependent patients: results from a preliminary study. Drug Alcohol Depend Rep. 2022 Mar;2:100016. Located at: S1 MEDLINE; 36845891. 10.1016/j.dadr.2021.100016 PMC9948893 36845891

[184] Nuijten M , Blanken P , Van De Wetering B , Nuijen B , Van Den Brink W , Hendriks VM . Sustained‐release dexamfetamine in the treatment of chronic cocaine‐dependent patients on heroin‐assisted treatment: A randomised, double‐blind, placebo‐controlled trial. Lancet. 2016;387(10034):2226–2234. Located at: Embase; 609202812. 10.1016/S0140-6736(16)00205-1 27015909

[185] Blanken P , Nuijten M , van den Brink W , Hendriks VM . Clinical effects beyond cocaine use of sustained‐release dexamphetamine for the treatment of cocaine dependent patients with comorbid opioid dependence: secondary analysis of a double‐blind, placebo‐controlled randomized trial. Addiction. 2020;115(5):917–923. 10.1111/add.14874 31908066

[186] Oliva A , Reed SC , Brooks DJ , Levin FR , Evans SM . Safety and tolerability of progesterone treatment for women with cocaine use disorder: a pilot treatment trial. Am J Drug Alcohol Abuse. 2022;48(5):586–595. Located at: S1 MEDLINE; 36095308. 10.1080/00952990.2022.2114004 36095308

[187] Mancino MJ , McGaugh J , Feldman Z , Poling J , Oliveto A . Effect of PTSD diagnosis and contingency management procedures on cocaine use in dually cocaine‐ and opioid‐dependent individuals maintained on LAAM: a retrospective analysis. Am J Addict. 2010 Mar;19(2):169–177. Located at: S1 MEDLINE; 20163389. 10.1111/j.1521-0391.2009.00025.x PMC3472706 20163389

[188] Oliveto A , Poling J , Mancino MJ , Feldman Z , Cubells JF , Pruzinsky R , et al. Randomized, double blind, placebo‐controlled trial of disulfiram for the treatment of cocaine dependence in methadone‐stabilized patients. Drug Alcohol Depend. 2011;113(2–3):184–191. Located at: Embase; 51062919. 10.1016/j.drugalcdep.2010.07.022 PMC3005977 20828943

[189] Atkinson TS , Sanders N , Mancino M , Oliveto A . Effects of disulfiram on QTc interval in non‐opioid‐dependent and methadone‐treated cocaine‐dependent patients. J Addict Med. 2013 Jul;7(4):243–248. Located at: S1 MEDLINE; 23648640. 10.1097/ADM.0b013e3182928e02 PMC3737260 23648640

[190] Oliveto A , Poling J , Mancino MJ , Williams DK , Thostenson J , Pruzinsky R , et al. Sertraline delays relapse in recently abstinent cocaine‐dependent patients with depressive symptoms. Addiction. 2012;British Journal of Addiction;107(1):131–141. Located at: APA PsycInfo <2012>. 10.1111/j.1360-0443.2011.03552.x PMC3237722 21707811

[191] Passos SRL , Camacho LAB , Lopes CS , Dos Santos MAB . Nefazodone in out‐patient treatment of inhaled cocaine dependence: a randomized double‐blind placebo‐controlled trial. Addiction. 2005;100(4):489–494 Located at: Embase; 40529841. 10.1111/j.1360-0443.2005.01041.x 15784063

[192] Petrakis IL , Carroll KM , Nich C , Gordon LT , Mccance‐Katz EF , Frankforter T , et al. Disulfiram treatment for cocaine dependence in methadone‐maintained opioid addicts. Addiction. 2000;95(2):219–228. Located at: Embase; 30111242. 10.1046/j.1360-0443.2000.9522198.x 10723850

[193] Jofre‐Bonet M , Sindelar JL , Petrakis IL , Nich C , Frankforter T , Rounsaville BJ , et al. Cost effectiveness of disulfiram: Treating cocaine use in methadone‐maintained patients. J Subst Abuse Treat. 2004 Apr;26(3):225–232. Located at: S1 MEDLINE; 15063917. 10.1016/S0740-5472(04)00004-2 PMC3662046 15063917

[194] Pettinati HM , Kampman KM , Lynch KG , Xie H , Dackis C , Rabinowitz AR , et al. A double blind, placebo‐controlled trial that combines disulfiram and naltrexone for treating co‐occurring cocaine and alcohol dependence. Addict Behav. 2008;33(5):651–667. Located at: Embase; 50014528. 10.1016/j.addbeh.2007.11.011 PMC2374760 18079068

[195] Pettinati HM , Kampman KM , Lynch KG , Dundon WD , Mahoney EM , Wierzbicki MR , et al. A pilot trial of injectable, extended‐release naltrexone for the treatment of co‐occurring cocaine and alcohol dependence. Am J Addict. 2014;23(6):591–597. 10.1111/j.1521-0391.2014.12146.x 25251201

[196] Plebani JG , Lynch KG , Yu Q , Pettinati HM , O'Brien CP , Kampman KM . Results of an initial clinical trial of varenicline for the treatment of cocaine dependence. Drug Alcohol Depend. 2012;121(1–2):163–166. 10.1016/j.drugalcdep.2011.08.025 PMC3262950 21925806

[197] Reid MS , Angrist B , Baker S , Woo C , Schwartz M , Montgomery A , et al. A placebo‐controlled screening trial of celecoxib for the treatment of cocaine dependence. Addiction. 2005;100(SUPPL. 1):32–42. Located at: Embase; 40363423. 10.1111/j.1360-0443.2005.00989.x 15730348

[198] Reid MS , Casadonte P , Baker S , Sanfilipo M , Braunstein D , Hitzemann R , et al. A placebo‐controlled screening trial of olanzapine, valproate, and coenzyme Q10/L‐carnitine for the treatment of cocaine dependence. Addiction. 2005;100(SUPPL. 1):43–57. Located at: Embase; 40363424. 10.1111/j.1360-0443.2005.00990.x 15730349

[199] Reid MS , Angrist B , Baker SA , O'Leary S , Stone J , Schwartz M , et al. A placebo controlled, double‐blind study of mecamylamine treatment for cocaine dependence in patients enrolled in an opiate replacement program. Subst Abuse. 2006;26(2):5–14 Located at: Embase; 44156074. 10.1300/J465v26n02_02 16687365

[200] Santos GM , Ikeda J , Coffin P , Walker JE , Matheson T , McLaughlin M , et al. Pilot study of extended‐release lorcaserin for cocaine use disorder among men who have sex with men: A double‐blind, placebo‐controlled randomized trial. PLoS ONE. 2021;16(7) Located at: APA PsycInfo <2021>:e0254724. 10.1371/journal.pone.0254724 PMC8282062 34265007

[201] Schmitz JM , Averill P , Stotts AL , Moeller FG , Rhoades HM , Grabowski J . Fluoxetine treatment of cocaine‐dependent patients with major depressive disorder. Drug Alcohol Depend. 2001;63(3):207–214. Located at: Ovid MEDLINE(R) <1996 to 2002>. 10.1016/s0376-8716(00)00208-8 11418225

[202] Schmitz JM , Mooney ME , Moeller FG , Stotts AL , Green C , Grabowski J . Levodopa pharmacotherapy for cocaine dependence: Choosing the optimal behavioral therapy platform. Drug Alcohol Depend. 2008;94(1–3):142–150. Located at: Embase; 50025645. 10.1016/j.drugalcdep.2007.11.004 PMC2293271 18164144

[203] Schmitz JM , Lindsay JA , Green CE , Herin DV , Stotts AL , Gerard Moeller F . High‐dose naltrexone therapy for cocaine‐alcohol dependence. Am J Addict. 2009;18(5):356–362. Located at: Embase; 355585690. 10.1080/10550490903077929 PMC2907651 19874153

[204] Schmitz JM , Rathnayaka N , Green CE , Moeller FG , Dougherty AE , Grabowski J . Combination of modafinil and d‐amphetamine for the treatment of cocaine dependence: A preliminary investigation. Front Psychiatry. 2012;3(AUG):77. Located at: Embase; 365742963. 10.3389/fpsyt.2012.00077 PMC3430875 22969732

[205] Schmitz JM , Green CE , Stotts AL , Lindsay JA , Rathnayaka NS , Grabowski J , et al. A two‐phased screening paradigm for evaluating candidate medications for cocaine cessation or relapse prevention: Modafinil, levodopa‐carbidopa, naltrexone. Drug Alcohol Depend. 2014;136(1):100–107. Located at: Embase; 52952427. 10.1016/j.drugalcdep.2013.12.015 PMC3944935 24424425

[206] Schmitz JM , Green CE , Hasan KM , Vincent J , Suchting R , Weaver MF , et al. PPAR‐gamma agonist pioglitazone modifies craving intensity and brain white matter integrity in patients with primary cocaine use disorder: a double‐blind randomized controlled pilot trial. Addiction. 2017;112(10):1861–1868. Located at: Embase; 622409442. 10.1111/add.13868 PMC5593771 28498501

[207] Schmitz JM , Suchting R , Green CE , Webber HE , Vincent J , Moeller FG , et al. The effects of combination levodopa‐ropinirole on cognitive improvement and treatment outcome in individuals with cocaine use disorder: A Bayesian mediation analysis. Drug Alcohol Depend. 2021;225:108800. 10.1016/j.drugalcdep.2021.108800 34102508

[208] Schmitz JM , Stotts AL , Vujanovic AA , Yoon JH , Webber HE , Lane SD , et al. Contingency management plus acceptance and commitment therapy for initial cocaine abstinence: Results of a sequential multiple assignment randomized trial (SMART). Drug Alcohol Depend. 2024 Mar;256:111078. Located at: S1 MEDLINE; 38309089. 10.1016/j.drugalcdep.2023.111078 38309089

[209] Schubiner H , Saules KK , Arfken CL , Johanson C‐E , Schuster CR , Lockhart N , et al. Double‐blind placebo‐controlled trial of methylphenidate in the treatment of adult ADHD patients with comorbid cocaine dependence. Exp Clin Psychopharmacol. 2002;10(3):286–294. Located at: Embase; 34919937. 10.1037/1064-1297.10.3.286 12233989

[210] Shearer J , Wodak A , Van Beek I , Mattick RP , Lewis J . Pilot randomized double blind placebo‐controlled study of dexamphetamine for cocaine dependence. Addiction. 2003;98(8):1137–1141. Located at: Embase; 36936500. 10.1046/j.1360-0443.2003.00447.x 12873248

[211] Shoptaw S , Kintaudi PC , Charuvastra C , Ling W . A screening trial of amantadine as a medication for cocaine dependence. Drug Alcohol Depend. 2002;66(3):217–224. Located at: Embase; 34327857. 10.1016/S0376-8716(01)00205-8 12062456

[212] Shoptaw S , Yang X , Rotheram‐Fuller EJ , Hsieh Y‐CM , Kintaudi PC , Charuvastra VC , et al. Randomized placebo‐controlled trial of baclofen for cocaine dependence: Preliminary effects for individuals with chronic patterns of cocaine use. J Clin Psychiatry. 2003;64(12):1440–1448. Located at: Embase; 41270102. 10.4088/JCP.v64n1207 14728105

[213] Shoptaw S , Majewska MD , Wilkins J , Twitchell G , Yang X , Ling W . Participants receiving dehydroepiandrosterone during treatment for cocaine dependence show high rates of cocaine use in a placebo‐controlled pilot study. Exp Clin Psychopharmacol. 2004;12(2):126–135. 10.1037/1064-1297.12.2.126 15122957

[214] Shoptaw S , Watson DW , Reiber C , Rawson RA , Montgomery MA , Majewska MD , et al. Randomized controlled pilot trial of cabergoline, hydergine and levodopa/carbidopa: Los Angeles Cocaine Rapid Efficacy Screening Trial (CREST). Addiction. 2005;100(SUPPL. 1):78–90. Located at: Embase; 40363427. 10.1111/j.1360-0443.2005.00991.x 15730352

[215] Shorter D , Lindsay JA , Kosten TR . The alpha‐1 adrenergic antagonist doxazosin for treatment of cocaine dependence: A pilot study. Drug Alcohol Depend. 2013 Jul 1;131(1):66–70. 10.1016/j.drugalcdep.2012.11.021 PMC3655111 23306096

[216] Sofuoglu M , Poling J , Gonzalez G , Gonsai K , Oliveto A , Kosten TR . Progesterone Effects on Cocaine Use in Male Cocaine Users Maintained on Methadone: A Randomized, Double‐Blind, Pilot Study. Exp Clin Psychopharmacol. 2007;15(5):453–460. Located at: Embase; 350102232. 10.1037/1064-1297.15.5.453 17924779

[217] Sofuoglu M , Carroll KM . Effects of galantamine on cocaine use in chronic cocaine users. Am J Addict. 2011 May;20(3):302–303. Located at: S1 MEDLINE; 21477060. 10.1111/j.1521-0391.2011.00130.x PMC3073598 21477060

[218] Sofuoglu M , Poling J , Babuscio T , Gonsai K , Severino K , Nich C , et al. Carvedilol does not reduce cocaine use in methadone‐maintained cocaine users. J Subst Abuse Treat. 2017;73:63–69. 10.1016/j.jsat.2016.11.005 PMC5193370 28017186

[219] Somoza EC , Winship D , Gorodetzky CW , Lewis D , Ciraulo DA , Galloway GP , et al. A multisite, double‐blind, placebo‐controlled clinical trial to evaluate the safety and efficacy of vigabatrin for treating cocaine dependence. JAMA Psychiatry. 2013;70(6):630–637. 10.1001/jamapsychiatry.2013.872 23575810

[220] Berezina TL , Khouri AS , Winship MD , Fechtner RD . Visual field and ocular safety during short‐term vigabatrin treatment in cocaine abusers. Am J Ophthalmol. 2012 Aug;154(2):326–332.e2. Located at: S1 MEDLINE; 22704138. 10.1016/j.ajo.2012.02.026 22704138

[221] Stine SM , Krystal JH , Kosten TR , Charney DS . Mazindol treatment for cocaine dependence. Drug Alcohol Depend. 1995;39(3):245–252. Located at: Embase; 25309396. 10.1016/0376-8716(95)01174-4 8556974

[222] Suchting R , Green CE , de Dios C , Vincent J , Moeller FG , Lane SD , et al. Citalopram for treatment of cocaine use disorder: A Bayesian drop‐the‐loser randomized clinical trial. Drug Alcohol Depend. 2021;228:109054. Located at: S1 MEDLINE; 34600245. 10.1016/j.drugalcdep.2021.109054 PMC8595787 34600245

[223] de Dios C , Suchting R , Green CE , Webber HE , Moeller FG , Lane SD , et al. The role of Iowa gambling task performance in response to citalopram treatment for cocaine use disorder. Am J Drug Alcohol Abuse. 2025 Jan 24;1–12. Located at: S1 MEDLINE; 39853194. 10.1080/00952990.2024.2420773 PMC11899305 39853194

[224] Tapp A , Wood AE , Kennedy A , Sylvers P , Kilzieh N , Saxon AJ . Quetiapine for the treatment of cocaine use disorder. Drug Alcohol Depend. 2015;149 ((Tapp) VA Puget Sound Health Care System, American Lake Division (A‐116), University of Washington, 9600 Veterans Drive SW, Tacoma, WA 98493, United States):18–24. 10.1016/j.drugalcdep.2014.12.037 25682480

[225] Umbricht A , DeFulio A , Winstanley EL , Tompkins DA , Peirce J , Mintzer MZ , et al. Topiramate for cocaine dependence during methadone maintenance treatment: A randomized controlled trial. Drug Alcohol Depend. 2014;140:92–100. 10.1016/j.drugalcdep.2014.03.033 PMC4431633 24814607

[226] Rass O , Umbricht A , Bigelow GE , Strain EC , Johnson MW , Mintzer MZ . Topiramate impairs cognitive function in methadone‐maintained individuals with concurrent cocaine dependence. Psychol Addict Behav. 2015 Mar;29(1):237–246. Located at: S1 MEDLINE; 25365653. 10.1037/adb0000027 PMC4388752 25365653

[227] Walsh SL , Middleton LS , Wong CJ , Nuzzo PA , Campbell CL , Rush CR , et al. Atomoxetine does not alter cocaine use in cocaine dependent individuals: A double blind randomized trial. Drug Alcohol Depend. 2013;130(1–3):150–157. Located at: Embase; 52326966. 10.1016/j.drugalcdep.2012.10.024 PMC4852978 23200303

[228] Wardle MC , Vincent JN , Suchting R , Green CE , Lane SD , Schmitz JM . Anhedonia is associated with poorer outcomes in contingency management for cocaine use disorder. J Subst Abuse Treat. 2017;72:32–39. 10.1016/j.jsat.2016.08.020 PMC5154907 27646197

[229] Ware OD , Sweeney MM , Cunningham C , Umbricht A , Stitzer M , Dunn KE . Bupropion Slow Release vs Placebo With Adaptive Incentives for Cocaine Use Disorder in Persons Receiving Methadone for Opioid Use Disorder: A Randomized Clinical Trial. JAMA Netw Open. 2023 Mar 15;6(3):e232278. 10.1001/jamanetworkopen.2023.2278 PMC10018324 36920397

[230] Winhusen TM , Somoza EC , Harrer JM , Mezinskis JP , Montgomery MA , Goldsmith RJ , et al. A placebo‐controlled screening trial of tiagabine, sertraline and donepezil as cocaine dependence treatments. Addiction. 2005 Mar;100(Suppl 1):68–77. Located at: S1 MEDLINE; 15730351. 10.1111/j.1360-0443.2005.00992.x 15730351

[231] Winhusen T , Somoza E , Sarid‐Segal O , Goldsmith RJ , Harrer JM , Coleman FS , et al. A double‐blind, placebo‐controlled trial of reserpine for the treatment of cocaine dependence. Drug Alcohol Depend. 2007;91(2):205–212. 10.1016/j.drugalcdep.2007.05.021 PMC10556850 17628352

[232] Winhusen TM , Lewis DF , Somoza EC , Horn P . Pharmacodynamics must inform statistics: An example from a cocaine dependence pharmacotherapy trial. ISRN Addict. 2014;2014:927290. 10.1155/2014/927290 PMC4392966 25969833

[233] Winhusen T , Somoza E , Ciraulo DA , Harrer JM , Goldsmith RJ , Grabowski J , et al. A double‐blind, placebo‐controlled trial of tiagabine for the treatment of cocaine dependence. Drug Alcohol Depend. 2007;91(2–3):141–148. Located at: Embase; 47554604. 10.1016/j.drugalcdep.2007.05.028 17629631

[234] Winhusen TM , Kropp F , Lindblad R , Douaihy A , Haynes L , Hodgkins C , et al. Multisite, randomized, double‐blind, placebo‐controlled pilot clinical trial to evaluate the efficacy of buspirone as a relapse‐prevention treatment for cocaine dependence. J Clin Psychiatry. 2014;75(7):757–764. 10.4088/JCP.13m08862 PMC4125613 24911028

[235] Winstanley EL , Bigelow GE , Silverman K , Johnson RE , Strain EC . A randomized controlled trial of fluoxetine in the treatment of cocaine dependence among methadone‐maintained patients. J Subst Abuse Treat. 2011;40(3):255–264. Located at: Embase; 51245446. 10.1016/j.jsat.2010.11.010 PMC3078567 21266301

[236] Zhang X , Nielsen DA , Domingo CB , Shorter DI , Nielsen EM , Kosten TR . Pharmacogenetics of *Dopamine β‐Hydroxylase* in cocaine dependence therapy with doxazosin. Addict Biol. 2019 May;24(3):531–538. 10.1111/adb.12611 PMC6119656 29498170

[237] Shorter DI , Zhang X , Domingo CB , Nielsen EM , Kosten TR , Nielsen DA . Doxazosin treatment in cocaine use disorder: pharmacogenetic response based on an alpha‐1 adrenoreceptor subtype D genetic variant. Am J Drug Alcohol Abuse. 2020;46(2):184–193. Located at: S1 MEDLINE; 31914324. 10.1080/00952990.2019.1674864 31914324

[238] Spiga F , Parkhouse T , Tang VM , Savović J , Foll BL , Nielsen S . Pharmacotherapies for cannabis use disorder. Cochrane Database Syst Rev [Internet]. 2025;(9):CD008940. 10.1002/14651858.CD008940.pub4 PMC12481667 41025421

[239] Brandt L , Chao T , Comer SD , Levin FR . Pharmacotherapeutic strategies for treating cocaine use disorder—what do we have to offer? Addiction. 2021;116(4):694–710. 10.1111/add.15242 PMC7930140 32888245

[240] Pani PP , Trogu E , Vecchi S , Amato L . Antidepressants for cocaine dependence and problematic cocaine use. Cochrane Database Syst Rev https://www.cochranelibrary.com/cdsr/doi/10.1002/14651858.CD002950.pub3/full. 2011;(12):CD002950. 10.1002/14651858.CD002950.pub3 PMC12277681 22161371

[241] Lassi DLS , Malbergier A , Negrao AB , Florio L , De Aquino JP , Castaldelli‐Maia JM . Pharmacological treatments for cocaine craving: what is the way forward? A systematic review. Brain Sci. 2022;12(11):1546. 10.3390/brainsci12111546 PMC9688748 36421870

[242] Jiménez‐Silvestre K , Rodríguez‐Kuri SE , Fernández‐Cáceres C , Marín‐Navarrete R . Comorbidities in patients with cocaine use disorders. J Subst Use. 2024 https://www.tandfonline.com/doi/abs/10.1080/14659891.2024.2444217;30(6):951–957.

[243] Palamar JJ , Davies S , Ompad DC , Cleland CM , Weitzman M . Powder cocaine and crack use in the United States: An examination of risk for arrest and socioeconomic disparities in use. Drug Alcohol Depend. 2015;149:108–116. 10.1016/j.drugalcdep.2015.01.029 PMC4533860 25702933

[244] Kirchoff RW , Mohammed NM , McHugh J , Markota M , Kingsley T , Leung J , et al. Naltrexone initiation in the inpatient setting for alcohol use disorder: A systematic review of clinical outcomes. Mayo Clin Proc Innov Qual Outcomes. 2021;5(2):495–501. 10.1016/j.mayocpiqo.2021.01.013 PMC8105524 33997645

